# Transition metal-free hydrogenative coupling of nitroarenes mediated with dihydropyridine: chemoselective formation of aromatic azoxy, azo, hydrazine and phenazine

**DOI:** 10.1039/d5ra04782j

**Published:** 2025-09-15

**Authors:** Chuang Lu, Dejun Zhou, Yangqi Zhang, Siye Du, Qiaomei Zheng, Di Wu, Weixin Zheng

**Affiliations:** a College of Material, Chemistry & Chemical Engineering, Hangzhou Normal University Hangzhou 311121 China wxzheng@hznu.edu.cn

## Abstract

Due to the diversity of the compounds containing dinitrogen bonds, chemoselectivity is a key issue to be addressed for the hydrogenative coupling of nitroarenes. A system consisting of Hantzsch 1,4-dihydropyridine (HEH) and a base was developed as a transition metal-free reductant for the hydrogenative coupling of nitroarenes to provide aromatic azoxy, azo and hydrazine. Under optimized conditions, the reaction of 2-fluoronitroarene afforded phenazines. Chemoselectivity for the formation of these dinitrogen compounds was effectively regulated by the choice of the base and the amount of reductant employed. A plausible free-radical mechanism for the hydrogenative coupling of nitroarenes was proposed, wherein the combination of HEH and NaH acted as a synergistic reductant.

## Abstract 2

A metal-free reductant, 1,4-dihydropyridine/base system, enabled for the hydrogenative coupling of nitroarenes. the chemoselectivity of dinitrogen product was regulated by base choice and reductant amount.

## Graphical abstract

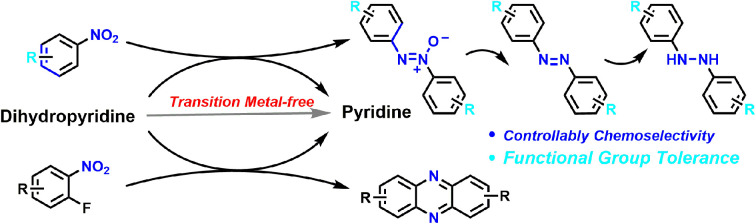

## 1 Introduction

Dinitrogen linkage-containing compounds, including azoxy, azo and hydrazine, have been widely considered for their extensive applications. The N=N moiety is usually found in pharmaceuticals,^1^ food additives,^2^ dyes, pigments,^3^ some advanced materials^4^ and numerous natural products.^5^ Extrusion of dinitrogen from diazene^6^ could generate carbon-based biradicals for the construction of new chemical bonds.^7^ Diazene oxides act as a 1,3-dipole skeleton in cyclization to produce various heterocyclic derivatives.^8^ Diaromatic hydrazines are vital intermediates in the preparation of antitumor agents and antimicrobial compounds.^9^ Their ability to form stable complexes with metal ions also makes them useful in the development of metal-based drugs.^10^ All the above properties have stimulated great interest in developing synthetic methods with effectivity and selectivity.

**Scheme 1:**
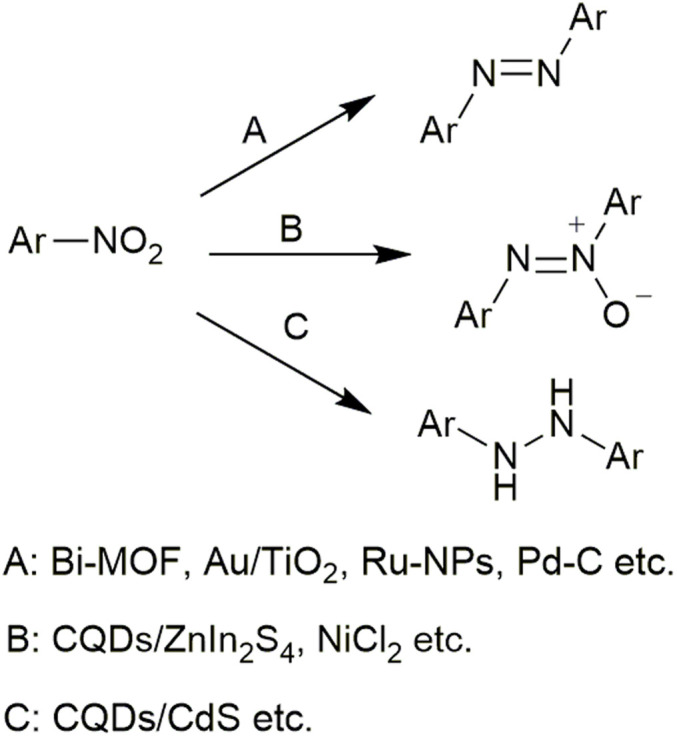
Synthesis of dinitrogen linkage-containing compounds *via* transition-metal-catalyzed nitro reductive coupling reactions.

From a synthetic perspective, reductive coupling of aromatic nitro compounds associated with transition metal reagents represents an effective approach for the construction of dinitrogen frameworks. Although chemoselectivity of the Béchamp reduction is remarkably high,^11,12^ neither the use of stoichiometric amounts of metals or metal salts nor the generation of metal oxide as by-products could be ignored. Various transition metal-catalysts, such as Bi,^13,14^ Au,^15–17^ Ru,^18–20^ Pd,^21–23^ In,^24,25^ Ni,^26–28^ and Cd,^29,30^ have been utilized for the reduction of aromatic nitro compounds to produce structurally diverse N-containing products (Scheme 1). Since metallic reagents play a role in activating covalent bonds in the presence of a hydrogen source, most catalyzed reductions yield a single product. Contrastively, the selective production of multiple products from the same reduction system has been rarely observed. Ruthenium nanoparticle-catalyzed reduction of nitroarenes gives azoxyarenes, azoarenes, or anilines using ethanol as a hydrogen source.^16^ Formation of azoxybenzene or azobenzene can be adjusted *via* light irradiation over Au/CeO_2_ photocatalysts.^31^ In most cases, the hydrogenation process requires high temperatures, specialized high-pressure equipment^32^ and relatively harsh reaction conditions. Moreover, some functional groups that are sensitive to reductive hydrogenation, such as cyano, carbonyl and halogen, are not compatible during the transition metal-catalyzed reduction of nitroarenes. Such limited compatibility constrains the diversification of the products.

With growing emphasis on the environmental credentials, synthetic strategies that avoid transition-metal catalysts have become a highly prominent research area in the past few decades.^33^ As to the reduction of the nitro group, the control over the product selectivity is not to be neglected due to the diversity of the N-containing products. An ethanol/sodium hydroxide system was used as the first non-transition-metal reductant for nitroarenes to generate azobenzene, where ethanol was the hydride donor.^34^ Nitrobenzene can be selectively converted to the corresponding azoxybenzene or aniline when isopropanol or *n*-propanol is used as the hydrogen source, respectively.^35^ Involving hydrazine as the reductant, the photo-induced reduction of nitroarenes can selectively generate *N*-arylhydroxylamines or azoxybenzene through the structural regulation of hydrazine.^36^ Compared with the more developed transition metal catalytic system, the non-transition metal-mediated nitro reduction system still has significant limitations, especially the lack of product regulation ability. This key problem leads to difficulty in achieving selective synthesis of multiple products in the same reaction system. Therefore, it is of great value to find an efficient non-metallic reductant to promote the selective reduction of nitro compounds into multiple products.

Hantzsch 1,4-dihydropyridine (HEH) is a partially saturated six-membered ring with two carbon–carbon double bonds at the 2nd and 5th positions. HEH acts as a classical biomimetic hydrogen transfer agent^37^ and has wide applicability in a variety of reductive reactions.^38^ In transition-metal-catalyzed reactions, HEH behaves as a hydride source for the hydrogenative reduction.^39^ For instance, hydrogenation of azides generates amines when catalyzed by Pd/C.^40^ Imine is hydrogenated with ruthenium catalysts.^41^ HEH can act as the reductant in the Sc(OTf)_3_-promoted reductive amination of ketones.^42^ The C=C double bonds in α,β-unsaturated ketones are selectively reduced by dihydropyridine.^43^ Besides the metal-catalyzed cases, HEH can also work as a hydride donor in the several transition metal-free systems, such as the reduction of imine to amine^44^ and the chemoselective hydrogenation of C=C double bond in coumarin lactone.^32^ Since the concepts of sustainable development and production have become key focuses, the chemical processes avoiding the use of transition metals have become increasingly attractive than before. In some cases, metal-free processes demonstrate better functional group tolerance.^45^ Moreover, the dominance of selectivity and reactivity should be the key point when comparing with metal-catalyzed processes.

Herein, given the limited exploration of metal-free systems for the hydrogenative coupling of aromatic nitro compounds, a reductant system involving HEH and a base was developed to produce aromatic azoxy, azo, hydrazine and phenazine chemoselectively.

## 2 Results and discussion

### 2.1 Synthesis of aromatic azoxy

Reduction of *p*-nitrotoluene (1a) was examined as a modular reaction to produce azoxytoluene (2a) using HEH as the reductant under alkaline conditions (Table 1).

**Table 1:** Reduction of *p*-nitrotoluene 1a for the synthesis of azoxytoluene 2a

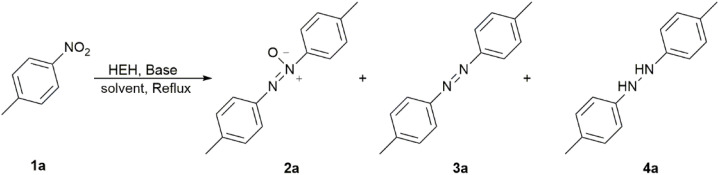									
Entry^a^	Solvents	HEH (equiv.)	Base/(equiv.)	Time (h)	Proportion 2a : 3a : 4a	Conversion (%)	Yield^b^ (%)		
							2a	3a	4a
1	CH_3_CN	2	NaOH/(4)	12	98 : 2 : 0	70	62	1	—
2	CH_3_CN	3	NaOH/(4)	2	96 : 4 : 0	94	90	4	—
3	CH_3_CN	3	NaOH/(5)	2	91 : 1 : 8	92	70	1	6
4	CH_3_CN	4	NaOH/(4)	2	97 : 3 : 0	94	74	2	—
5	CH_3_CN	4	NaOH/(5)	2	91 : 5 : 4	100	68	3	4
**6**	**CH**_**3**_ **CN**	**3**	**NaOH/(3)**	**3**	**>99 : 1 : 0**	**100**	**89**	**1**	**—**
7^c^	CH_3_CN	3	NaOH/(3)	1.5	97 : 3 : 0	100	87	3	—
8	CH_3_CN	3	K_2_CO_3_/(3)	3	—	Trace	—	—	—
9	CH_3_CN	3	*t*-BuOK/(3)	1	—	—	—	—	—
10	CH_3_CN	3	NaH/(3)	2	70 : 30 : 0	96	66	27	—
11	Acetone	3	NaOH/(3)	4	48 : 0 : 0	56	48	—	—
12	DMF	3	NaOH/(3)	4	11 : 0 : 0	15	11	—	—
13	CH_3_OH	3	NaOH/(3)	3	—	Trace	—	—	—

a Reaction conditions: 1.0 mmol of 1a was involved in CH_3_CN under refluxing.

b ^1^H NMR yield using dibromomethane as the internal standard.

c N_2_ atmosphere.

**Scheme 2:**
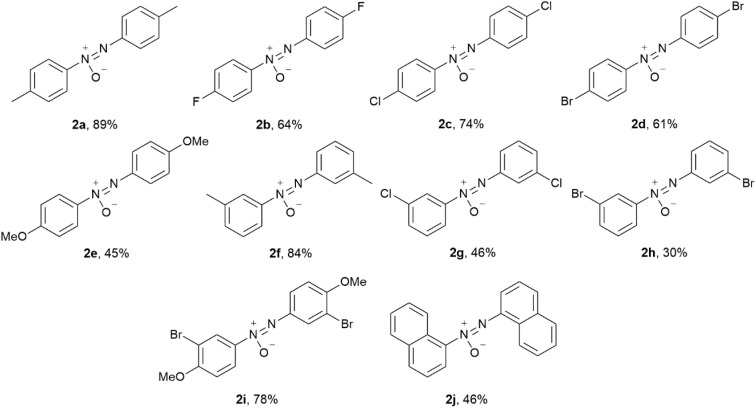
Synthesis of aromatic azoxy derivatives 2a–j.

The amount of HEH and the type of base had significant influence on the chemoselectivity of the products. NaOH was proven to be an effective base for the formation of the azoxytoluene (Table 1, entries 1–7). 2 equivalents of HEH with 4 equivalents of NaOH gave a reasonable chemoselectivity for 2a, but the substrate 1a was not consumed completely in 12 hours (Table 1, entry 1). Keeping the amount of NaOH constant, increasing HEH by 1 equiv. reduced the reaction time to 2 hours, and the conversion of the substrate was raised to 94% (Table 1, entry 2). Increasing the amount of HEH magnified the conversion, but the selectivity for the products was decreased (Table 1, entries 2–5). When both HEH and NaOH were used at 3 equivalents, the highly selective product 2a could be obtained in 3 hours (Table 1, entry 6). Inert nitrogen atmosphere significantly accelerated the reaction process, but the ratio of azo 3a increased slightly (Table 1, entry 7). Alkali source replacement experiments (such as K_2_CO_3_ and *t*-BuOK) failed to generate the target product 2a (Table 1, entries 8–9). Notably, the introduction of sodium hydride increased the yield of the azo product 3a (Table 1, entry 10). This result suggested a potential strategy for modulating reaction selectivity toward azoarene formation. The control of solvents (acetone, DMF, and methanol) did not achieve the desired results (Table 1, entries 11–13). Thus, 3 equivalents of both HEH and NaOH in acetonitrile could be used for the preparation of azoxytoluene (Table 1, entry 6). Based on the optimized conditions in Table 1, the substituents in nitroarenes were screened (Scheme 2).

**Scheme 3:**
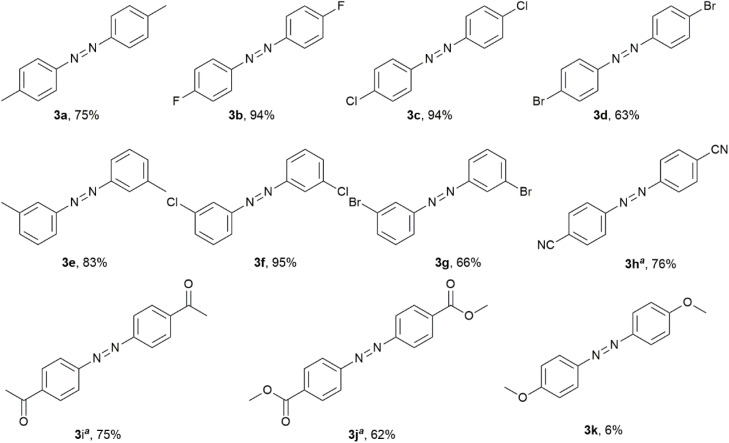
Synthesis of azoarene derivatives 3a–k.

Based on the results in Scheme 2, weak electron-withdrawing (EW) effects favored the formation of azoxy (2b–d). Electron-withdrawing groups (EWGs) could provide a positive effect on the formation of azoxybenzene compared with electron-donating groups (EDGs) (2e). The position of substituents profoundly affected the reactivity: *para*-substituted substrates exhibited higher yields, while *ortho*-substituted substrates hardly reacted due to the steric hindrance. The yields of both *para*-chloro (2c) and -bromo (2d) substrates were higher than those of their corresponding *meta*-substituted products. NMR analysis of the crude product of 2g and 2h revealed that the major products were the corresponding azo products in >60% NMR yield, rather than the azoxy 2g and 2h. When Cl and Br were attached to the *meta*-position of the nitro group, the enhanced EW effects of substituents drove the reaction toward further reduction to generate the azo compounds. For the EW conjugative effect, the *para*-carbonyl- and *para*-cyano-substituted nitroarenes were reduced beyond the azoxy stage, affording only azoarenes (Scheme 3, 3h–j). Such an unexpected finding and the case of entry 10 in Table 1 suggested the possibility that the HEH-base system would be applicable to the synthesis of azo compounds. Due to the combined action of the electronic effect of EWGs and the inductive effect of *meta* position, the yield of *m*-chloro substrate (46%, 2g) was slightly higher than that of *m*-bromo substrate (30%, 2h). Notably, the system maintained high efficiency (78%, 2i) even when both EWGs and EDGs coexisted on the aromatic ring, demonstrating excellent functional group tolerance. The results in Table 2 fully confirmed the selective reduction characteristics of the HEH–NaOH system for the conversion of nitroarenes to azoxyarenes.

**Table 2:** Reduction of *p*-nitrotoluene 1a for the synthesis of azotoluene 3a

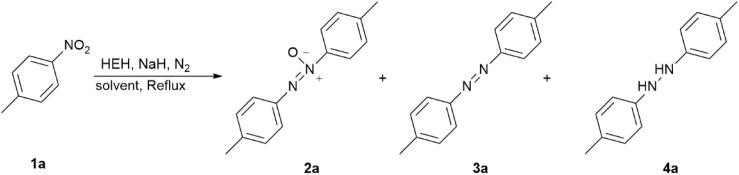								
Entry^a^	HEH (equiv.)	NaH (equiv.)	Time (h)	Proportion 2a : 3a : 4a	Conversion (%)	Yield^b^ (%)		
						2a	3a	4a
1	2	2	1.5	97 : 3 : 0	98	75	2	—
2	2	4	1.5	36 : 64 : 0	100	20	35	—
3	2	6	1.5	45 : 55 : 0	100	29	35	—
4	2	8	1.5	44 : 56 : 0	100	24	30	—
5	2	10	1.5	13 : 87 : 0	100	6	39	—
6	3	4	1	0 : 10 : 90	100	0	10	90
7	2.1	10	3	2 : 98 : 0	100	1	62	—
8	2.1	6	3	17 : 83 : 0	100	9	44	—
9	2.2	6	3	0 : 97 : 3	100	—	74	2
10	2.3	6	3	0 : 69 : 31	100	—	44	20
**11**	**2.2**	**5**	**3**	**0 : 99 : 1**	**100**	**—**	**75**	**1**

a Reaction conditions: 1.0 mmol of 1a was involved in CH_3_CN under reflux.

b ^1^H NMR yield using dibromomethane as the internal standard.

### 2.2 Synthesis of aromatic azo and hydrazine

For further research on the modulation of the selective diversification of nitroarene reduction, sodium hydride was applied instead of sodium hydroxide as a base in a nitrogen atmosphere for the selective generation of azo compounds owing to the case of entry 10 in Table 1. When 2 equivalents of HEH were used, the ratio of azotoluene 3a was promoted with the addition of increasing amounts of sodium hydride (Table 2, entries 1–5). However, the selectivity was not acceptable for effective preparation. The addition of an extra one equivalent of HEH led to the formation of hydrazine 4a, skipping the intermediate oxidation state of azotoluene 3a (Table 2, entry 6). These findings provided evidence that the dosage of HEH needed be controlled more precisely to achieve efficient nitro-to-azo conversion (Table 2, entries 7–11). Ultimately, using 2.2 equivalents of HEH with 5.0 equivalents of sodium hydride (Table 2, entry 11) achieved reasonable selectivity for azo 3a, demonstrating the need for a critical balance between the reductant and base.

The optimized conditions for azo 3a (Table 2, entry 11) worked in all the listed cases of *ortho*- and *meta*-substituted nitroarenes to form the corresponding azo 3a–k in good yields (Scheme 3). Some sensitive functional groups in transition metal-catalyzed reactions, such as halogen, cyano and carbonyl groups, were tolerated under these conditions. In the cases of 3h–j, both conditions of HEH–NaOH (Table 1, entry 6) and HEH–NaH (Table 2, entry 11) worked and the azo yields in the former were higher. For the electron-donating conjugation of the oxygen atom in the methyloxy group, increasing the electron density on the aromatic moiety hindered the formation of the azo (Scheme 3, 3k in 6% yield).

The case of entry 6 in Table 2 reminded us that the combination of HEH and NaH could be used for the synthesis of hydrazine. Based on the optimization of reagent stoichiometry, 4 equivalents each of HEH and NaH favored the formation of ditolyl hydrazine 4a in 91% NMR yield and >99% of isomer ratio. It was indicated that the combination of HEH and NaH had the dual ability to produce azoarene and aromatic hydrazine. As hydrazine was readily oxidized to azo in air during the workup, NMR yields of 4a–d were given to demonstrate the efficiency of HEH–NaH in the synthesis of hydrazine. In order to further confirm the formation of diaromatic hydrazine, 3-chloropropionyl chloride was introduced as an *in situ* trapping agent to derivatize hydrazine upon completion of the coupling reduction. The corresponding condensation products, pyrazolones 5a–d, were successfully obtained in good yields (Table 3). These results verified the formation of aromatic hydrazines 4a–d.

**Table 3:** Synthesis and capture of aromatic hydrazines

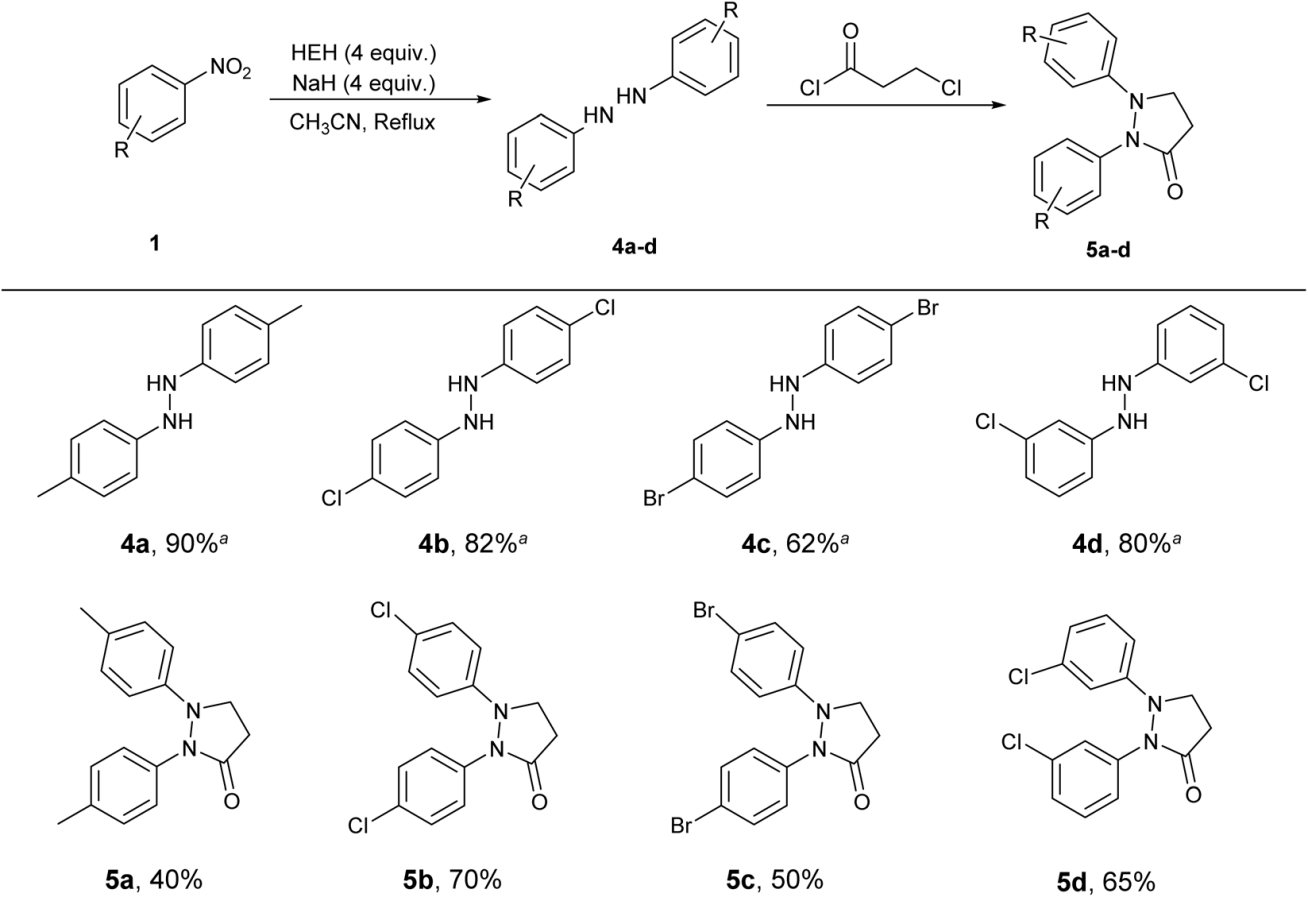

a ^1^H NMR yield using dibromomethane as internal standard.

### 2.3 Mechanism

**Scheme 4:**
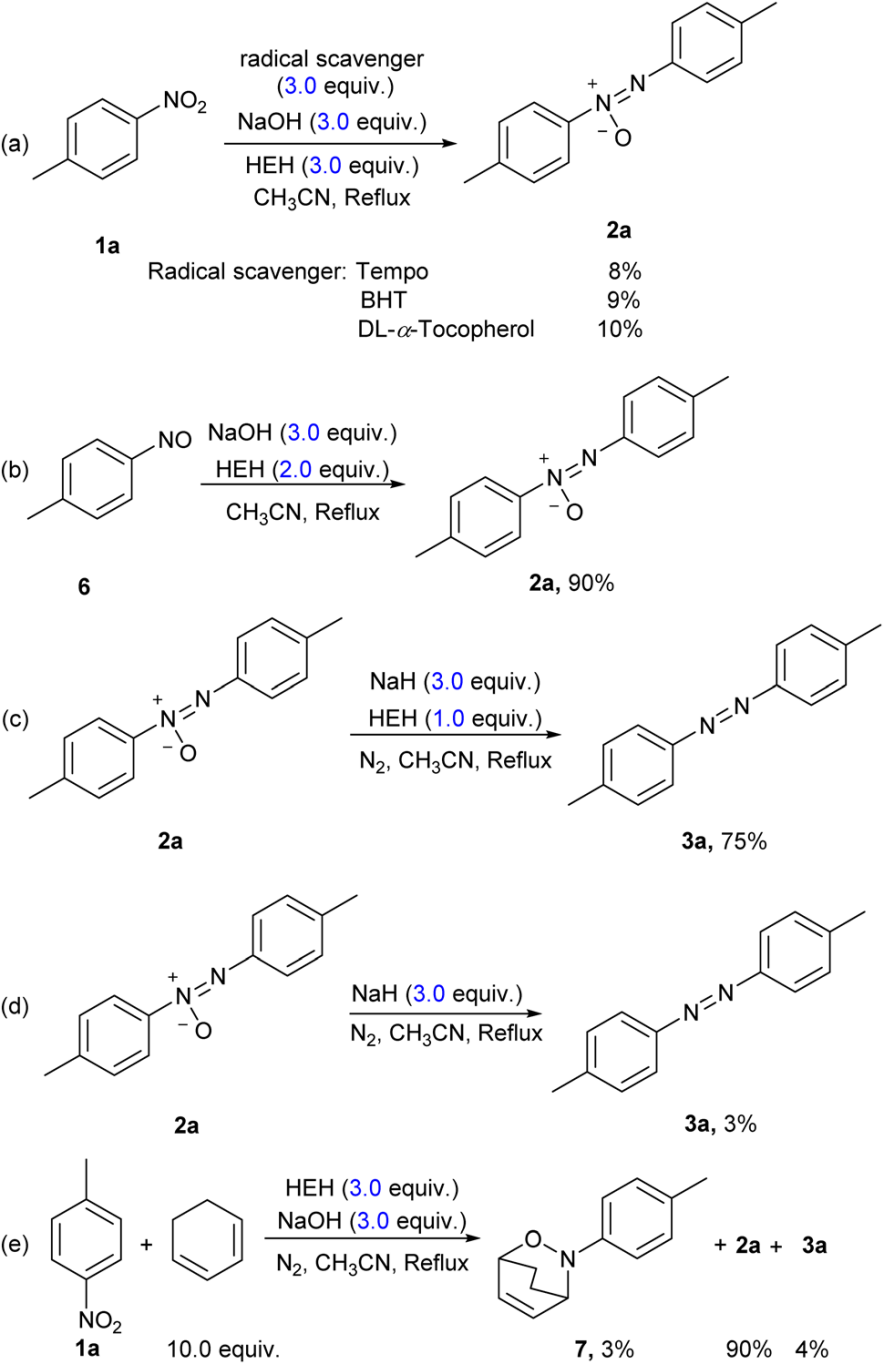
Control experiments.

In order to clarify the mechanism of hydrogenative coupling of nitroarene mediated with the HEH-base system, several control experiments were carried out, as shown in Scheme 4.

Free radical trapping agents, tetramethylammonium hydroxide (TEMPO), butylated hydroxytoluene (BHT) and dl-α-tocopherol, were used respectively to verify the mechanism properties of the reaction under the conditions of entry 6 in Table 1. With the addition of the radical scavengers, reductions of nitrotoluene were hindered, and the yields of the azoxytoluene 2a had plummeted (Scheme 4, eqn (a)). These results indicated that the reduction of aromatic nitro groups mediated by HEH-base underwent a free radical mechanism. Based on the hypothesis of the bimolecular reduction of nitroarene, nitrosyltoluene 6 was treated as a substrate in the presence of 2 equiv. of HEH, and the target product 2a was successfully obtained in the yield of 90% (Scheme 4, eqn (b)). With 1 equiv. of HEH, reduction of azoxytoluene 2a afforded azo compound 3a in 75% yield (Scheme 4, eqn (c)). Addition of NaH to azoxytoluene 2a in acetonitrile could generate trace azobenzene in the absence of HEH (Scheme 4, eqn (d)). It was supposed that sodium hydride had a chemical effect in this process. These results could explain why the amount of HEH required for the generation of the low-oxidation-state azo compound was lesser than that consumed by the azoxy compound. NaH in such a process could provide part of the hydrogen source for the reduction reaction. Therefore, the combination of HEH and NaH worked as a synergistic reductant. For further evidence for the intermediate, 10 equivalents of cyclohexa-1,3-diene was involved as the capturer of the intermediate of nitrosotoluene, a dienophile, and the corresponding product 7 was separated in the yield of 3% (Scheme 4, eqn (e)). The generation of 7 provided a hard evidence for the formation of nitroso intermediate in the process of the reduction of the aromatic nitro group.

**Scheme 5:**
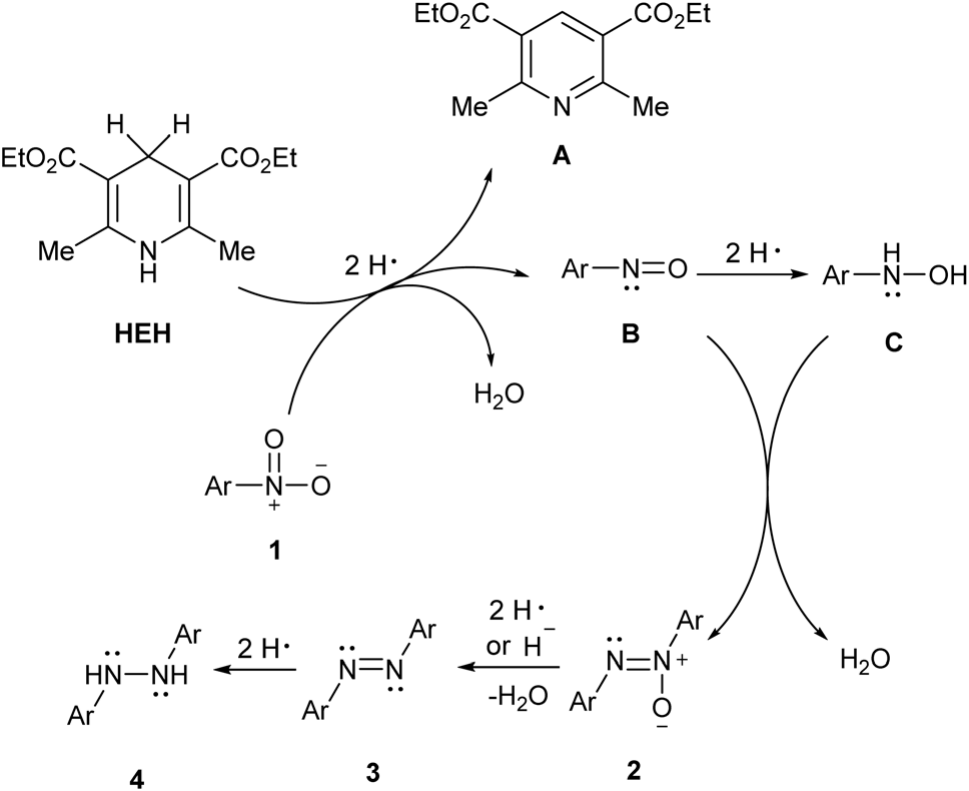
Proposed reaction mechanism.

Based on the above experiments and the free radical reaction system involving dihydropyridine,^45,46^ a plausible mechanism for the hydrogenative coupling of nitroarenes in the HEH reduction system was proposed (Scheme 5).

HEH was thermally activated by a strong base to produce 2 equivalents of hydrogen radicals and pyridine A. The nitroso species B was obtained by the addition of hydrogen radical to the N=O bond in nitro group in substrate 1. With further treatment of 2H˙, hydroxylamine C was produced followed by a nucleophilic addition of N-atom to N=O in nitroso group in C to provide azoxy 2. Deoxygenation of 2 under the synergistic effect of the two hydrogen radicals and the hydrogen anion of the sodium hydride resulted in the generation of azobenzene 3. The N=N double bond in 3 underwent hydrogenative reduction to produce diarylhydrazine 4. The addition of 4 Å molecular sieves to the standard reaction mixture was used to evaluate the role of the dehydration step in the mechanism of nitrotoluene conversion. However, no influence on the reaction was detected. It was worth noting that 1-bromo-3-nitrobenzene was reduced beyond the azoxy stage, affording only azoarene 3g. The influence of the drying agent would depend on the characteristics of the substrates.

### 2.4 Formation of phenazines

During the optimization of the synthesis conditions of aromatic azo derivatives, it was accidentally found that nitroarenes with an F atom connecting to the adjacent position of nitro could be constructed into a fused aromatic phenazine rather than an azo compound (Table 4).

**Table 4:** Reduction of 2-nitrofluoroarenes for the synthesis of phenazines 9a–g

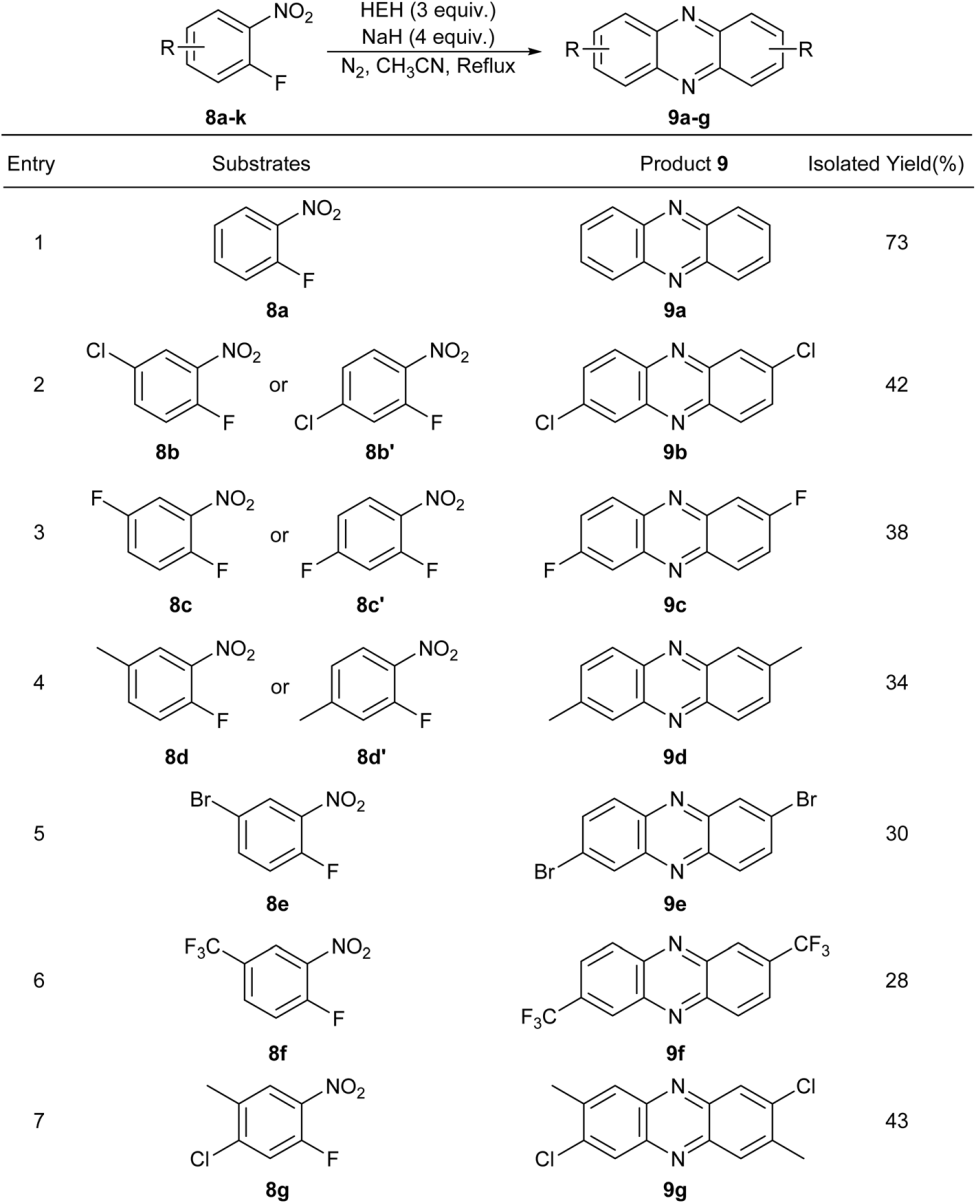

As heterocycles with diverse bioactivities in natural products,^47–49^ early synthetic approaches for phenazines relied on simple substrates but required harsh conditions involving elevated temperatures, stoichiometric toxic oxidants, expensive ligands, and transition metal catalysts. Recent studies have explored *ortho*-halogenated nitrogenous aromatics as substrates, yet these methods still depend on intricate reducing systems.^50^

According to the results in Table 4, phenazine compounds could be synthesized from *o*-fluoronitroarene, requiring 3 equivalents of HEH and 4 equivalents of sodium hydride. The substrates with electron donating and electron withdrawing groups can react with each other and offer a wide range of substrate compatibility (Table 4, 9a–g). Therefore, a new method for the construction of phenazine framework was established.

## 3 Conclusions

In summary, an HEH-based reductant, a transition metal-free system, was developed for the chemoselective hydrogenative coupling of nitroarenes. The diversity of the products, namely, aromatic azoxy, azo, hydrazine and phenazine, indicated that the reactivity of the reductant could be controlled, demonstrating the potential for expanded applications. Broad substrate compatibility was observed, especially with some groups that are sensitive to transition metallic processes such as halogen, cyano and carbonyl. Construction of phenazine derivatives provided a new method for the synthesis of such N-containing fused heterocyclic frameworks. Additionally, the formation of a C–N bond could stimulate the expansion of the studies on the chemistry of carbon–heteroatom bonds in arenes in transition metal-free reactions. Furthermore, the radical mechanism of HEH-based reductant would create new potentials for the cleavage and formation of inert carbon–heteroatom bonds.

## Author contributions

Chuang Lu: writing – original draft, investigation, data curation. Dejun Zhou: data curation, editing. Yangqi Zhang: data curation. Siye Du: data curation. Qiaomei Zheng: data curation. Di Wu: data curation. Weixin Zheng: supervision, writing – review & editing, funding acquisition.

## Conflicts of interest

The authors declare that there are no conflicts of interest.

## Supplementary Material

RA-015-D5RA04782J-s001

## Data Availability

The data that support the findings of this study, including the procedures and copies of NMR spectra, are openly available in the SI as a separate file (pdf). Supplementary information: Experimental procedures, ^1^H and ^13^C NMR spectra of synthesized compound. See DOI: https://doi.org/10.1039/d5ra04782j.
