# Supplementary material for: Transition metal-free hydrogenative coupling of nitroarenes mediated with dihydropyridine: chemoselective formation of aromatic azoxy, azo, hydrazine and phenazine
Source: RSC Adv. 2025 Sep 15;15(40):33506–14. doi: 10.1039/d5ra04782j (PMC12434464; doi:10.1039/d5ra04782j)
Supplement: RA-015-D5RA04782J-s001 [file RA-015-D5RA04782J-s001.pdf]

Supplementary Information (SI) for RSC Advances.
This journal is © The Royal Society of Chemistry 2025

Transition Metal-free Hydrogenative Coupling of Nitroarenes Mediated with Dihydropyridine: Chemoselective Formation of Aromatic azoxy, azo, Hydrazine and Phenazine

Chuang Lu, Dejun Zhou, Yangqi Zhang, Siye Du, Qiaomei Zheng, Di Wu and Weixin Zheng*
College of Material, Chemistry & Chemical Engineering Hangzhou Normal University Hangzhou 311121, China

Supporting Information

Table of Contents

1 General 2
2 Procedures and Characterization Data 2
- 2.1 General procedures for the syntheses of aromatic azoxy **2a-j**, azo **3a-k**, hydrazine **4a-d** .. 2
- 2.2 Synthesis of pyrazolidin-3-one **5a-d** 7
- 2.3 Control experiments for mechanism 8
- 2.4 Synthesis of phenazine derivatives **9a-g** 9

3 Copies of ^{1}H and ^{13}C NMR Spectra 11
4 Copies of ^{19}F NMR Spectra of **2b**, **3b**, **9c** and **9f** 44

1 General

Unless otherwise noted, all starting materials were commercially available and were used without further purification. Nitrosyltoluene **6** was prepared according to the reported method.[1] ^{1}H and ^{13}C NMR spectra were recorded on Bruker Avance DMX500 in $CDCl_3$ solutions and with tetramethyl silane as internal standard. High resolution electrospray ionization mass spectra were recorded on Agilent 6500 Q-TOF. All the spectra of products can be found in Supplementary Materials. Melting points were recorded on Micro melting point meter X5. R_f values were calculated by dividing the distance traveled by the compound by the distance traveled by the solvent front.

2 Procedures and Characterization Data

2.1 General procedures for the syntheses of aromatic azoxy 2a-j, azo 3a-k, hydrazine 4a-d

A solution of nitroarene (1.0 mmol) in acetonitrile (5 mL) was sequentially treated with HEH (3.0 mmol) and NaOH (3.0 mmol) for azoxy **2a-j** (Table 1). In case of azo **3a-k**, 2.2 mmol of HEH and NaH (5.0 mmol) were involved (Table 3). Both 4.0 mmol of HEH and NaH were used for the formation of hydrazine **4a-d** (Table 5). The reaction mixture was refluxed with continuous stirring until thin-layer chromatography (TLC) analysis indicated complete consumption of the starting material. After cooling to room temperature, the mixture was diluted with deionized water (10 mL) and the aqueous phase was extracted with ethyl acetate (3 × 15 mL). The combined organic extracts were washed with 10 mL of saturated brine, dried over anhydrous Na_2SO_4, and concentrated under reduced pressure. The crude product was purified by flash column chromatography on neutral alumina using a gradient eluent system of ethyl acetate/petroleum ether to afford compounds **2a**-**2j**, **3a-k** and **4a-d**.

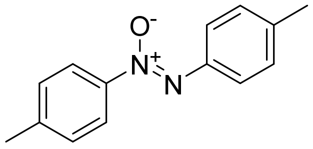

1,2-Di-*p*-tolyldiazene-1-oxide **(2a)**. Pale yellow solid (0.201 g, 89%); R_f = 0.7 (petroleum : ethyl acetate = 15:1); m.p. 60-62 °C; ^{1}H NMR (500 MHz, $CDCl_3$) δ (ppm): 8.16 (d, J = 8.5 Hz, 2H), 8.11 (d, J = 8.5 Hz, 2H), 7.26-7.24 (m, 2H), 2.40 (s, 3H), 2.38 (s, 3H); ^{13}C{^{1}H} NMR (126 MHz, $CDCl_3$) δ (ppm): 146.3, 142.0, 140.0, 129.7, 129.3, 125.7, 122.8, 122.2, 21.6, 21.3. The NMR data were consistent with reported data.[2]

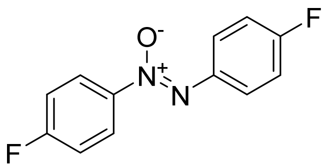

1,2-Bis(4-fluorophenyl)diazene oxide **(2b)**. Pale yellow solid (0.150 g, 64%); $R_f = 0.6$ (petroleum : ethyl acetate = 15:1); m.p. 90-92 °C; ^{1}H NMR (500 MHz, $CDCl_3$) δ (ppm): 8.32-8.29 (m, 2H), 8.26-8.23 (m, 2H), 7.18-7.13 (m, 4H); $^{13}C\{^1H\}$ NMR (126 MHz, $CDCl_3$) δ (ppm): 164.5 (d, J = 253.3 Hz), 162.5 (d, J = 253.0 Hz), 144.3, 140.3 (d, J = 3.2 Hz), 128.0 (d, J = 8.5 Hz), 124.5 (d, J = 9.2 Hz), 115.8 (d, J = 6.7 Hz), 115.6 (d, J = 5.7 Hz); $^{19}F\{^1H\}$ NMR (470 MHz, $CDCl_3$) δ (ppm): -108.05, -108.61. The ^{1}H and ^{13}C NMR data were consistent with reported data.[2]

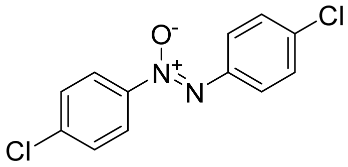

(Z)-1,2-bis(4-chlorophenyl)diazene 1-oxide **(2c)**. Pale yellow solid (0.198 g, 74%); $R_f = 0.7$ (petroleum : ethyl acetate = 15:1); m.p. 154-156 °C; ^{1}H NMR (500 MHz, $CDCl_3$) δ (ppm): 8.25 (d, J = 8.9 Hz, 2H), 8.15 (d, J = 8.9 Hz, 2H), 7.47 (d, J = 8.9 Hz, 2H), 7.44 (d, J = 8.9 Hz, 2H); $^{13}C\{^1H\}$ NMR (126 MHz, $CDCl_3$) δ (ppm): 146.6, 142.2, 138.1, 135.3, 129.0, 129.0, 127.1, 123.7. The NMR data were consistent with reported data.[2]

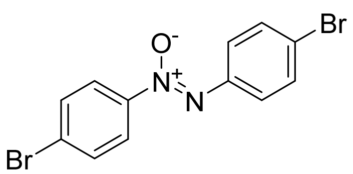

(Z)-1,2-bis(4-bromophenyl)diazene 1-oxide **(2d)**. Yellow needles (0.217 g, 61%); $R_f = 0.7$ (petroleum : ethyl acetate = 15:1); m.p. 170-172 °C; ^{1}H NMR (500 MHz, $CDCl_3$) δ (ppm): 8.17 (d, J = 9.0 Hz, 2H), 8.07 (d, J = 9.0 Hz, 2H), 7.64 (d, J = 9.0 Hz, 2H), 7.60 (d, J = 9.0 Hz, 2H); $^{13}C\{^1H\}$ NMR (126 MHz, $CDCl_3$) δ (ppm): 147.1, 142.6, 132.1, 132.0, 127.2, 126.5, 124.0, 123.6. The NMR data were consistent with reported data.[2]

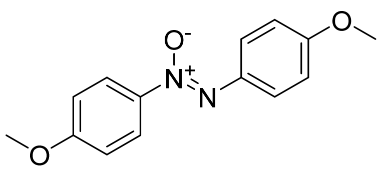

(Z)-1,2-bis(4-methoxyphenyl)diazene 1-oxide **(2e)**. Yellowish solid (0.116 g, 45%); $R_f = 0.7$ (petroleum : ethyl acetate = 5:1); m.p. 118-119 °C; ^{1}H NMR (500 MHz, $CDCl_3$) δ (ppm): 8.29-8.23 (m, 4H), 6.98-6.94 (m, 4H), 3.87 (s, 3H), 3.86 (s, 3H); $^{13}C\{^1H\}$ NMR (126 MHz, $CDCl_3$) δ (ppm): 161.9, 160.2, 141.7, 138.0, 127.8, 123.8, 113.8, 113.6, 55.6, 55.5. The NMR data were consistent with reported data.[3]

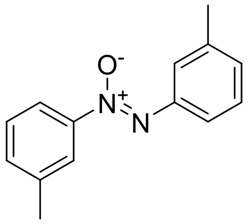

(Z)-1,2-bis(4-methoxyphenyl)diazene 1-oxide **(2f)**. Orange oil (0.190 g, 84%); $R_f = 0.6$ (petroleum : ethyl acetate = 15:1); ^{1}H NMR (500 MHz, $CDCl_3$) δ (ppm): 8.10-8.07 (m, 2H), 7.98-7.96 (m, 2H), 7.37-7.32 (m, 3H), 7.19 (d, J = 7.6 Hz, 1H), 2.47 (s, 3H), 2.43 (s, 3H); $^{13}C\{^1H\}$ NMR (126 MHz, $CDCl_3$) δ (ppm): 148.4, 144.1, 139.0, 138.5, 132.3, 130.4, 128.6, 128.5, 126.1, 122.8, 122.6,

119.5, 21.5, 21.4. The NMR data were consistent with reported data.[2]

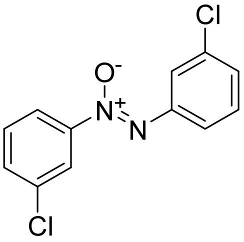

(Z)-1,2-bis(3-chlorophenyl)diazene 1-oxide **(2g)**. Pale yellow solid (0.123 g, 46%); R_f = 0.7 (petroleum : ethyl acetate = 15:1); m.p. 97-98 °C; ^{1}H NMR (500 MHz, $CDCl_3$) δ (ppm): 8.29 (t, J = 2.1 Hz, 1H), 8.24 (t, J = 1.8 Hz, 1H), 8.17 (ddd, J = 8.3, 2.1, 1.0 Hz, 1H), 7.98 (dt, J = 7.7, 1.6 Hz, 1H), 7.52 (ddd, J = 8.0, 2.0, 1.0 Hz, 1H), 7.45-7.35 (m, 3H); ^{13}C{^{1}H} NMR (126 MHz, $CDCl_3$) δ (ppm): 148.8, 144.5, 134.8, 134.4, 132.0, 129.9.0, 129.8, 129.7, 125.4, 124.1, 122.8, 120.6. The NMR data were consistent with reported data.[2]

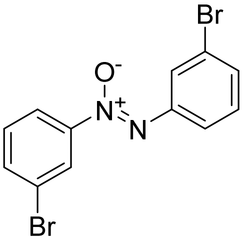

(Z)-1,2-bis(3-bromophenyl)diazene 1-oxide **(2h)**. Yellowish solid (0.107 g, 30%); m.p. 112-113 °C; R_f = 0.7 (petroleum : ethyl acetate = 15:1); ^{1}H NMR (500 MHz, $CDCl_3$) δ (ppm): 8.47 (t, J = 2.0 Hz, 1H), 8.41 (t, J = 2.0 Hz, 1H), 8.24 (ddd, J = 8.3, 2.0, 0.8 Hz, 1H), 8.05 (ddd, J = 8.1, 1.7, 0.9 Hz, 1H), 7.71 (ddd, J = 8.0, 1.8, 0.9 Hz, 1H), 7.54 (ddd, J = 8.0, 1.8, 0.9 Hz 1H), 7.40 (t, J = 8.1 Hz, 2H), 7.36 (t, J = 8.1 Hz, 2H); ^{13}C{^{1}H} NMR (126 MHz, $CDCl_3$) δ (ppm):148.9, 144.7, 135.0, 132.8, 130.2, 130.1, 128.3, 125.7, 124.5, 122.5, 122.4, 121.1. The NMR data were consistent with reported data.[2]

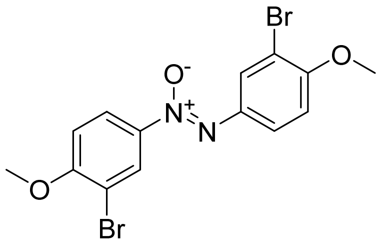

(Z)-1,2-bis(3-bromo-4-methoxyphenyl)diazene 1-oxide **(2i)**. Yellow needles (0.325 g, 78%); R_f = 0.6 (petroleum : ethyl acetate = 3:1); m.p. 192-193°C; ^{1}H NMR (500 MHz, $CDCl_3$) δ (ppm): 8.58 (d, J = 3.0 Hz, 1H), 8.50 (d, J = 3.4 Hz, 1H), 8.24 (dd, J = 11.4, 3.0 Hz, 1H) 8.20 (dd, J = 11.4, 3.4 Hz, 1H), 6.95 (d, J = 10.9 Hz, 1H), 6.93 (d, J = 10.6 Hz, 1H), 3.97 (s, 3H), 3.96 (s, 3H); ^{13}C{^{1}H} NMR (126 MHz, $CDCl_3$) δ (ppm): 158.4, 156.6, 141.5, 138.1, 131.2, 127.4, 126.9, 122.7, 111.3, 113.2, 113.0, 110.7, 56.7, 56.5; HRMS (ESI) calcd for $C_{14}H_{12}Br_2N_2N_2NaO_3$ $[M+Na]^+$: 436.9112, found 436.9116.

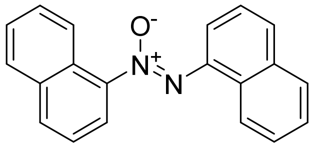

(Z)-1,2-di(naphthalen-1-yl)diazene 1-oxide **(2j)**. Red solid (0.137 g, 46%); R_f = 0.6 (petroleum : ethyl acetate = 5:1); m.p. 124-125 °C; ^{1}H NMR (500 MHz, $CDCl_3$) δ (ppm): 9.14 (d, J = 7.7 Hz, 1H), 8.51 (d, J = 7.4 Hz, 1H), 8.35 (d, J = 8.3 Hz 1H), 8.01-7.99 (m, 2H), 7.93 (t, J = 7.5 Hz, 2H), 7.89-7.87 (m, 1H), 7.63-7.50 (m, 6H); ^{13}C{^{1}H} NMR (126 MHz, $CDCl_3$) δ (ppm): 147.3,139.4, 134.4, 134.1, 131.0, 130.4, 128.3, 128.1, 128.0, 127.0, 126.8, 126.5, 126.0, 125.5, 124.7, 123.6, 123.0, 121.5,

119.6. The NMR data were consistent with reported data.[4]

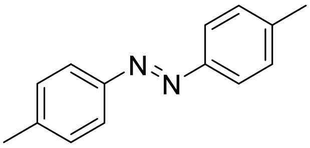

(E)-1,2-di-p-tolyldiazene **(3a)**. Yellow solid (0.158 g, 75%); m.p. 142-143 °C; R_f = 0.7 (petroleum : ethyl acetate = 15:1); ^{1}H NMR (500 MHz, $CDCl_3$) δ (ppm): 7.81 (d, J = 8.3 Hz, 4H), 7.30 (d, J = 8.1 Hz, 4H), 2.43 (s, 6H); $^{13}C\{^1H\}$ NMR (126 MHz, $CDCl_3$) δ (ppm): 150.8, 141.2, 129.7, 122.7, 21.5. The NMR data were consistent with reported data.[5]

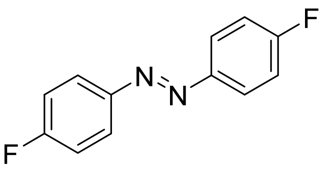

(E)-1,2-bis(4-fluorophenyl)diazene **(3b)**. Orange plates (0.205 g, 94%); R_f = 0.7 (petroleum : ethyl acetate = 15:1); m.p. 102-103 °C; ^{1}H NMR (500 MHz, $CDCl_3$) δ (ppm): 7.94-7.91 (m, 4H), 7.20 (t, J = 10.9 Hz, 4H); $^{13}C\{^1H\}$ NMR (126 MHz, $CDCl_3$) δ (ppm): 164.4 (d, J = 252.6 Hz), 149.0 (d, J = 9.5 Hz). 124.8 (d, J = 9.1 Hz), 116.0 (d, J = 22.7 Hz); $^{19}F\{^1H\}$ NMR (470 MHz, $CDCl_3$) δ (ppm): -109.24. The ^{1}H and ^{13}C NMR data were consistent with reported data.[5]

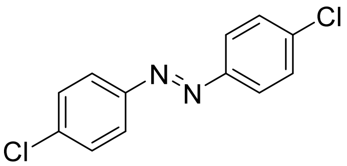

(E)-1,2-bis(4-chlorophenyl)diazene **(3c)**. Orange needles (0.236 g, 94%); R_f = 0.7 (petroleum : ethyl acetate = 15:1); m.p. 184-185 °C; ^{1}H NMR (500 MHz, $CDCl_3$) δ (ppm): 7.86 (d, J = 8.7 Hz, 4H), 7.49 (d, J = 8.7 Hz, 4H); $^{13}C\{^1H\}$ NMR (126 MHz, $CDCl_3$) δ (ppm): 150.8, 137.2, 129.4, 124.2. The NMR data were consistent with reported data.[5]

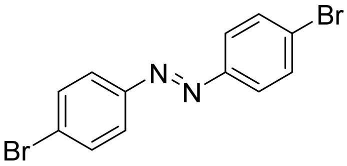

(E)-1,2-bis(4-bromophenyl)diazene **(3d)**. Orange powder (0.214 g, 63%); R_f = 0.8 (petroleum : ethyl acetate = 15:1); m.p. 204-205 °C; ^{1}H NMR (500 MHz, $CDCl_3$) δ (ppm): 7.79 (d, J = 8.8 Hz, 4H), 7.65 (d, J = 8.8 Hz, 4H); $^{13}C\{^1H\}$ NMR (126 MHz, $CDCl_3$) δ (ppm): 151.2, 132.4, 125.8, 124.4. The NMR data were consistent with reported data.[5]

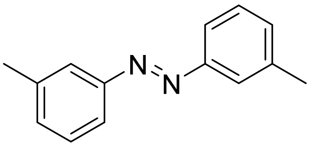

(E)-1,2-di-m-tolyldiazene **(3e)**. Orange oil (0.174 g, 83%); R_f = 0.7 (petroleum : ethyl acetate = 15:1); ^{1}H NMR (500 MHz, $CDCl_3$) δ (ppm): 7.72-7.71 (m, 4H), 7.39-7.36 (m, 2H), 7.26 (d, J = 7.5 Hz, 2H), 2.43 (s, 6H); $^{13}C\{^1H\}$ NMR (126 MHz, $CDCl_3$) δ (ppm): 152.8, 138.9, 131.6, 128.8, 122.9, 120.4, 21.3. The NMR data were consistent with reported data.[5]

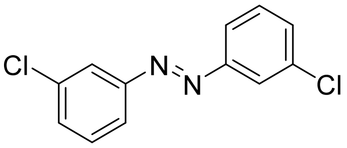

(E)-1,2-bis(3-chlorophenyl)diazene **(3f)**. Orange plates (0.239 g, 95%); R_f = 0.7 (petroleum : ethyl acetate = 15:1); m.p. 100-101 °C; ^{1}H NMR (500 MHz, $CDCl_3$) δ (ppm): 7.88-7.87 (m, 2H), 7.82-

7.80 (m, 2H), δ 7.45-7.44 (m, 4H); $^{13}C\{^1H\}$ NMR (126 MHz, $CDCl_3$) *δ* (ppm): 153.1, 135.2, 131.2, 130.1, 122.6, 121.9. The NMR data were consistent with reported data.[6]

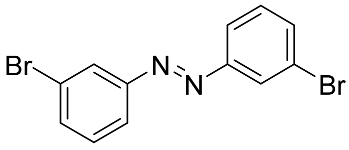

(E)-1,2-bis(3-bromophenyl)diazene **(3g)**. Orange plates (0.224 g, 66%); R_f = 0.7 (petroleum : ethyl acetate = 15:1); m.p. 150-151 °C; ^1H NMR (500 MHz, $CDCl_3$) *δ* (ppm): 8.03 (t, *J* = 1.9 Hz, 2H), 7.86 (ddd, *J* = 8.0, 1.8,1.1 Hz, 2H), 7.59 (ddd, *J* = 8.0, 1.9, 1.0 Hz, 2H), 7.38 (t, *J* = 7.9 Hz, 2H); $^{13}C\{^1H\}$ NMR (126 MHz, $CDCl_3$) *δ* (ppm): 153.2, 134.1, 130.5, 124.7, 123.2. The NMR data were consistent with reported data.[6]

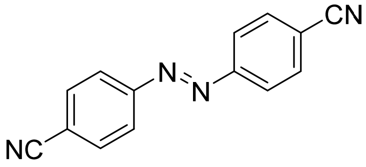

(E)-4,4'-(diazene-1,2-diyl)dibenzonitrile **(3h)**. Red solid (0.177 g, 76%); R_f = 0.3 (petroleum : ethyl acetate = 3:1); m.p. 233-234 °C; ^1H NMR (500 MHz, $CDCl_3$) *δ* (ppm): 8.04 (d, *J* = 10.6 Hz, 4H), δ 7.86 (d, *J* = 10.6 Hz, 4H); $^{13}C\{^1H\}$ NMR (126 MHz, $CDCl_3$) *δ* (ppm): 154.0, 133.3, 123.7, 118.1, 115.1. The NMR data were consistent with reported data.[5]

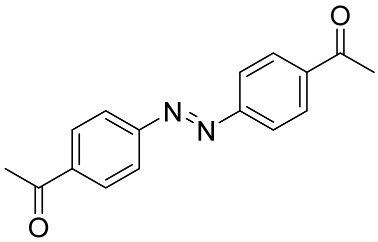

(E)-1,1'-(diazene-1,2-diylbis(4,1-phenylene))bis(ethan-1-one) **(3i)**. Pale red solid (0.200 g, 75%) R_f = 0.7 (petroleum : ethyl acetate = 3:1);; m.p. 192-193 °C; ^1H NMR (500 MHz, $CDCl_3$) *δ* (ppm): 8.12 (d, *J* = 8.6 Hz, 4H), δ 8.01 (d, *J* = 8.6 Hz, 4H), 2.68 (s, 6H); $^{13}C\{^1H\}$ NMR (126 MHz, $CDCl_3$) *δ* (ppm): 197.4, 154.8, 138.9, 129.4, 123.2, 26.9. The NMR data were consistent with reported data.[7]

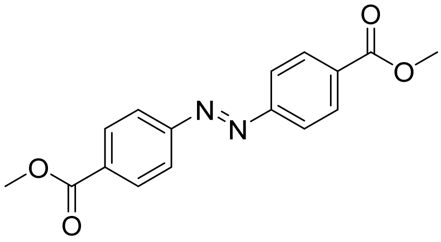

(E)-dimethyl 4,4'-(diazene-1,2-diyl) dibenzoate **(3j)**. Orange powder (0.185 g, 62%); R_f = 0.7 (petroleum : ethyl acetate = 2:1); mp 196-197 °C; ^1H NMR (500 MHz, $CDCl_3$) *δ* (ppm): 8.21 (d, *J* = 8.6 Hz, 4H), δ 7.99 (d, *J* = 8.6 Hz, 4H), 3.97 (s, 6H); $^{13}C\{^1H\}$ NMR (126 MHz, $CDCl_3$) *δ* (ppm): 166.4, 154.9, 132.4, 130.7, 122.9, 52.4. The NMR data were consistent with reported data.[8]

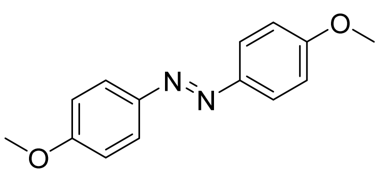

(E)-1,2-bis(4-methoxyphenyl)diazene **(3k)**. Orange prisms (0.015 g, 6%); mp 160-161 °C; R_f = 0.7 (petroleum : ethyl acetate = 5:1); ^1H NMR (500 MHz, $CDCl_3$) *δ* (ppm): 7.88 (d, *J* = 11.2 Hz, 4H), 7.00 (d, *J* = 11.2 Hz, 4H), 3.89 (s, 6H); $^{13}C\{^1H\}$ NMR (126 MHz, $CDCl_3$) *δ* (ppm): 161.5, 147.0, 124.3, 114.1, 55.5. The NMR data were consistent with reported data.[5]

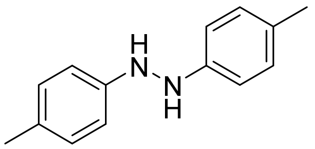

1,2-Di-p-tolylhydrazine **(4a)**. Yellow solid (90%, NMR yield); R_f = 0.4 (petroleum : ethyl acetate = 15:1); ^{1}H NMR (500 MHz, $CDCl_3$) δ (ppm): 7.01 (d, J = 8.2 Hz, 4H), 6.76 (d, J = 8.2 Hz, 4H), 5.51 (s, 2H), 2.26 (s, 6H). The NMR data were consistent with reported data.[9]

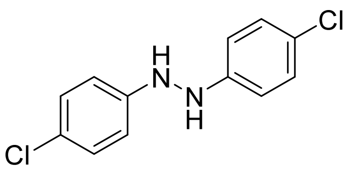

1,2-Bis(4-chlorophenyl)hydrazine **(4b)**. Pale yellow solid (82%, NMR yield); R_f = 0.3 (petroleum : ethyl acetate = 15:1); ^{1}H NMR (500 MHz, $CDCl_3$) δ (ppm): 7.15 (d, J = 8.2 Hz, 4H), 6.75 (d, J = 8.2 Hz, 4H), 5.61 (s, 2H). The NMR data were consistent with reported data.[9]

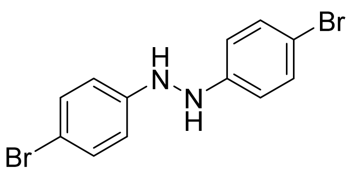

1,2-Bis(4-bromophenyl)hydrazine **(4c)**. Yellow solid (62%, NMR yield); R_f = 0.3 (petroleum : ethyl acetate = 15:1); ^{1}H NMR (500 MHz, $CDCl_3$) δ (ppm): 7.93-7.85 (m, 4H), 7.53-7.42 (m, 4H), 7.00 (d, J = 8.9 Hz, 4H), 4.11 (q, J = 7.0 Hz, 2H), 1.45 (t, J = 7.0 Hz, 3H). The NMR data were consistent with reported data.[9]

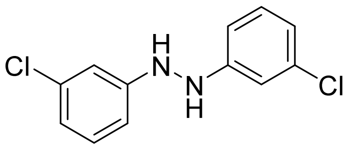

1,2-Bis(3-chlorophenyl)hydrazine **(4d)**. White powder (80%, NMR yield); R_f = 0.3 (petroleum:ethyl acetate = 15:1); ^{1}H NMR (500 MHz, $CDCl_3$) δ (ppm): 7.15 (t, J = 7.9 Hz, 2H), 6.82 (d, J = 8.7 Hz, 4H), 6.70 (d, J = 8.1 Hz, 2H), 5.65 (s, 2H). The NMR data were consistent with reported data.[9]

2.2 Synthesis of pyrazolidin-3-one 5a-d

After the standard reaction for the synthesis of hydrazine **4** in 4.1 was completed (monitored by TLC), 3-Chloropropionyl chloride (5.0 mmol) was added to trap the hydrazine 4 via in situ condensation. The mixture was stirred for further 1 h and cooled to room temperature. To the mixture was added deionized water (10 mL), and the aqueous phase was extracted with ethyl acetate (3 × 15 mL). The combined organic extracts were washed with 10 mL of saturated brine, dried over anhydrous Na_2SO_4, and concentrated under reduced pressure. Using a gradient elution system of ethyl acetate / petroleum ether, the crude product was purified by fast column chromatography on neutral alumina to obtain compounds **5a-d**.

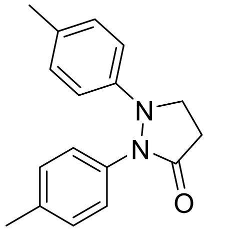

1,2-Di-p-tolylpyrazolidin-3-one **(5a)**. Yellow oil (0.107 g, 40%); R_f = 0.3 (petroleum : ethyl acetate = 3:1); ^{1}H NMR (500 MHz, $CDCl_3$) δ (ppm): 7.67 (d, J = 8.6 Hz, 2H), 7.08 (d, J = 8.4 Hz, 2H), 7.05 (d, J = 8.2 Hz, 2H), 6.87 (d, J = 8.9 Hz, 2H), 3.93 (t, J = 7.3 Hz, 2H), 2.68 (t, J = 7.3 Hz, 2H), 2.26 (s, 3H), 2.25 (s, 3H); ^{13}C{^{1}H} NMR (126 MHz, $CDCl_3$) δ (ppm): 171.5, 147.3, 135.7, 133.9, 133.3, 129.7, 129.3, 118.5, 118.4, 54.9, 31.5, 20.8, 20.6. The NMR data were consistent with reported data.[10]

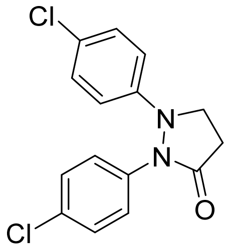

1,2-Bis(4-chlorophenyl)pyrazolidin-3-one **(5b)**. Colorless liquid (0.215 g, 70%); R_f = 0.3 (petroleum : ethyl acetate = 3:1); ^{1}H NMR (500 MHz, $CDCl_3$) δ (ppm): 7.73-7.71 (m, 2H), 7.27-7.25 (m, 2H), 7.24-7.22 (m, 2H), 6.89-6.87 (m, 2H), 3.98 (t, J = 7.3 Hz, 2H), 2.71 (t, J = 7.3 Hz, 2H).; ^{13}C{^{1}H} NMR (126 MHz, $CDCl_3$) δ (ppm): 171.5, 147.9, 136.4, 129.6, 129.3, 129.2, 129.0, 119.5, 119.5, 54.8, 31.3. The NMR data were consistent with reported data.[11]

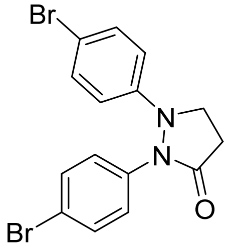

1,2-Bis(4-bromophenyl)pyrazolidin-3-one **(5c)**. White solid (0.198 g, 50%); m.p. 180-181 °C; R_f = 0.4 (petroleum : ethyl acetate = 3:1); ^{1}H NMR (500 MHz, $CDCl_3$) δ (ppm): 7.67-7.65 (m, 2H), 7.42-7.38 (m, 4H), 6.83-6.81 (m, 2H), 3.99 (t, J = 7.3 Hz, 2H), 2.72 (t, J = 7.3 Hz, 2H); ^{13}C{^{1}H} NMR (126 MHz, $CDCl_3$) δ (ppm): 171.5, 148.4, 136.9, 132.3, 132.0, 120.1, 119.8, 117.4, 116.7, 54.8, 31.4. The NMR data were consistent with reported data.[10]

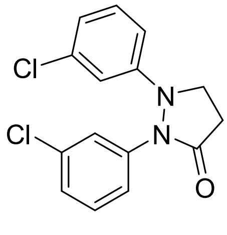

1,2-Bis(3-chlorophenyl)pyrazolidin-3-one **(5d)**. Colorless liquid (0.200 g, 65%); R_f = 0.4 (petroleum : ethyl acetate = 3:1); ^{1}H NMR (500 MHz, $CDCl_3$) δ (ppm): 7.83 (t, J = 2.1 Hz, 1H), 7.65 (ddd, J = 8.3, 2.0, 0.7 Hz, 1H), 7.23 (t, J = 8.2 Hz, 1H), 7.19 (t, J = 8.1 Hz, 1H), 7.08-7.02 (m, 2H), 6.97 (t, J = 2.2 Hz, 1H), 6.82 (ddd, J = 8.2, 2.3, 0.6 Hz, 1H), 4.00 (t, J = 7.3 Hz, 2H), 2.72 (t, J = 7.3 Hz, 2H).; ^{13}C{^{1}H} NMR (126 MHz, $CDCl_3$) δ (ppm): 171.7, 150.7, 138.9, 135.1, 134.7, 130.3, 130.0, 124.6, 124.0, 118.6, 118.1, 116.3, 116.2, 54.9, 31.4. The NMR data were consistent with reported data.[10]

2.3 Control experiments for mechanism

To the mixture of nitrotoluene (1.0 mmol), HEH (3.0 mmol) and NaH (3.0 mmol) in acetonitrile (5 mL) was added cyclohexa-1,3-diene (10.0 mmol). The above solution was refluxed for 3 hrs and monitored by TLC. After general workup, the crude product was purified over fast column chromatography on neutral alumina to obtain compounds **7**, using a gradient elution system of ethyl

acetate / petroleum ether.

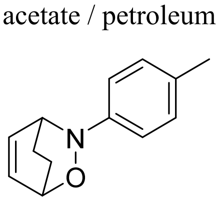

3-Tolyl-2-oxa-3-azabicyclo[2.2.2]oct-5-ene **(7)**. Brown liquid (0.006 g, 3%); ^{1}H NMR (500 MHz, $CDCl_3$) δ (ppm): 7.00 (d, J = 8.3 Hz, 2H), 6.89 (d, J = 8.3 Hz, 2H), 6.55 (t, J = 6.9 Hz, 1H), 6.11 (t, J = 6.9 Hz, 1H), 4.66-4.64 (m, 1H), 4.35-4.34 (m, 1H), 2.30-2.17 (m, 5H), 1.56-1.52 (m, 1H), 1.36-1.33 (m, 1H); ^{13}C{^{1}H} NMR (126 MHz, $CDCl_3$) δ (ppm): 149.8, 131.5, 131.2, 129.8, 128.8, 117.4, 68.9, 56.5, 23.9, 21.3, 20.5. The NMR data were consistent with reported data.[12]

2.4 Synthesis of phenazine derivatives 9a-g

To the solution of 2-fluoronitrobenzene (1.0 mmol) in anhydrous acetonitrile (5 mL) was sequentially added HEH (3.0 mmol) and NaOH (4.0 mmol). The reaction mixture was refluxed with continuous stirring until the substrate was invisible in TLC. After cooling to room temperature, the mixture was diluted with deionized water (10 mL) and the aqueous phase was extracted with ethyl acetate (3 × 15 mL). The combined organic extracts were washed with 10 mL of saturated brine, dried over anhydrous Na_2SO_4, and concentrated under reduced pressure. The crude product was purified by flash column chromatography on neutral alumina using a gradient eluent system of ethyl acetate/petroleum ether to afford compounds **9a-g**.

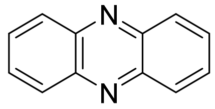

Phenazine **(9a)**. Yellow prisms (0.132 g, 73%); m.p. 176-177 °C; R_f = 0.3 (petroleum : ethyl acetate = 10:1); ^{1}H NMR (500 MHz, $CDCl_3$) δ (ppm): 8.26-8.24 (m, 4H), 7.85-7.83 (m, 4H); ^{13}C{^{1}H} NMR (126 MHz, $CDCl_3$) δ (ppm): 143.5, 130.4, 129.6. The NMR data were consistent with reported data.[12]

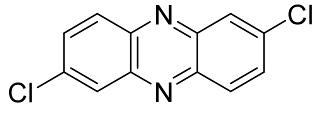

2,7-Dichlorophenazine **(9b)**. Yellow prisms (0.105 g, 42%); m.p. 265-266 °C; R_f = 0.5 (petroleum : ethyl acetate = 10:1); ^{1}H NMR (500 MHz, $CDCl_3$) δ (ppm): 8.24 (d, J = 2.2 Hz, 2H), 8.18 (d, J = 9.3 Hz, 2H), 7.79 (dd, J = 9.3, 2.3 Hz, 2H); ^{13}C{^{1}H} NMR (126 MHz, $CDCl_3$) δ (ppm): 143.3, 142.3, 136.9, 132.6, 130.9, 128.1. The NMR data were consistent with reported data.[12]

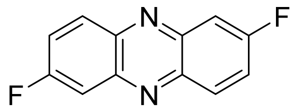

2,7-Difluorophenazine **(9c)**. Orange prisms (0.082 g, 38%); m.p. 79-80 °C; R_f = 0.5 (petroleum : ethyl acetate = 10:1); ^{1}H NMR (500 MHz, $CDCl_3$) δ (ppm): 8.24 (dd, J = 9.6, 5.5 Hz, 2H), 7.83 (dd, J = 9.2, 2.8 Hz, 2H), 7.71-7.67 (m, 2H); ^{13}C{^{1}H} NMR (126 MHz, $CDCl_3$) δ (ppm): 162.9 (d, J = 254.2 Hz), 143.3 (d, J = 15.2 Hz), 141.3, 131.7 (d, J = 11.3 Hz), 123.2 (d, J = 28.0 Hz), 111.8 (d, J = 20.2 Hz); ^{19}F NMR (470 MHz, $CDCl_3$)

δ (ppm): -105.84. The ^{1}H and ^{13}C NMR data were consistent with reported data.[13]

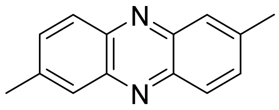

2,7-Dimethylphenazine **(9d)**. Orange prisms (0.071 g, 34%); m.p. 161-162 °C; R_f = 0.2 (petroleum : ethyl acetate = 10:1); ^{1}H NMR (500 MHz, $CDCl_3$) δ (ppm): 8.10 (d, J = 8.9 Hz, 2H), 7.97 (br, 2H), 7.65 (dd, J = 8.9, 1.9 Hz, 2H), 2.64 (s, 6H); ^{13}C{^{1}H} NMR (126 MHz, $CDCl_3$) δ (ppm): 143.2, 142.3, 140.5, 133.3, 129.0, 127.7, 22.2. The NMR data were consistent with reported data.[12]

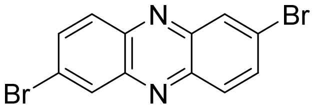

2,7-Dibromophenazine **(9e)**. Orange prisms (0.101 g, 30%); m.p. 251-252 °C; R_f = 0.5 (petroleum : ethyl acetate = 10:1); ^{1}H NMR (500 MHz, $CDCl_3$) δ (ppm): 8.43 (d, J = 2.1 Hz, 2H), 8.10 (d, J = 9.3 Hz, 2H), 7.91 (dd, J = 9.2, 2.1 Hz, 2H); ^{13}C{^{1}H} NMR (126 MHz, $CDCl_3$) δ (ppm): 143.7, 142.3, 134.9, 131.7, 130.9, 125.4. The NMR data were consistent with reported data.[13]

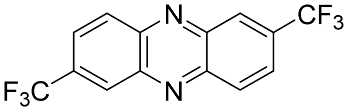

2,7-Bis(trifluoromethyl)phenazine **(9f)**. Orange prisms (0.089 g, 28%); R_f = 0.7 (petroleum : ethyl acetate = 10:1); m.p. 119-120 °C; ^{1}H NMR (500 MHz, $CDCl_3$) δ (ppm): 8.63 (s, 2H), 8.43 (d, J = 9.1 Hz 2H), 8.04 (dd, J = 9.1, 1.9, Hz, 2H); ^{13}C{^{1}H} NMR (126 MHz, $CDCl_3$) δ (ppm): 144.4, 143.1, 133.1 (q, J = 33.2 Hz), 131.5, 128.2 (q, J = 4.5 Hz), 126.5 (q, J = 2.7 Hz), 123.4 (q, J = 273.5 Hz); ^{19}F NMR (470 MHz, $CDCl_3$) δ (ppm): -63.49. The ^{1}H and ^{13}C NMR data were consistent with reported data.[5]

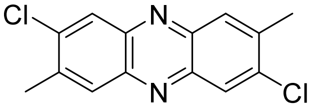

2,7-Dichloro-3,8-dimethylphenazine **(9g).** Orange prisms (0.119 g, 43%); m.p. 281-282°C; R_f = 0.7 (petroleum : ethyl acetate = 10:1); ^{1}H NMR (500 MHz, $CDCl_3$) δ (ppm): 8.23 (s, 2H), 8.06 (s, 2H), 2.67 (s, 6H); ^{13}C{^{1}H} NMR (126 MHz, $CDCl_3$) δ (ppm): 142.5, 142.2, 140.0, 138.7, 129.5, 128.1, 21.1. HRMS (ESI) calcd for $C_{14}H_{11}Cl_2N_2N_2$ $[M+H]^+$: 277.0299, found 277.0303.

3 Copies of ^{1}H and ^{13}C NMR Spectra

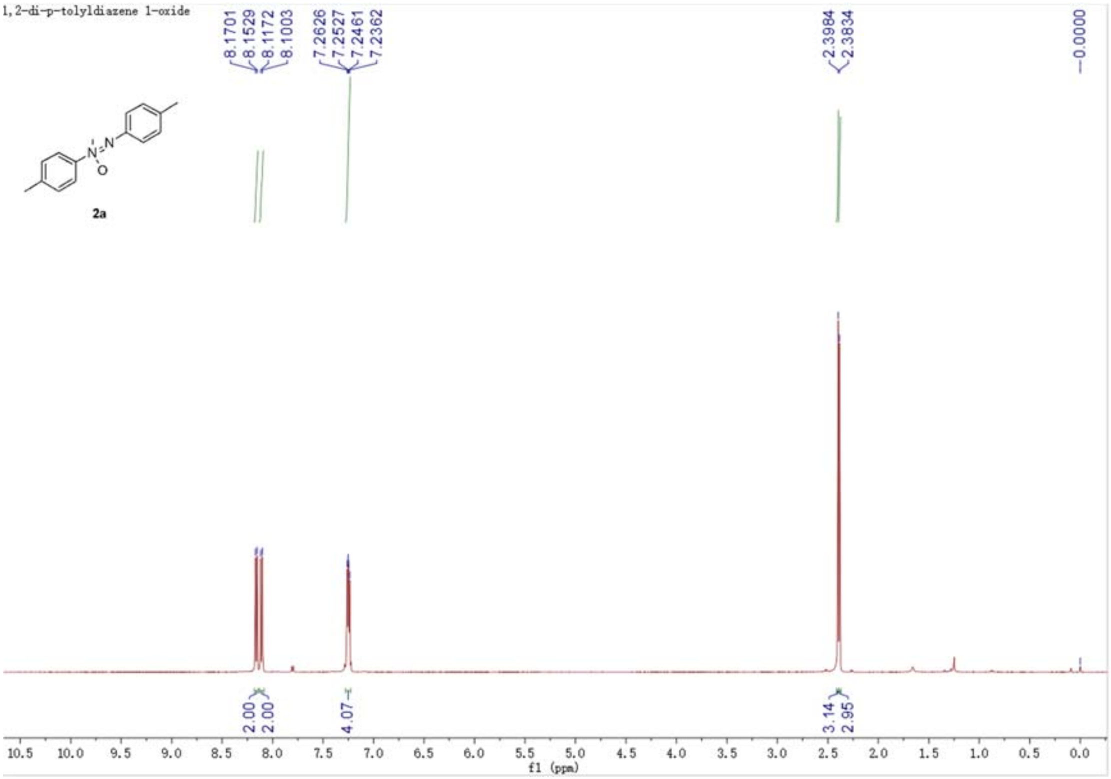

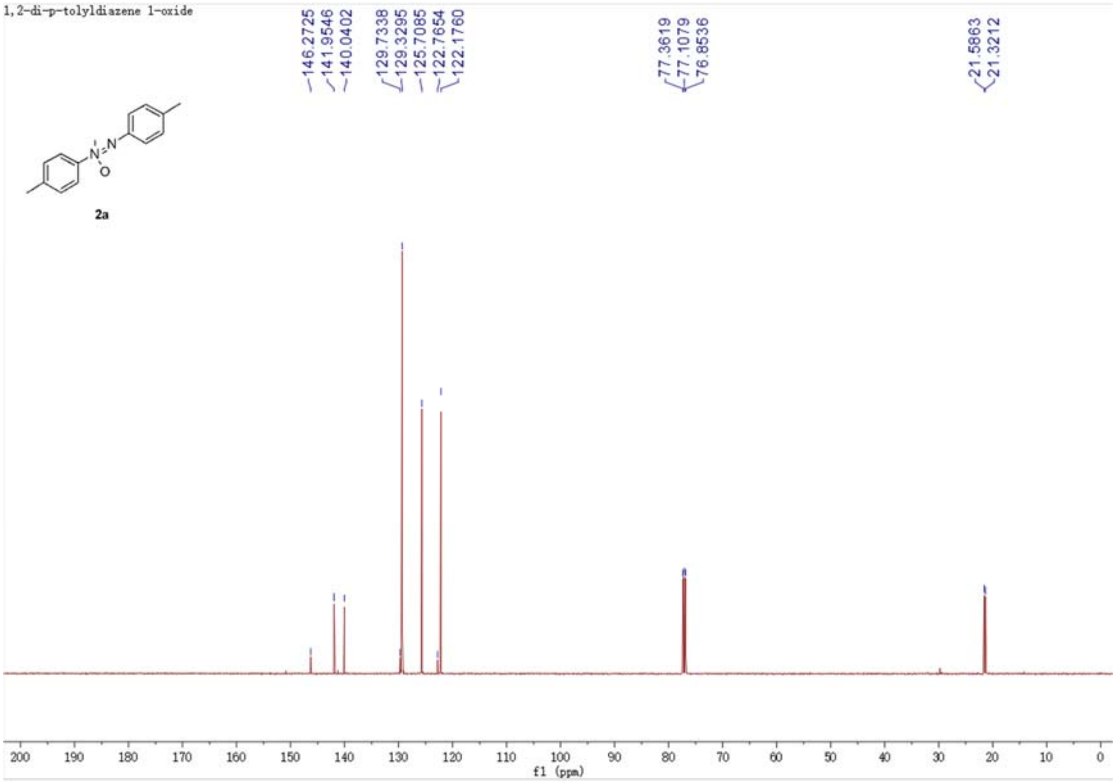

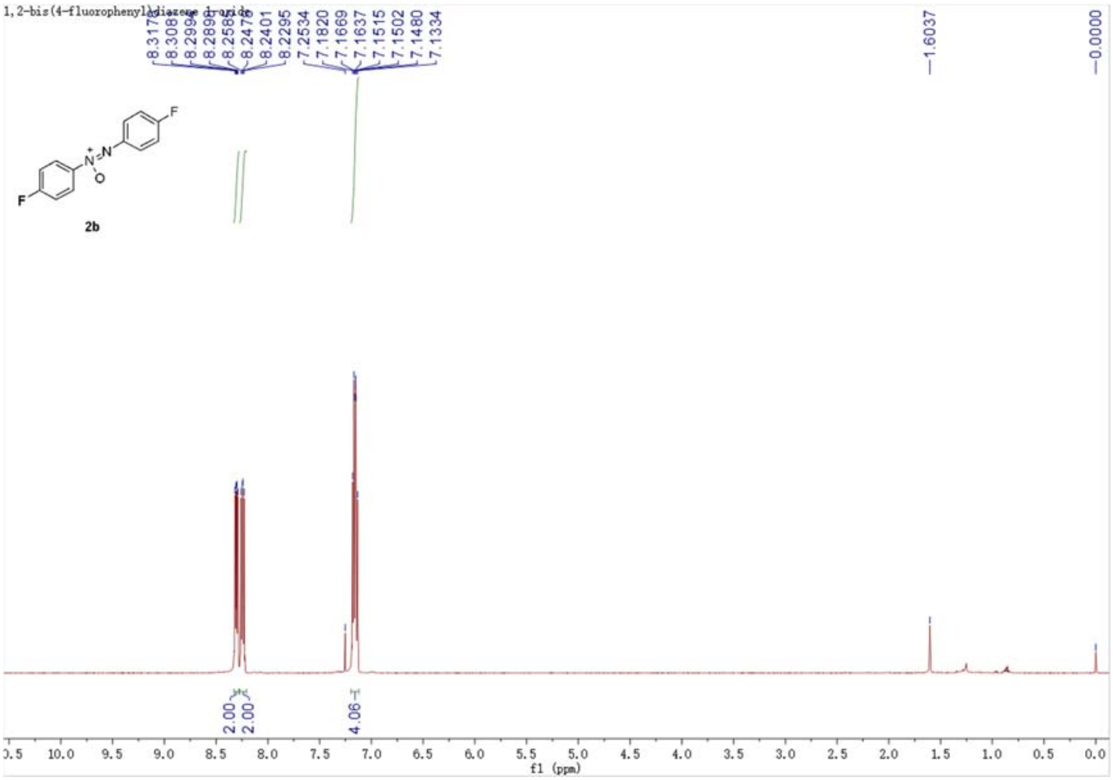
1,2-bis(4-fluorophenyl)diazene 1-oxide
8.3177
8.3083
8.2994
8.2899
8.2589
8.2477
8.2401
8.2295
7.2534
7.1820
7.1669
7.1637
7.1515
7.1502
7.1480
7.1334
1.6037
0.0000
F
N
O
2b
2.00
2.00
4.06
f1 (ppm)

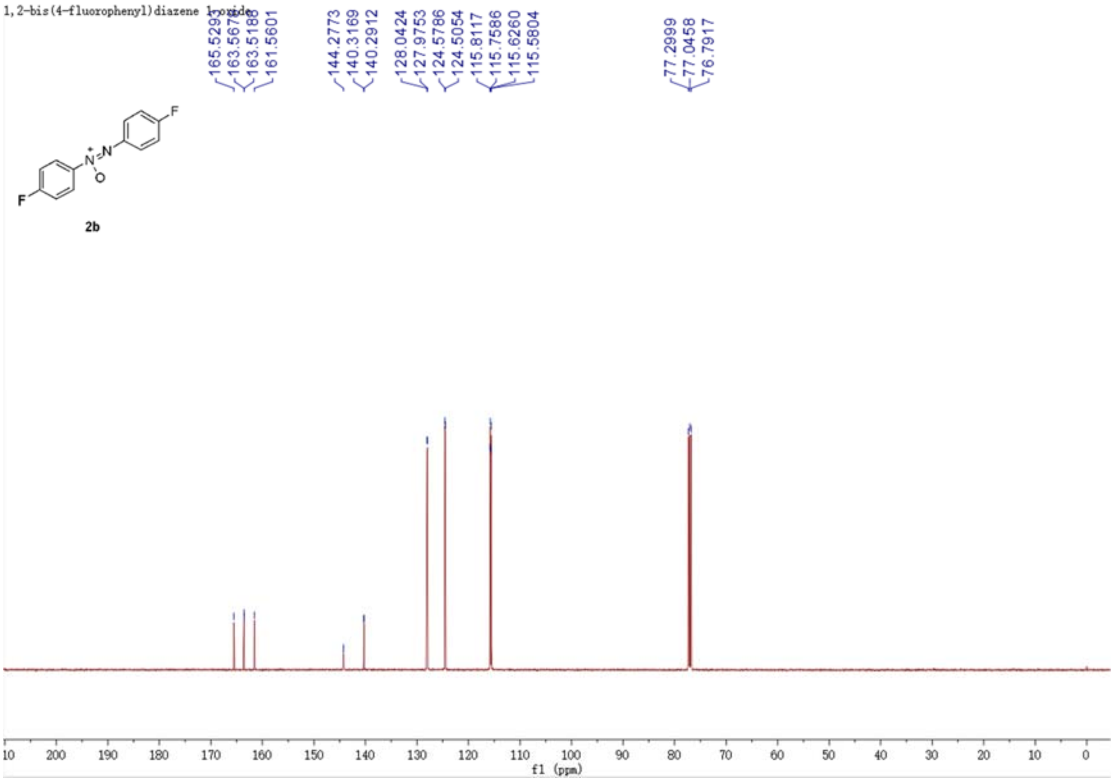
1,2-bis(4-fluorophenyl)diazene 1-oxide
165.5293
163.5678
163.5188
161.5601
144.2773
140.3169
140.2912
128.0424
127.9753
124.5786
124.5054
115.8117
115.7586
115.6260
115.5804
77.2999
77.0458
76.7917
F
N
O
2b
f1 (ppm)

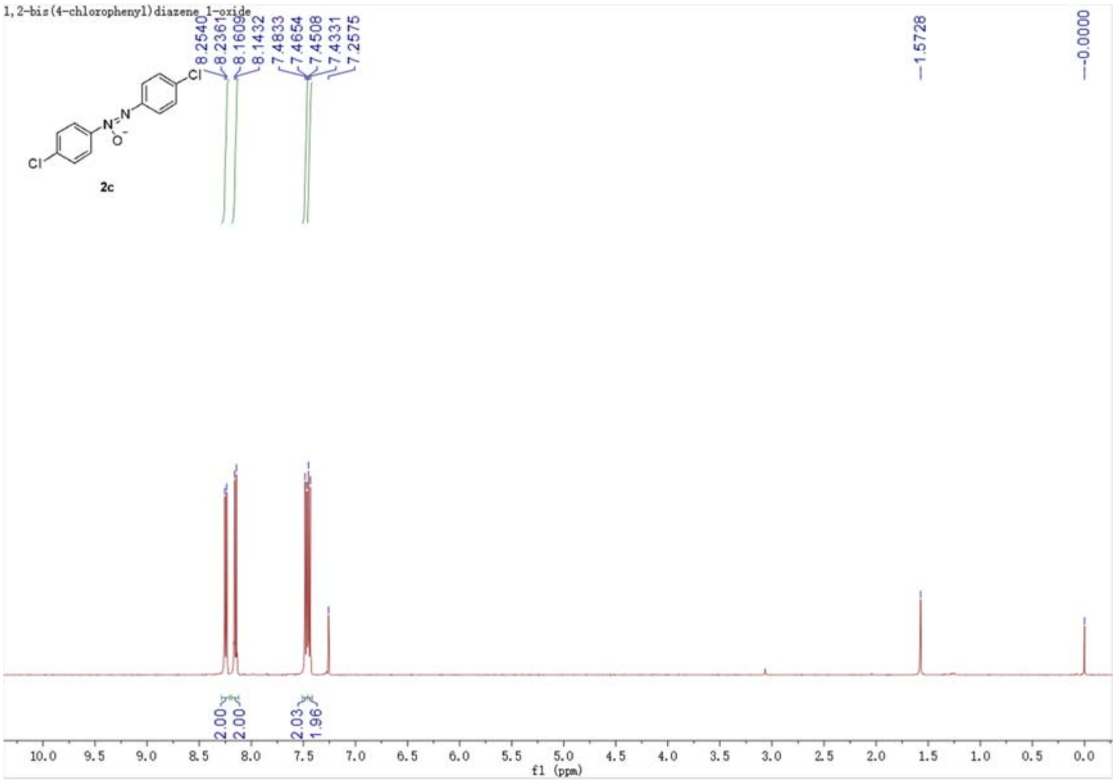
1,2-bis(4-chlorophenyl)diazene 1-oxide
8.2540
8.2361
8.1609
8.1432
7.4833
7.4654
7.4508
7.4331
7.2575
1.5728
0.0000
2c
2.00
2.00
2.03
1.96
f1 (ppm)

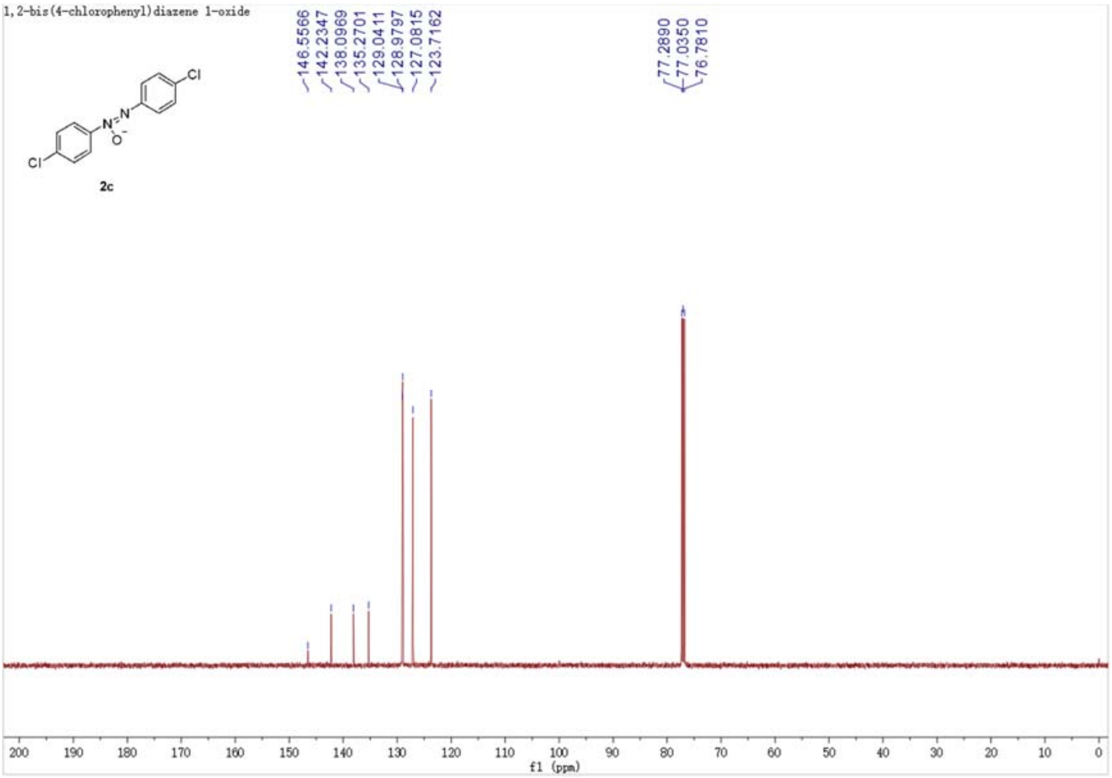
1,2-bis(4-chlorophenyl)diazene 1-oxide
146.5566
142.2347
138.0969
135.2701
129.0411
128.9797
127.0815
123.7162
77.2890
77.0350
76.7810
2c
f1 (ppm)

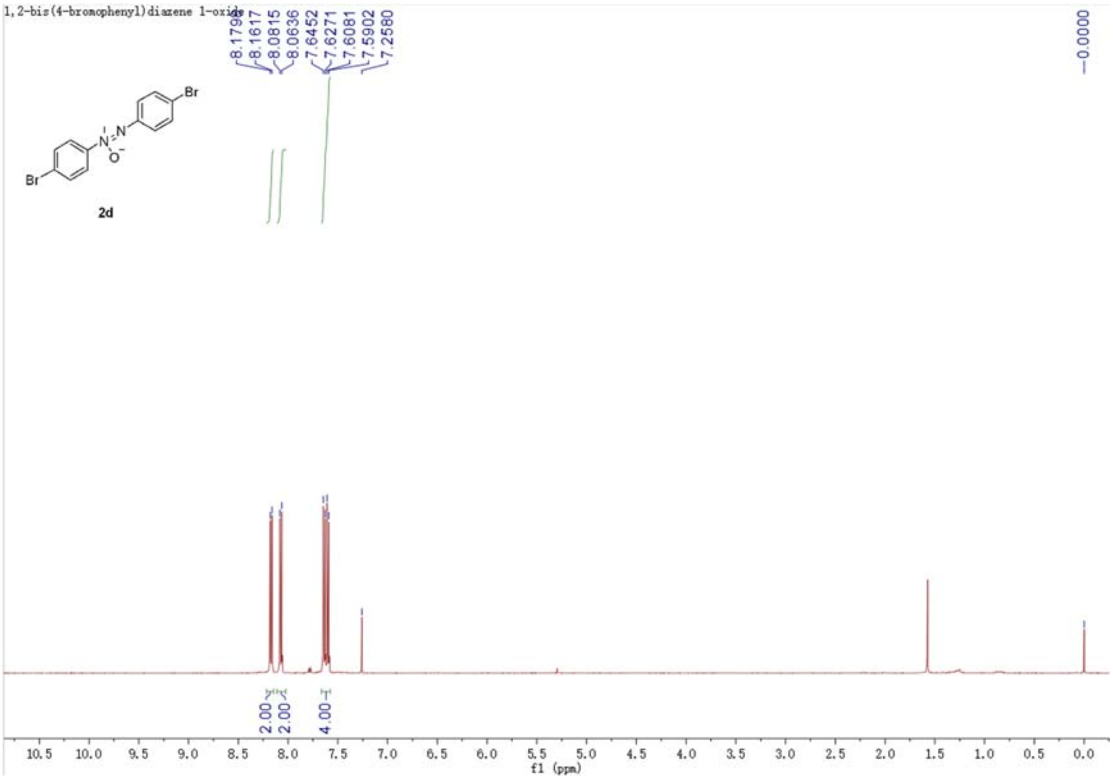
1,2-bis(4-bromophenyl)diazene 1-oxide
8.1799
8.1617
8.0815
8.0636
7.6452
7.6271
7.6081
7.5902
7.2580
0.0000
2d
2.00
2.00
4.00
f1 (ppm)

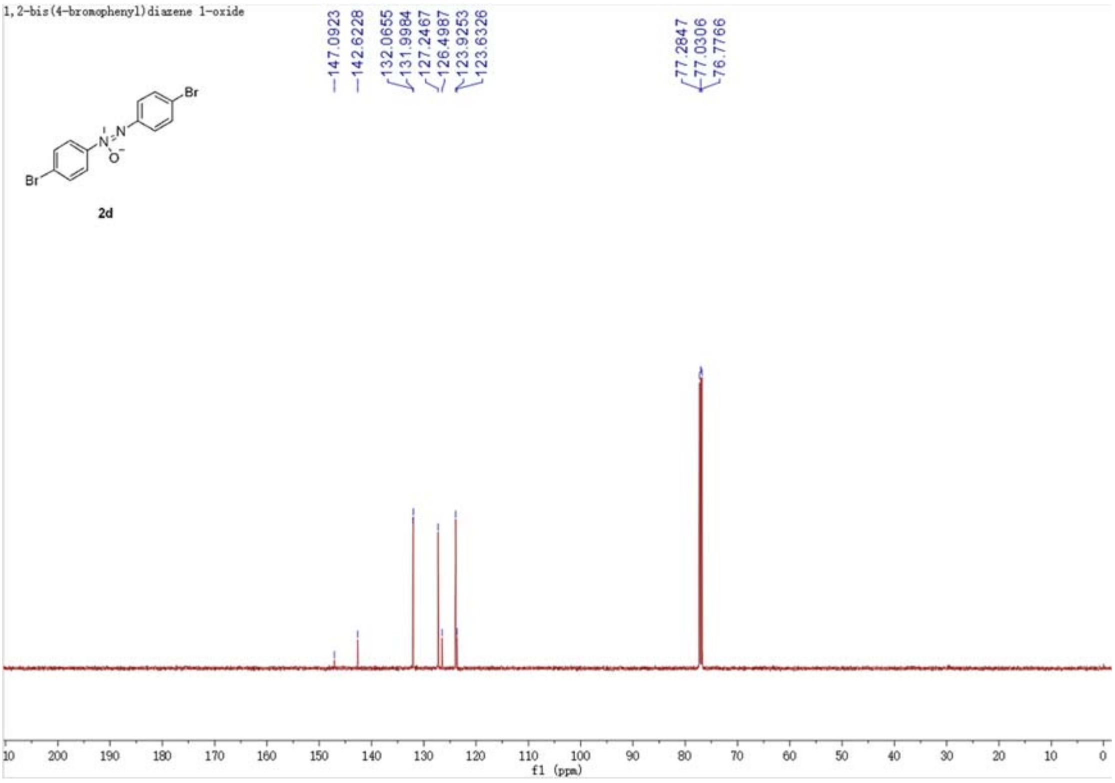
1,2-bis(4-bromophenyl)diazene 1-oxide
147.0923
142.6228
132.0655
131.9984
127.2467
126.4987
123.9253
123.6326
77.2847
77.0306
76.7766
2d
f1 (ppm)

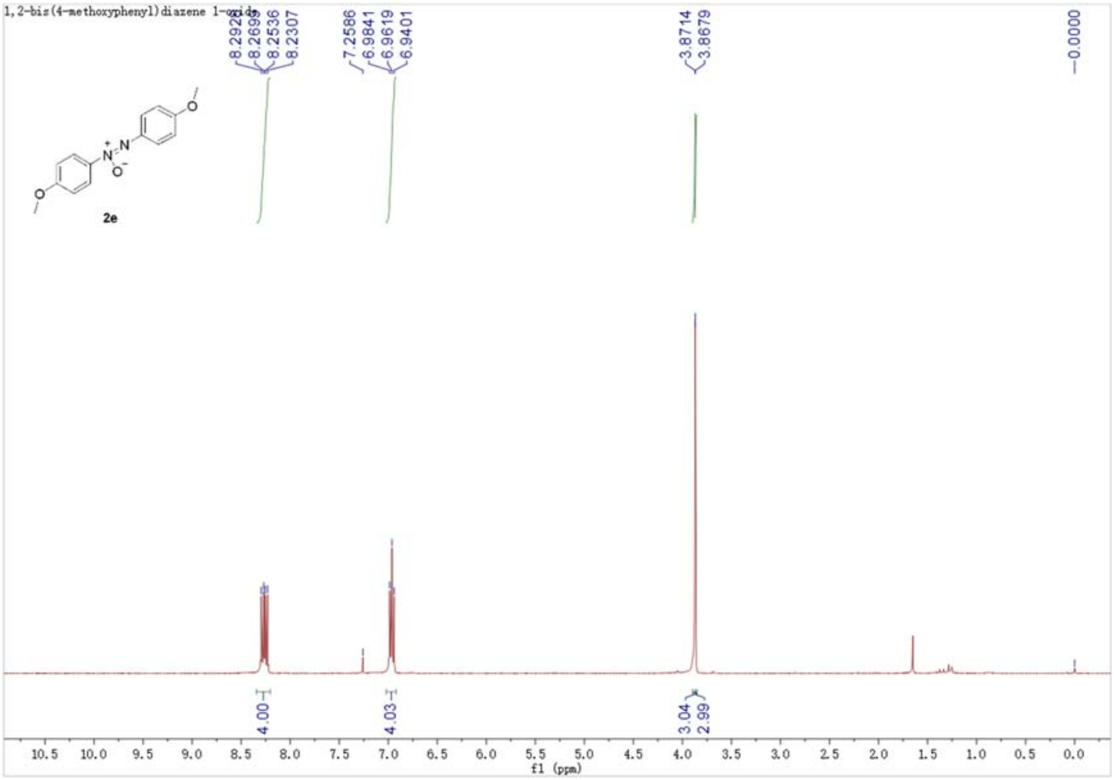
1,2-bis(4-methoxyphenyl)diazene
8.2536
8.2307
7.2586
6.9841
6.9619
6.9401
3.8714
3.8679
0.0000
2e
4.00
4.03
3.04
2.99
10.5 10.0 9.5 9.0 8.5 8.0 7.5 7.0 6.5 6.0 5.5 5.0 4.5 4.0 3.5 3.0 2.5 2.0 1.5 1.0 0.5 0.0
f1 (ppm)

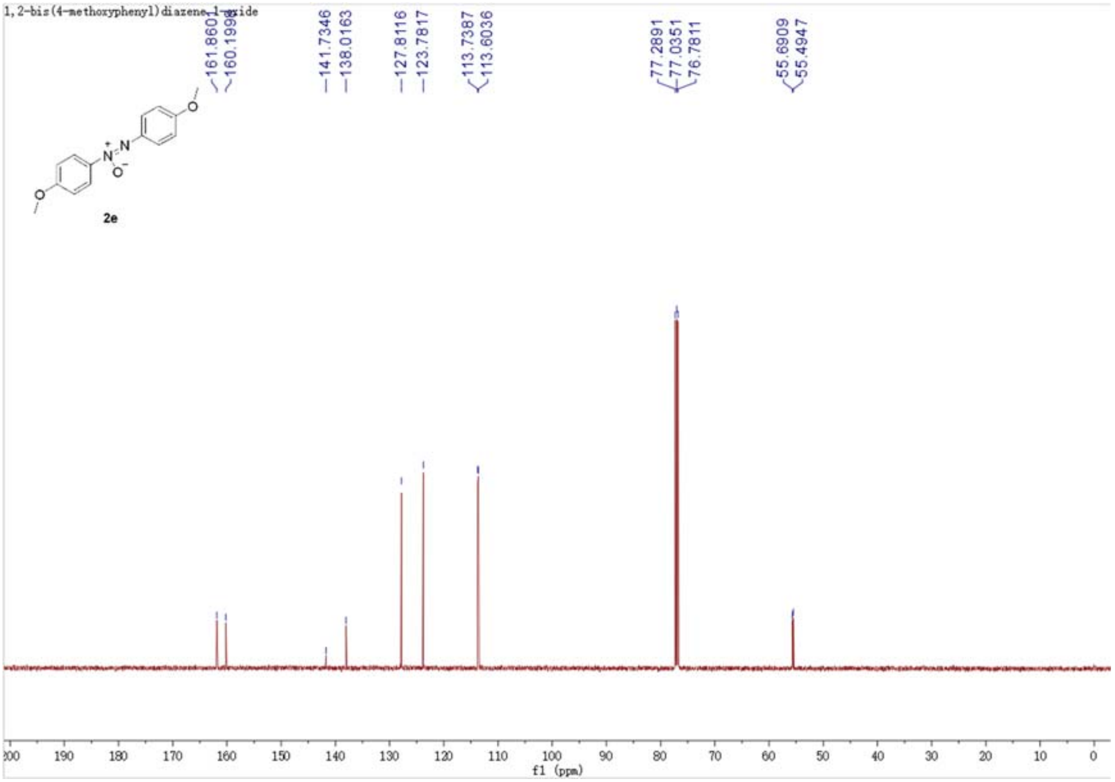
1,2-bis(4-methoxyphenyl)diazene
161.8601
160.1998
141.7346
138.0163
127.8116
123.7817
113.7387
113.6036
77.2891
77.0351
76.7811
55.6909
55.4947
2e
190 180 170 160 150 140 130 120 110 100 90 80 70 60 50 40 30 20 10 0
f1 (ppm)

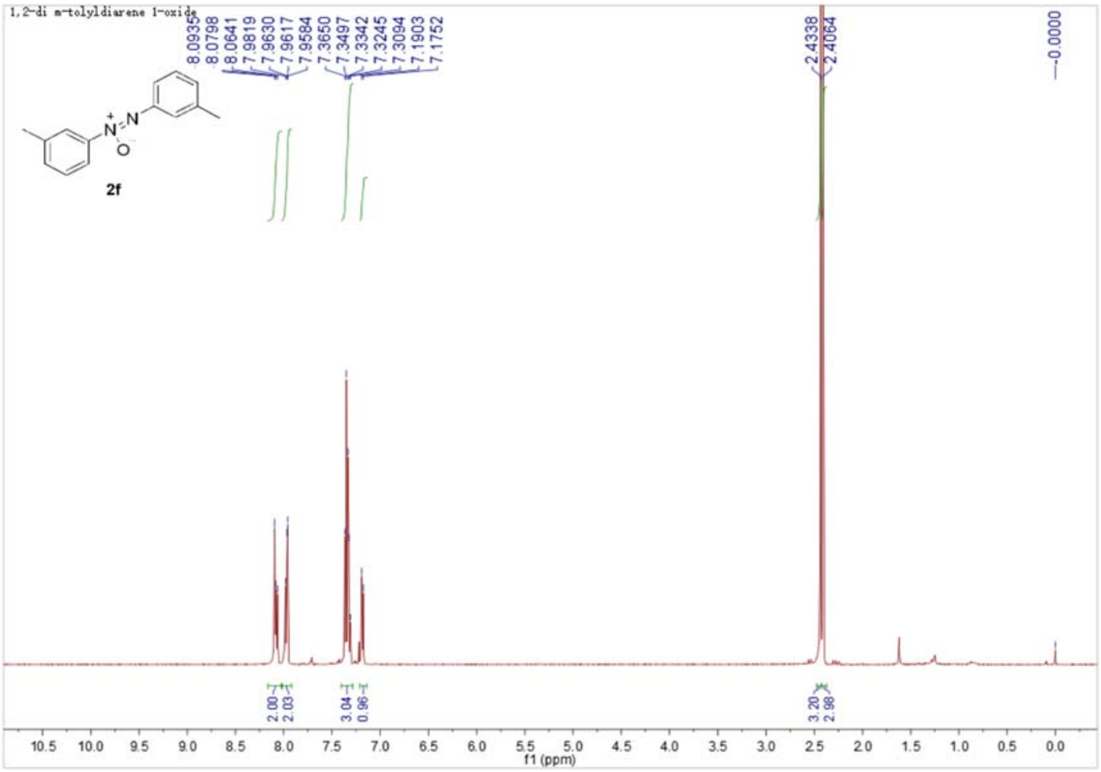
1,2-di m-tolyldiarene 1-oxide
2f
8.0935
8.0798
8.0641
7.9819
7.9630
7.9617
7.9584
7.3650
7.3497
7.3342
7.3245
7.3094
7.1903
7.1752
2.4338
2.4064
-0.0000
2.00
2.03
3.04
0.96
3.20
2.98
f1 (ppm)

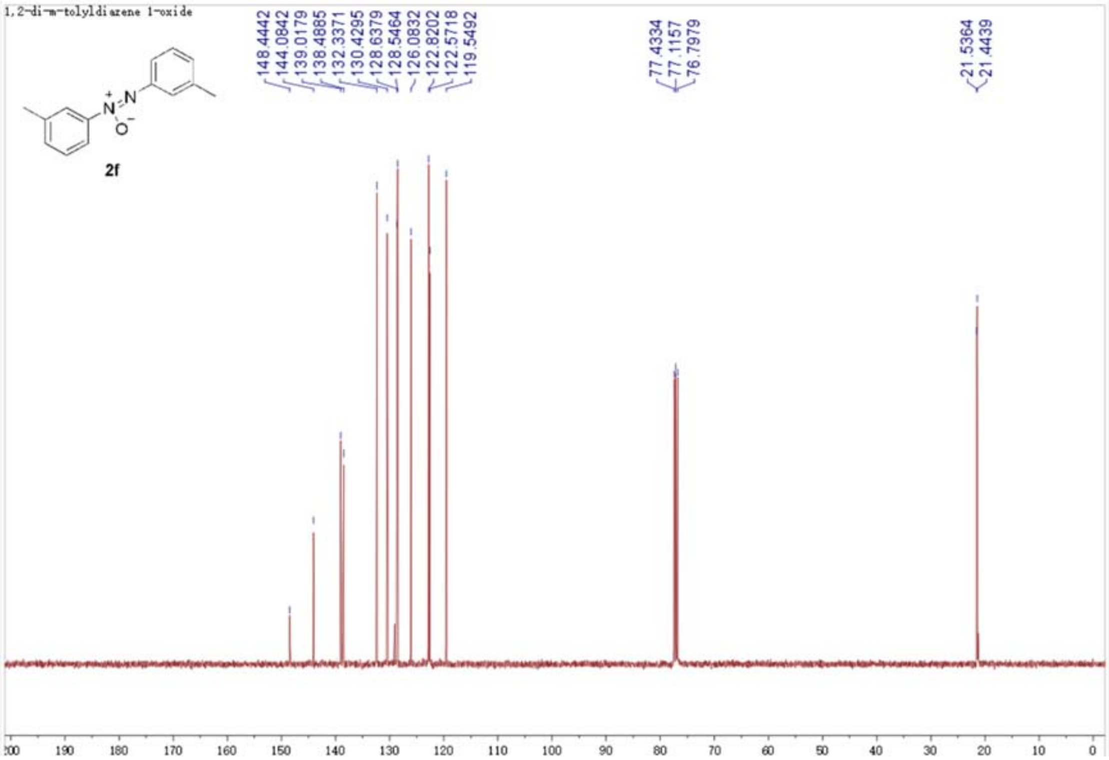
1,2-di-m-tolyldiarene 1-oxide
2f
148.4442
144.0842
139.0179
138.4885
132.3371
130.4295
128.6379
128.5464
126.0832
122.8202
122.5718
119.5492
77.4334
77.1157
76.7979
21.5364
21.4439

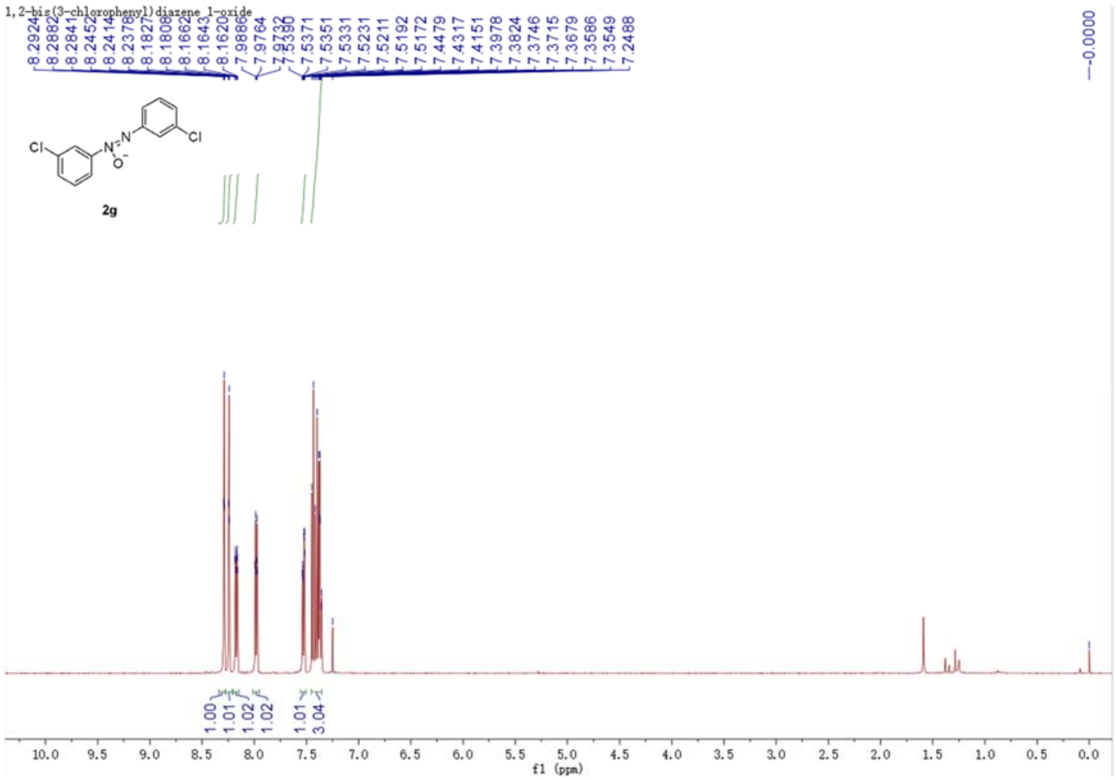
1,2-bis(3-chlorophenyl)diazene 1-oxide
8.2924
8.2882
8.2841
8.2452
8.2414
8.2378
8.1827
8.1808
8.1662
8.1643
8.1620
7.9886
7.9764
7.9732
7.5390
7.5371
7.5351
7.5331
7.5231
7.5211
7.5192
7.5172
7.4479
7.4317
7.4151
7.3978
7.3824
7.3746
7.3715
7.3679
7.3586
7.3549
7.2488
-0.0000
2g
1.00
1.01
1.02
1.02
1.01
3.04
f1 (ppm)

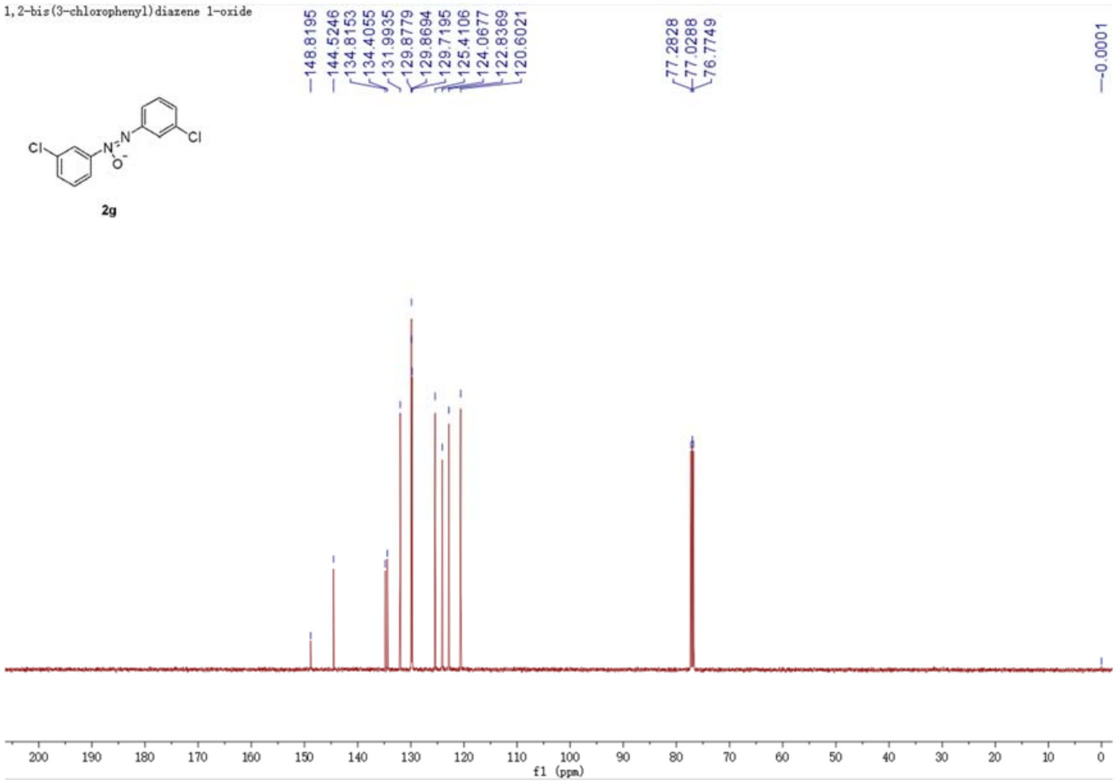
1,2-bis(3-chlorophenyl)diazene 1-oxide
148.8195
144.5246
134.8153
134.4055
131.9935
129.8779
129.8694
129.7195
125.4106
124.0677
122.8369
120.6021
77.2828
77.0288
76.7749
-0.0001
2g
f1 (ppm)

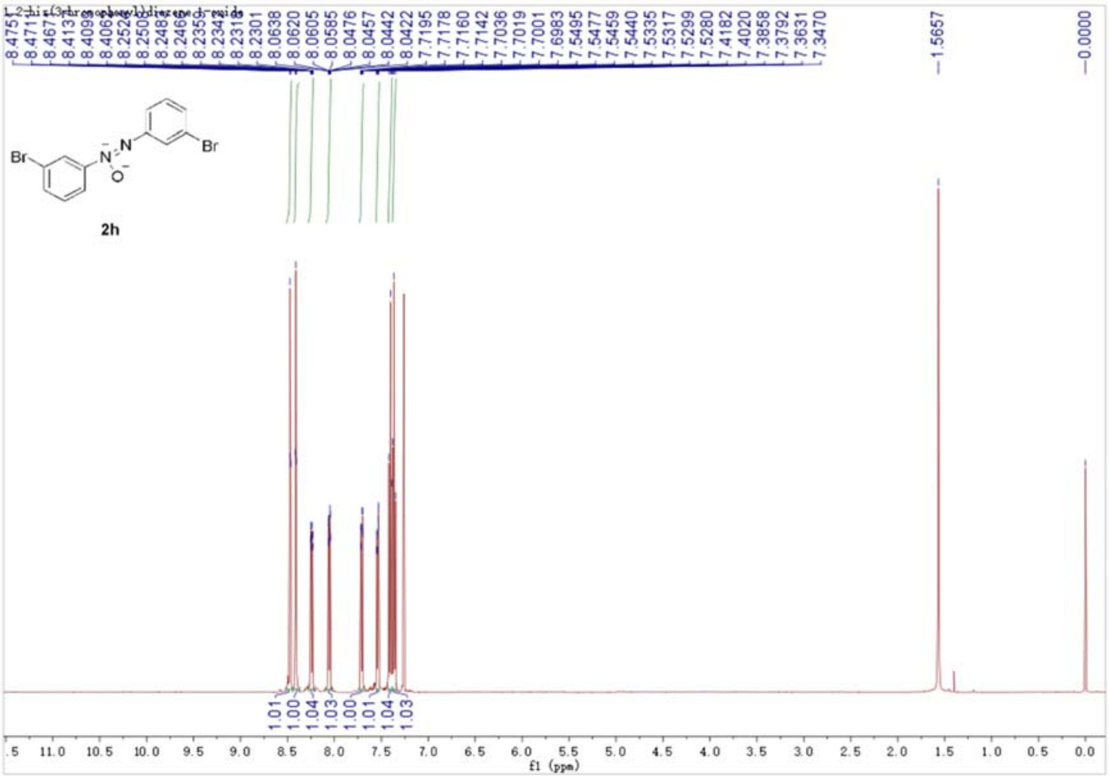
1,2-bis(3-bromophenyl)diazene 1-oxide
2h
f1 (ppm)

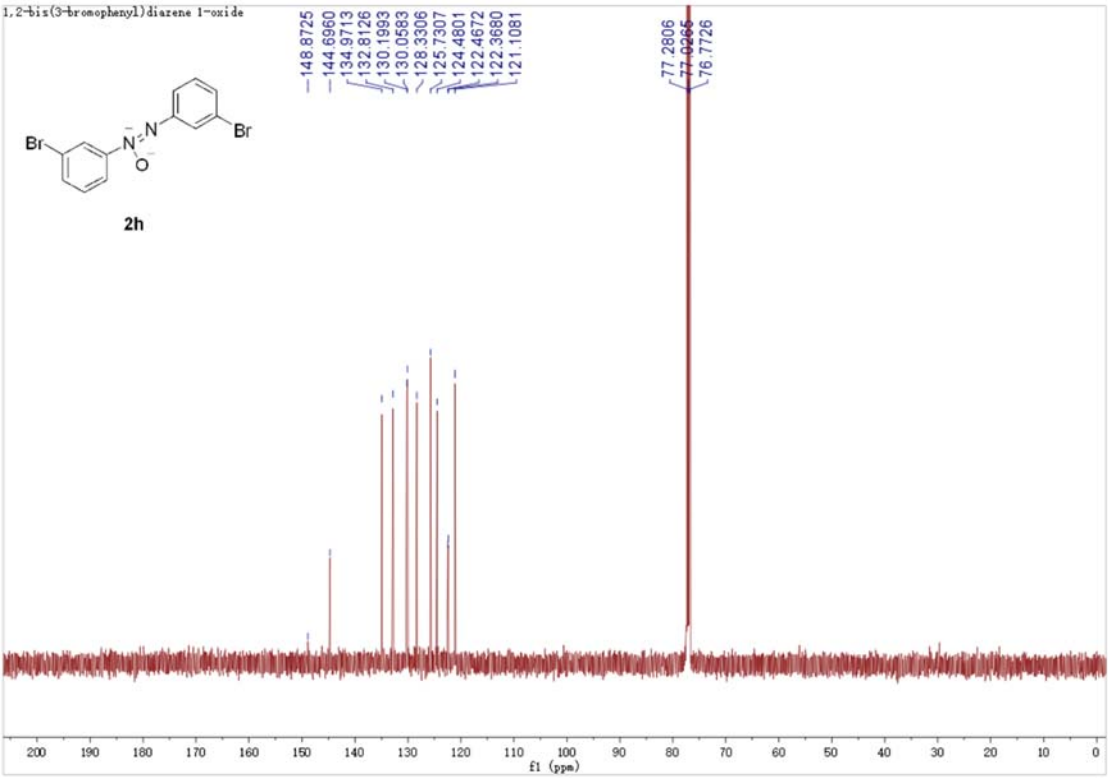
1,2-bis(3-bromophenyl)diazene 1-oxide
148.8725
144.6960
134.9713
132.8126
130.1993
130.0583
128.3306
125.7307
124.4801
122.4672
122.3680
121.1081
77.2806
77.0265
76.7726
2h
f1 (ppm)

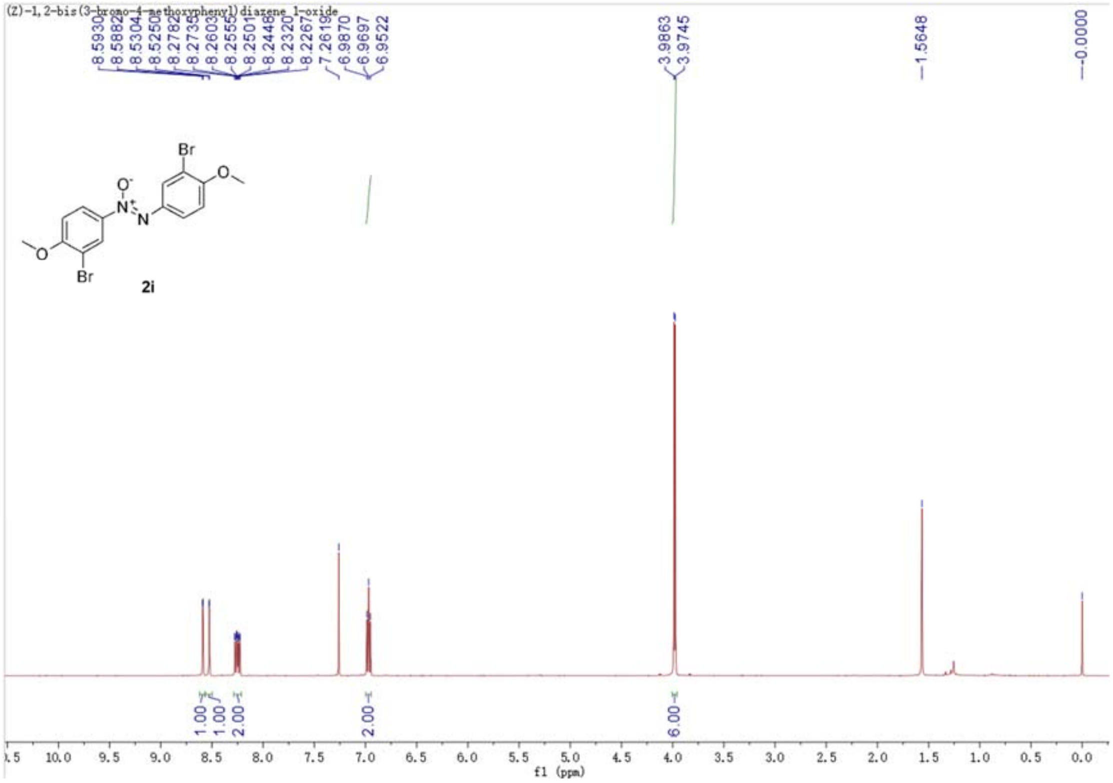
(Z)-1,2-bis(3-bromo-4-methoxyphenyl)diazene 1-oxide
8.5930
8.5882
8.5304
8.5250
8.2782
8.2735
8.2603
8.2555
8.2501
8.2448
8.2320
8.2267
7.2619
6.9870
6.9697
6.9522
3.9863
3.9745
1.5648
0.0000
2i
1.00
1.00
2.00
2.00
6.00
f1 (ppm)

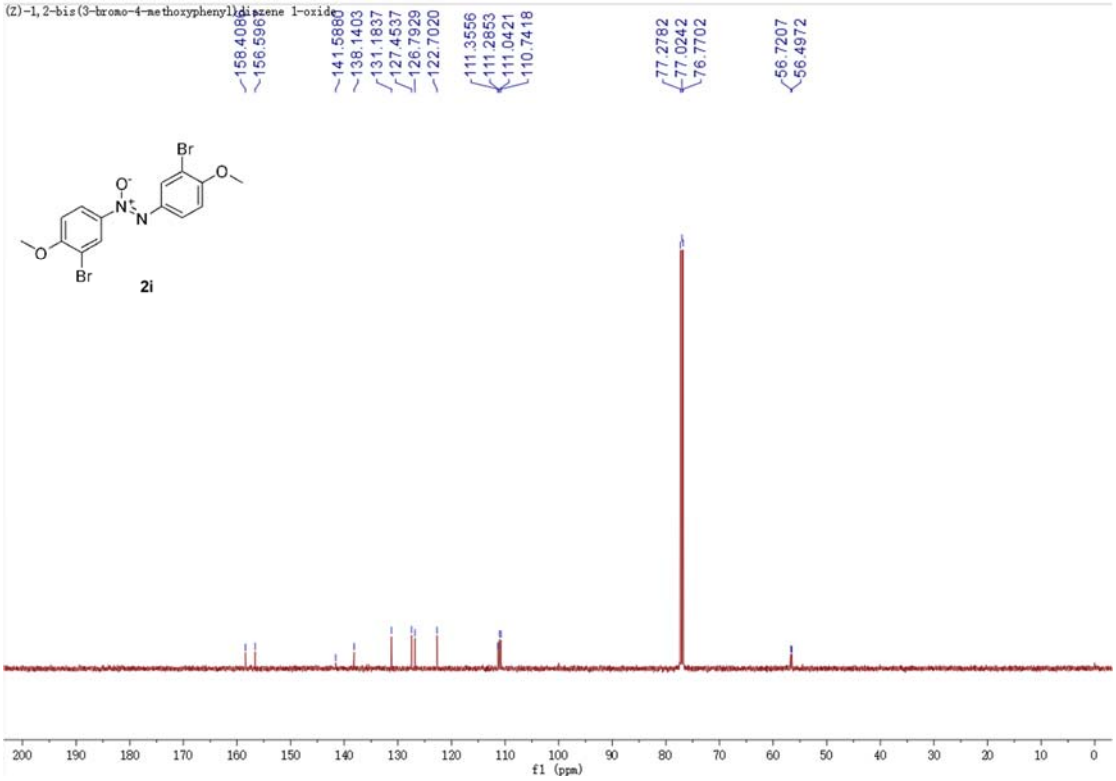
(Z)-1,2-bis(3-bromo-4-methoxyphenyl)diazene 1-oxide
158.4086
156.5963
141.5880
138.1403
131.1837
127.4537
126.7929
122.7020
111.3556
111.2853
111.0421
110.7418
77.2782
77.0242
76.7702
56.7207
56.4972
2i
f1 (ppm)

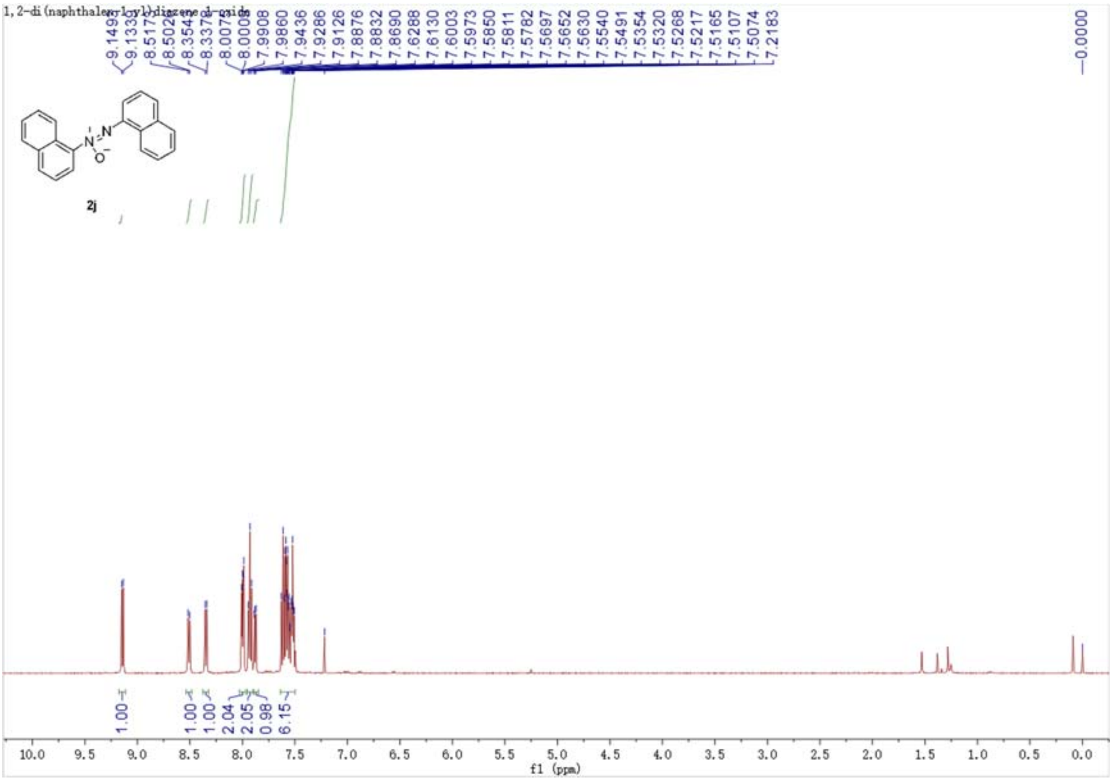
1,2-di(naphthalen-1-yl)diazene 1-oxide
2j
f1 (ppm)

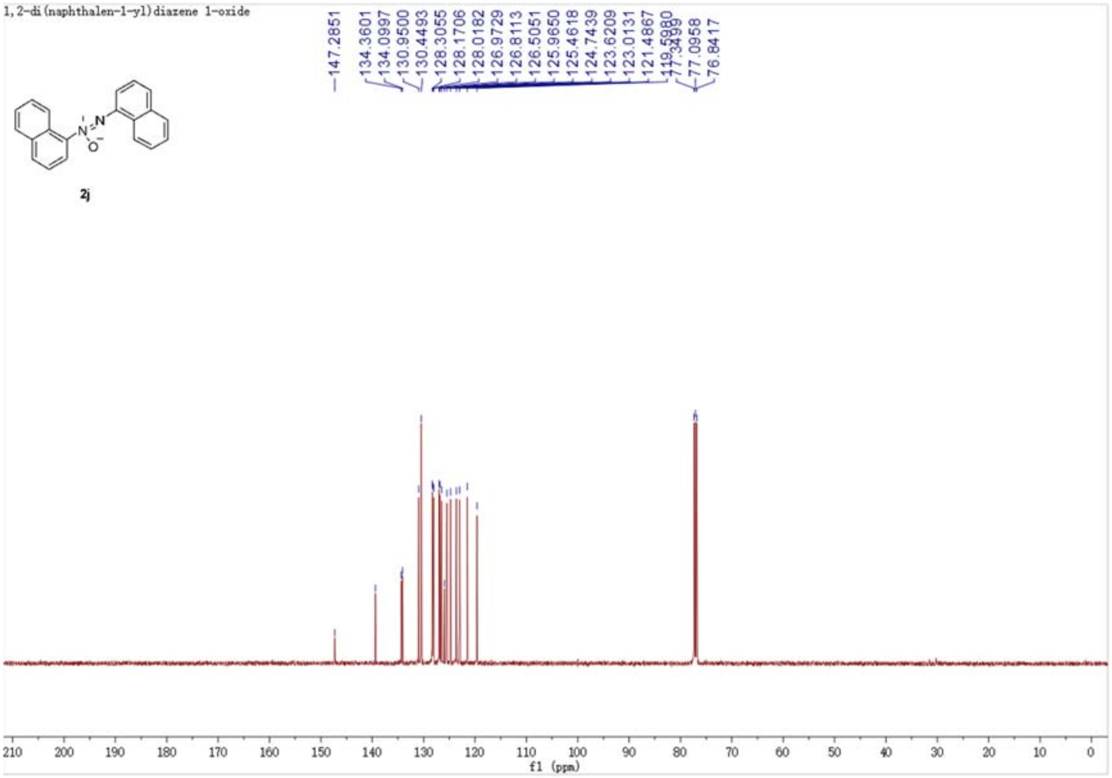
1,2-di(naphthalen-1-yl)diazene 1-oxide
2j
f1 (ppm)

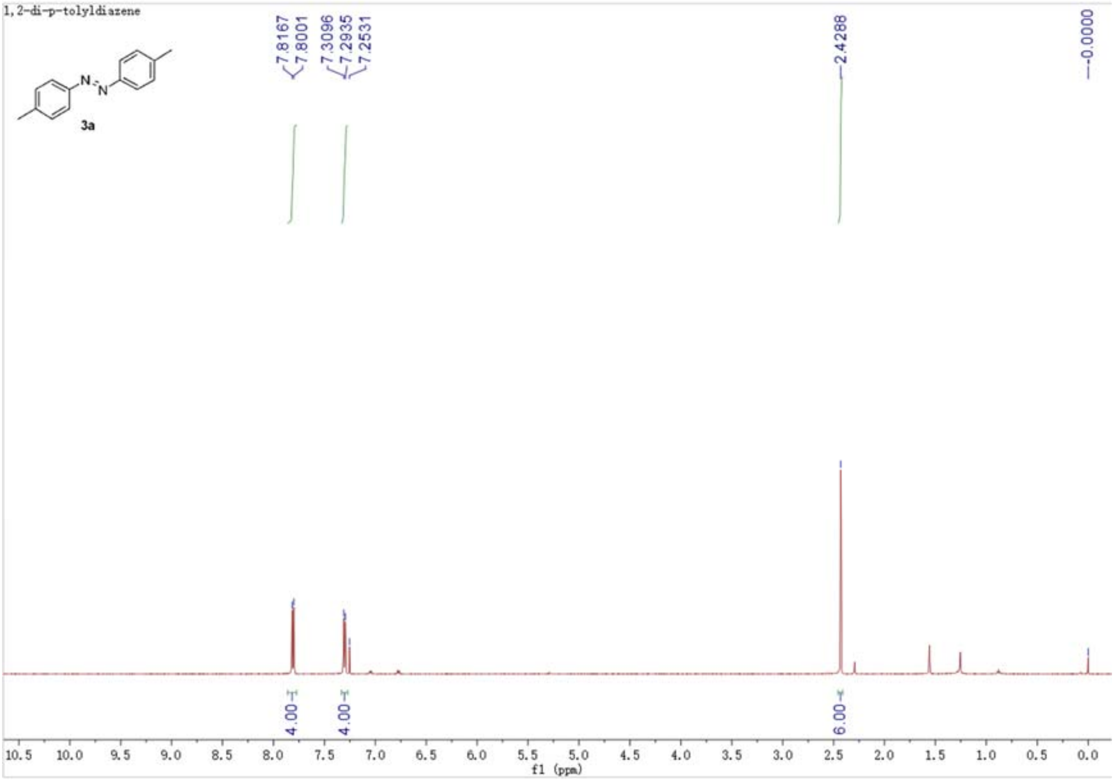
1,2-di-p-tolyldiazene
3a
7.8167
7.8001
7.3096
7.2935
7.2531
2.4288
0.0000
4.00
4.00
6.00
f1 (ppm)

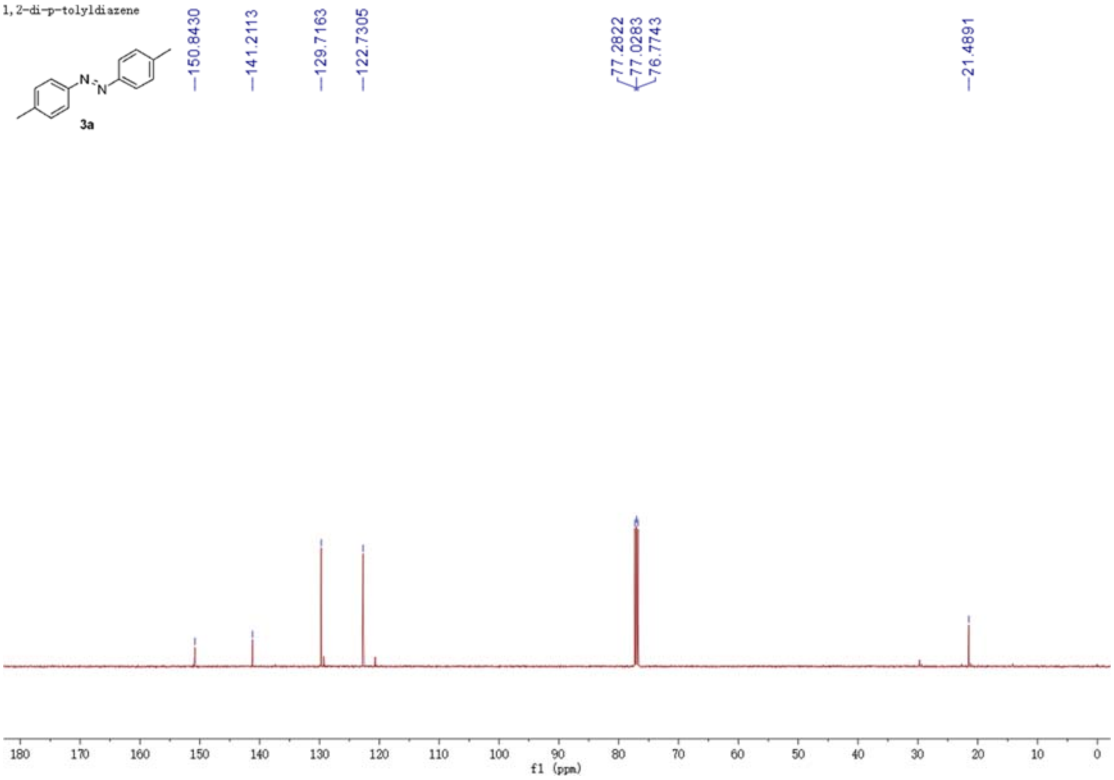
1,2-di-p-tolyldiazene
3a
150.8430
141.2113
129.7163
122.7305
77.2822
77.0283
76.7743
21.4891
f1 (ppm)

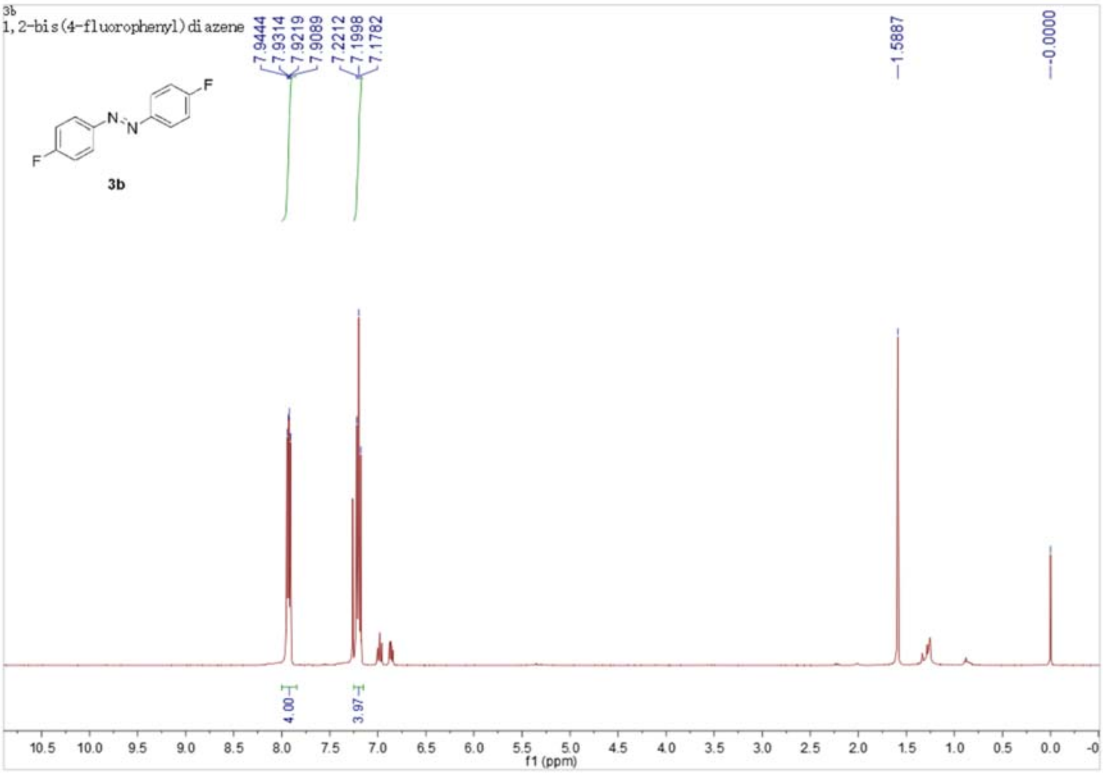
3b
1,2-bis(4-fluorophenyl)diazene
7.9444
7.9314
7.9219
7.9089
7.2212
7.1998
7.1782
1.5887
0.0000
3b
4.00
3.97
f1 (ppm)

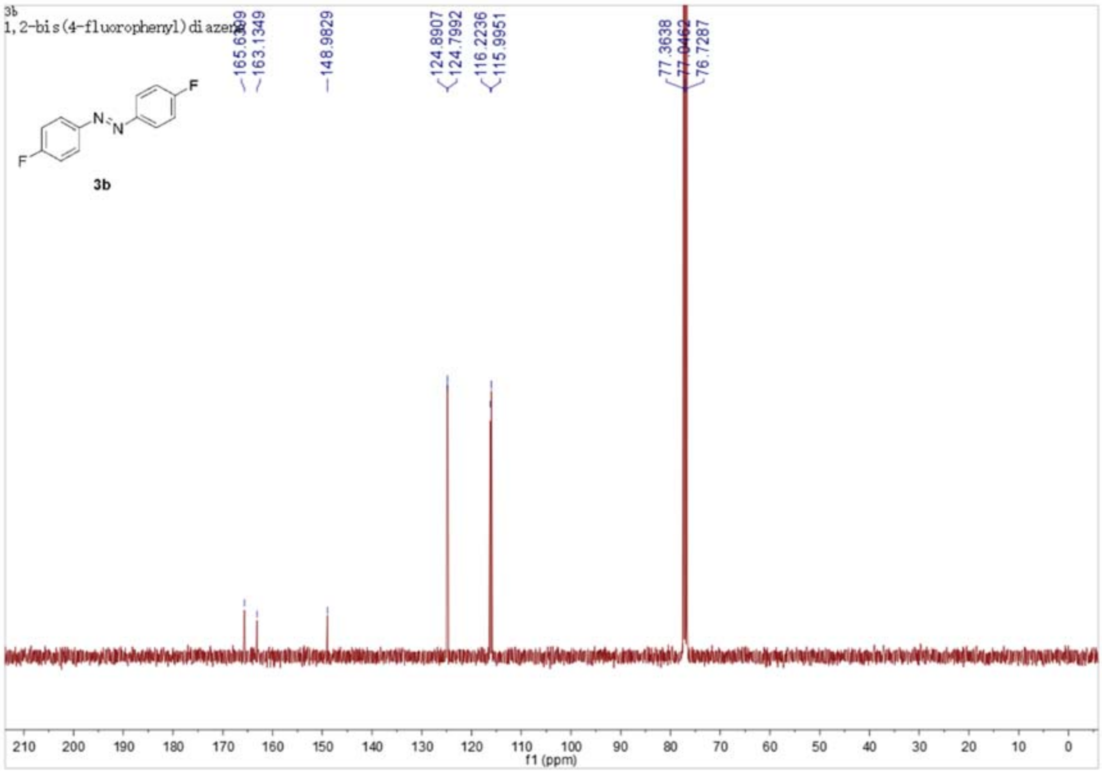
3b
1,2-bis(4-fluorophenyl)diazene
165.6389
163.1349
148.9829
124.8907
124.7992
116.2236
115.9951
77.3638
77.0462
76.7287
3b
f1 (ppm)

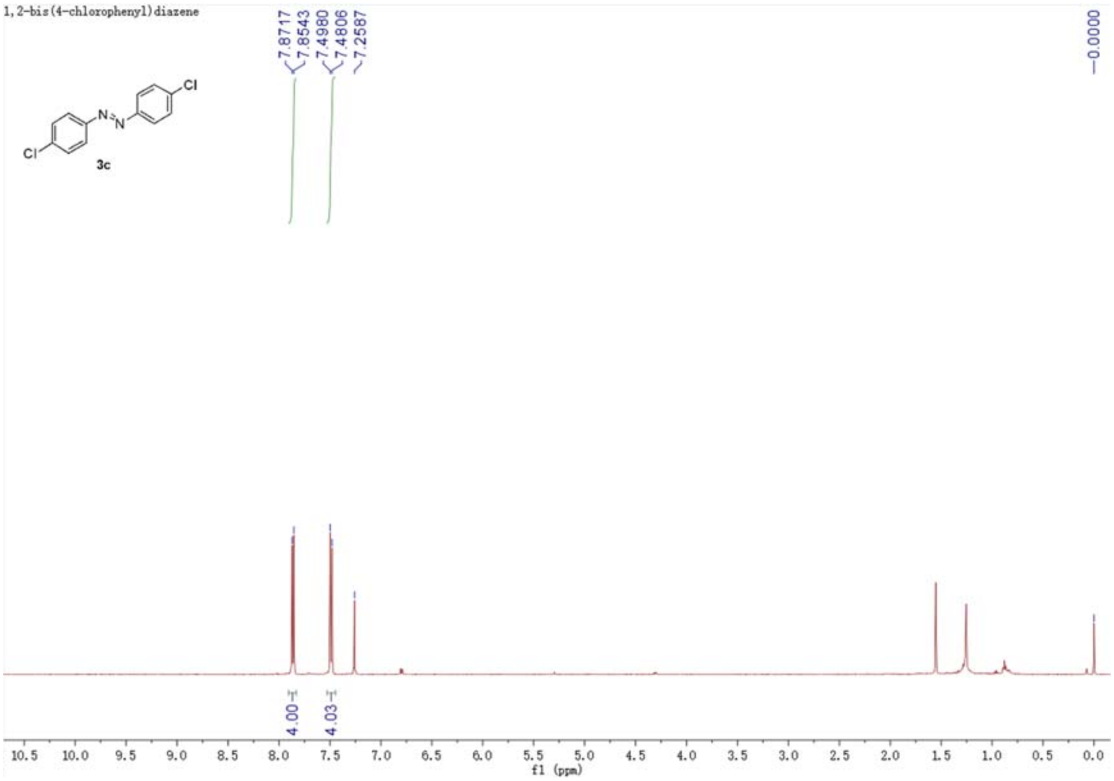
1,2-bis(4-chlorophenyl)diazene
7.8717
7.8543
7.4980
7.4806
7.2587
0.0000
Cl
N
N
Cl
3c
4.00
4.03
10.5 10.0 9.5 9.0 8.5 8.0 7.5 7.0 6.5 6.0 5.5 5.0 4.5 4.0 3.5 3.0 2.5 2.0 1.5 1.0 0.5 0.0
f1 (ppm)

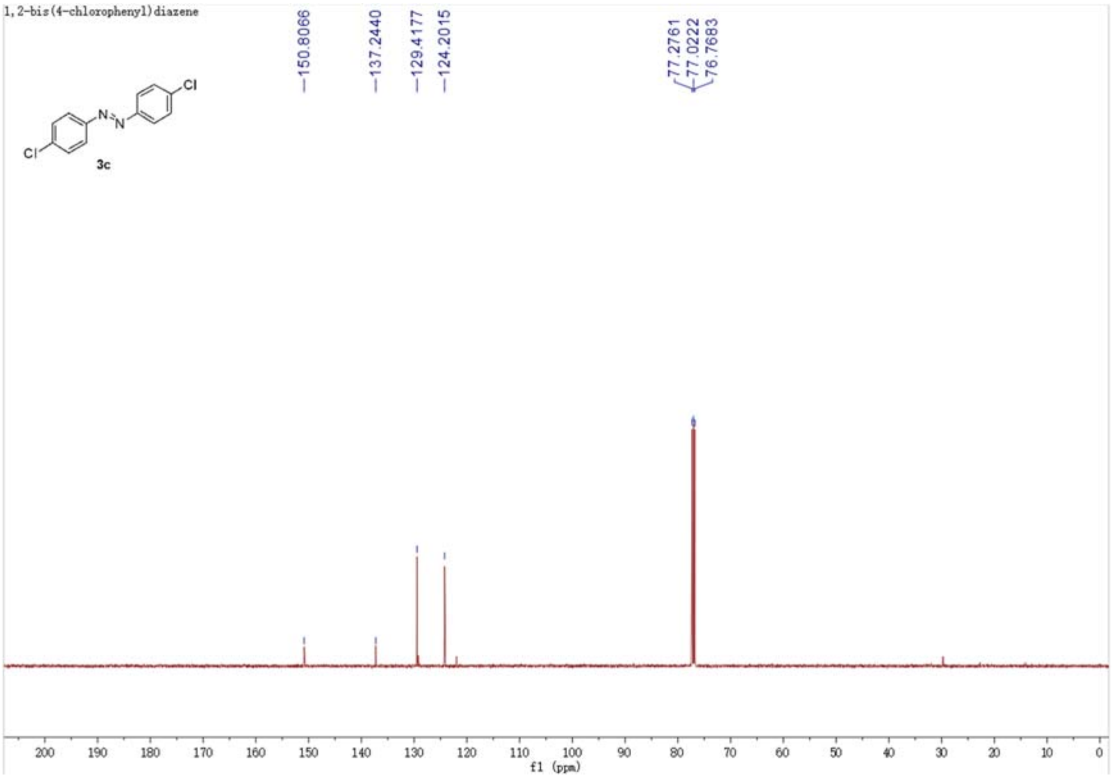
1,2-bis(4-chlorophenyl)diazene
150.8066
137.2440
129.4177
124.2015
77.2761
77.0222
76.7683
Cl
N
N
Cl
3c
200 190 180 170 160 150 140 130 120 110 100 90 80 70 60 50 40 30 20 10 0
f1 (ppm)

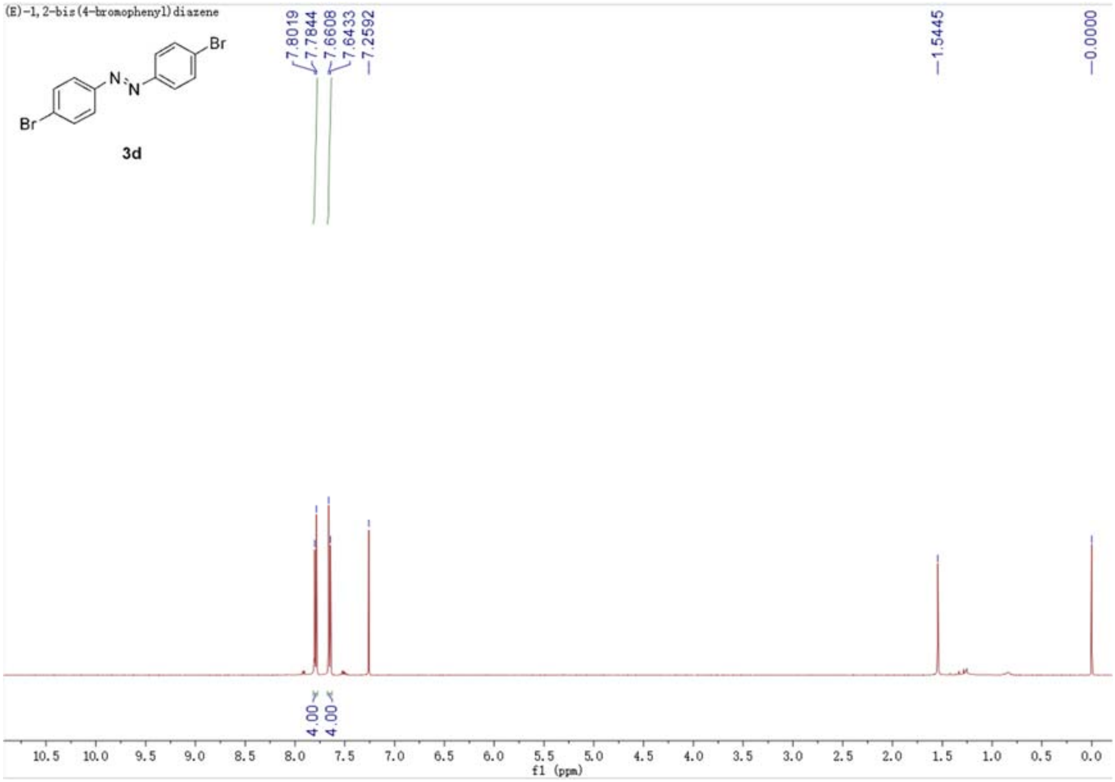
(E)-1,2-bis(4-bromophenyl)diazene
Br
N
N
Br
3d
7.8019
7.7844
7.6608
7.6433
7.2592
1.5445
0.0000
4.00
4.00
10.5 10.0 9.5 9.0 8.5 8.0 7.5 7.0 6.5 6.0 5.5 5.0 4.5 4.0 3.5 3.0 2.5 2.0 1.5 1.0 0.5 0.0
f1 (ppm)

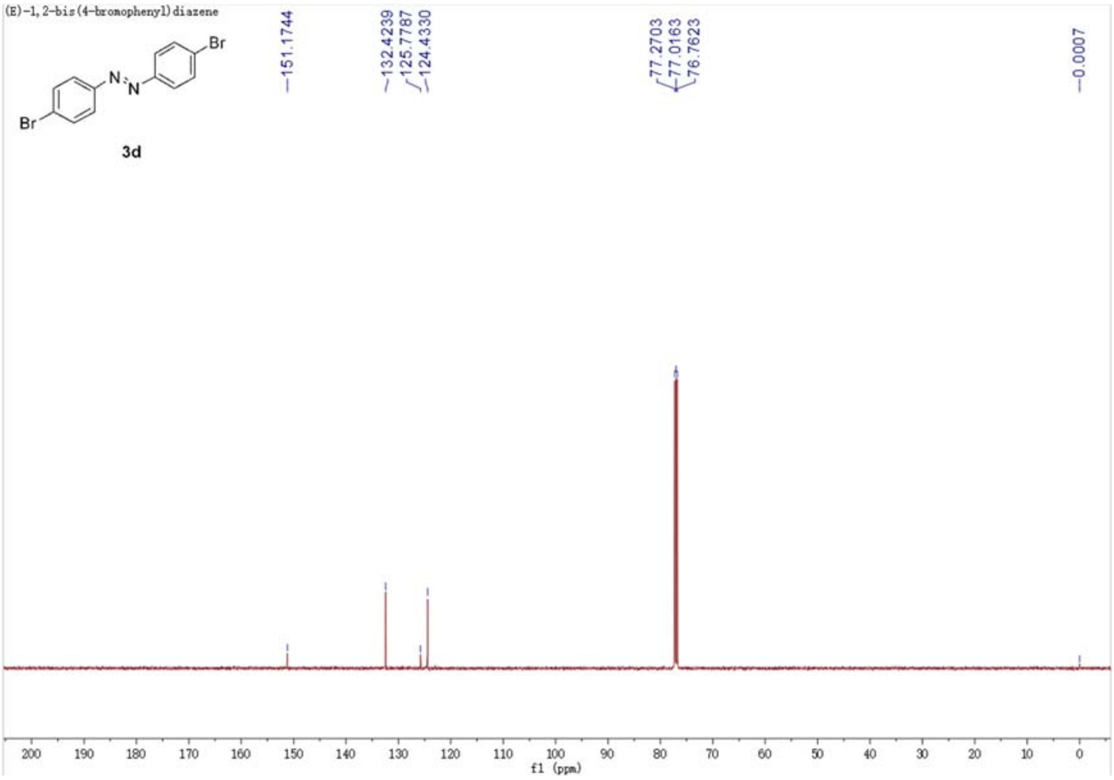
(E)-1,2-bis(4-bromophenyl)diazene
Br
N
N
Br
3d
151.1744
132.4239
125.7787
124.4330
77.2703
77.0163
76.7623
0.0007
200 190 180 170 160 150 140 130 120 110 100 90 80 70 60 50 40 30 20 10 0
f1 (ppm)

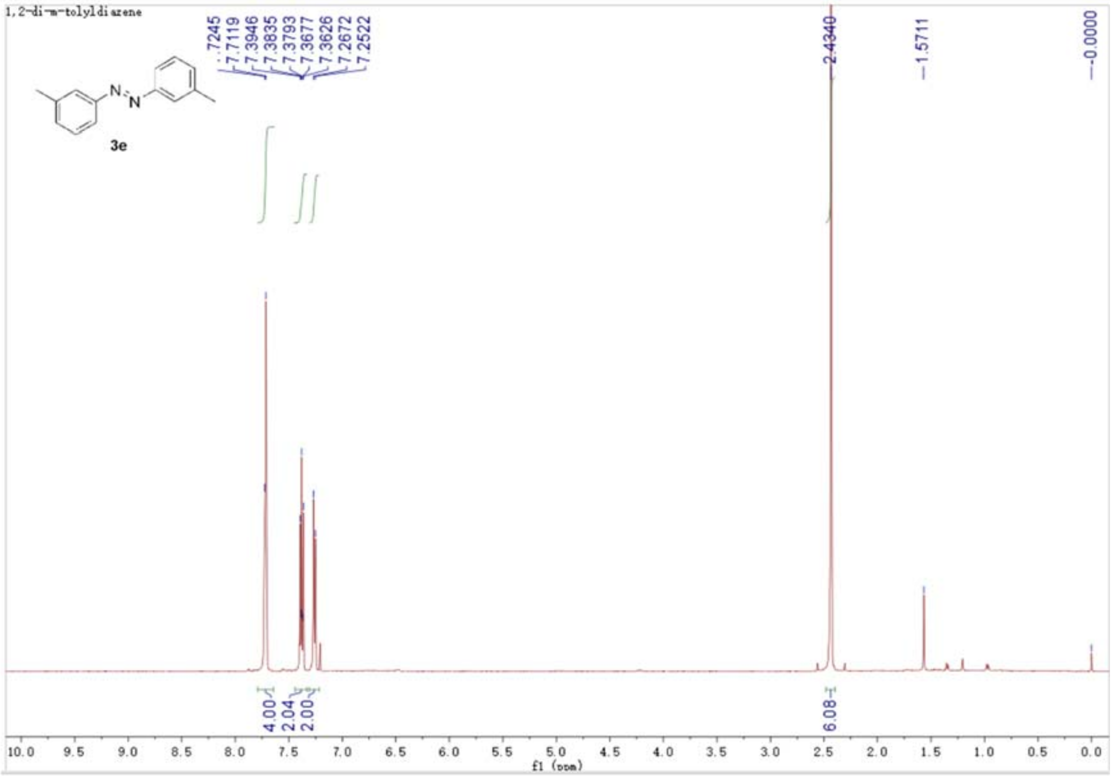
1,2-di-m-tolyldiazene
3e
7.7245
7.7119
7.3946
7.3835
7.3793
7.3677
7.3626
7.2672
7.2522
2.4340
1.5711
0.0000
4.00
2.04
2.00
6.08
f1 (ppm)

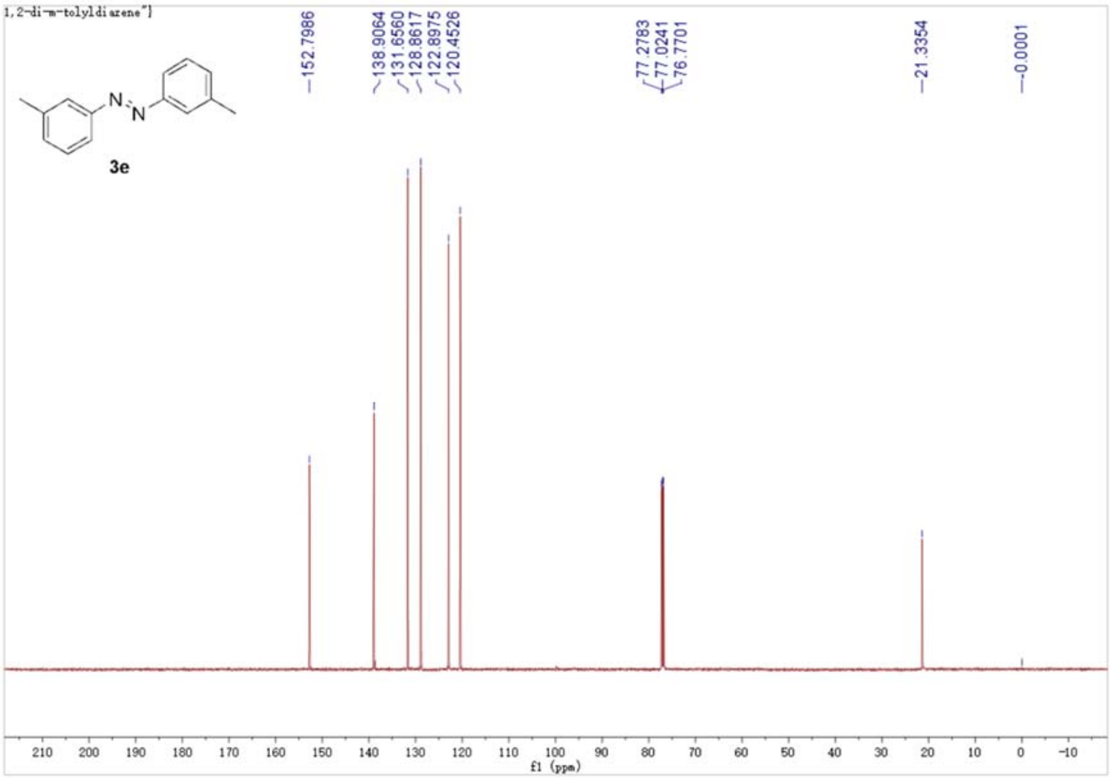
1,2-di-m-tolyldiazene"]
3e
152.7986
138.9064
131.6560
128.8617
122.8975
120.4526
77.2783
77.0241
76.7701
21.3354
-0.0001
f1 (ppm)

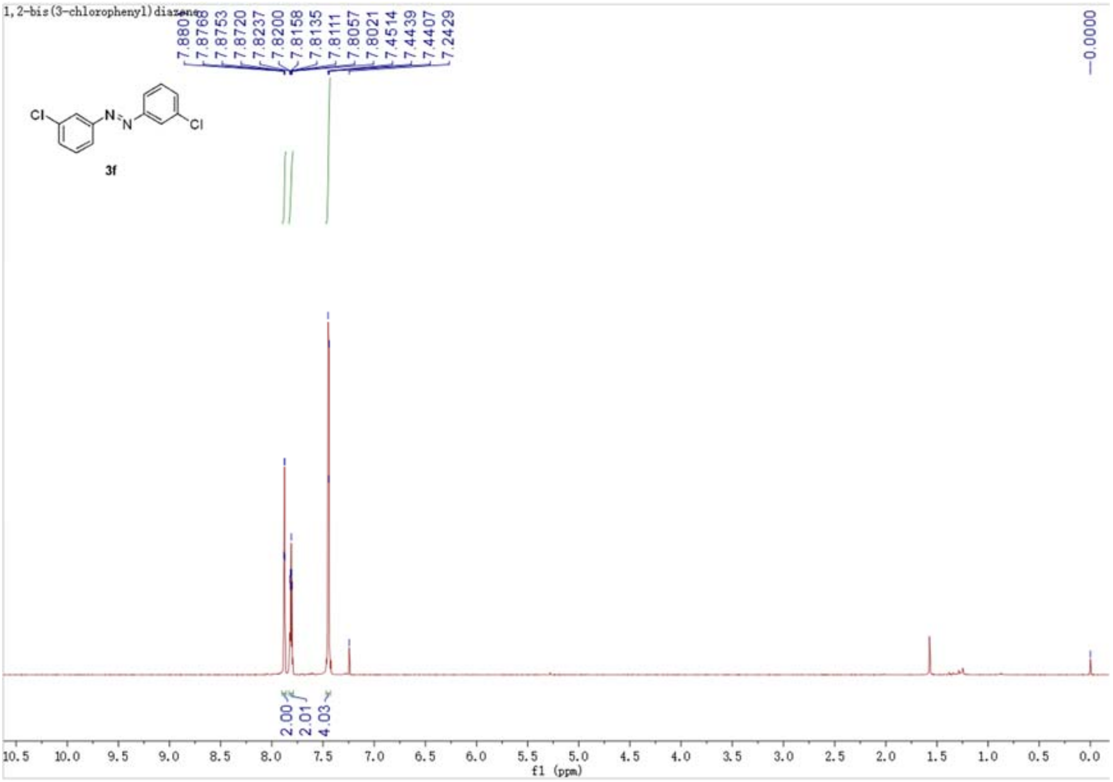
1,2-bis(3-chlorophenyl)diazene
7.8801
7.8768
7.8753
7.8720
7.8237
7.8200
7.8158
7.8135
7.8111
7.8057
7.8021
7.4514
7.4439
7.4407
7.2429
0.0000
3f
2.00
2.01
4.03
f1 (ppm)

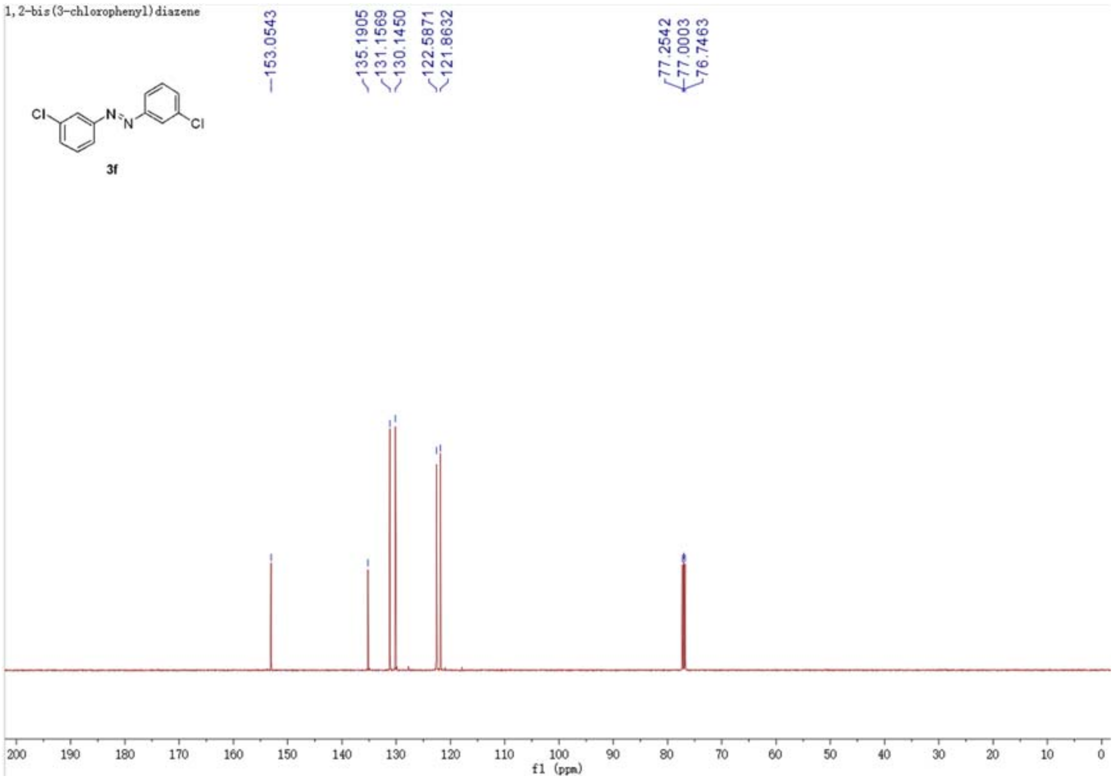
1,2-bis(3-chlorophenyl)diazene
153.0543
135.1905
131.1569
130.1450
122.5871
121.8632
77.2542
77.0003
76.7463
3f
f1 (ppm)

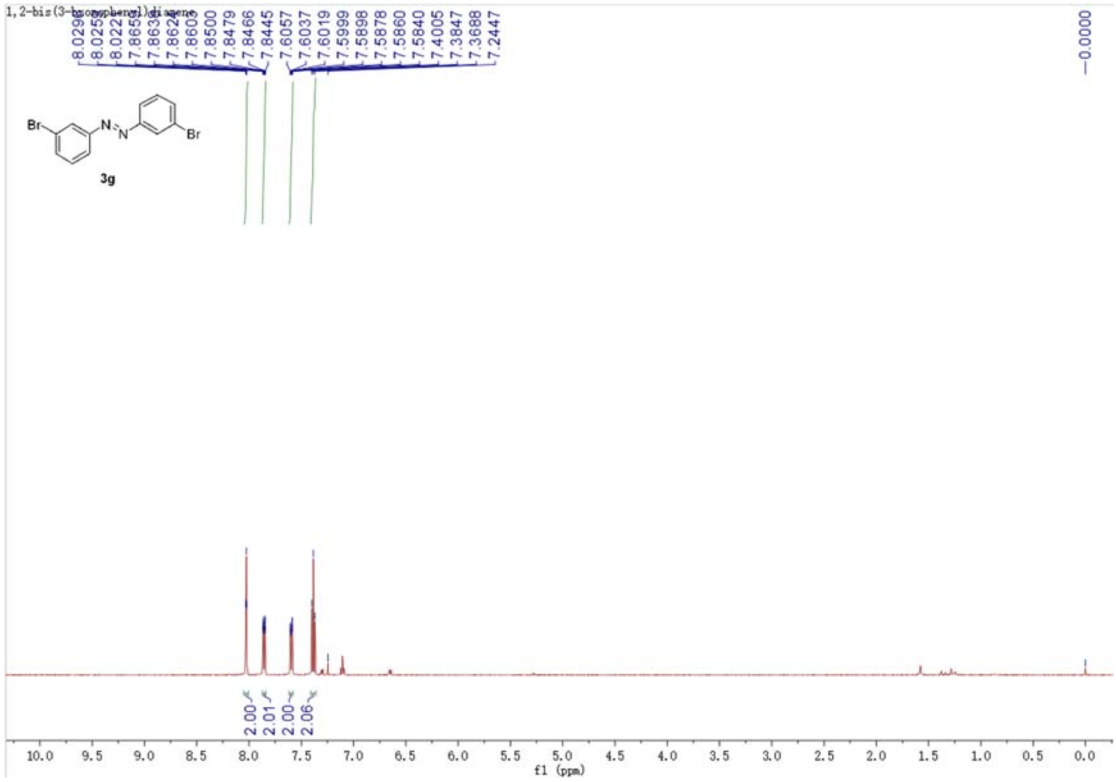
1,2-bis(3-bromophenyl)diazene
8.0296
7.8466
7.8445
7.6057
7.6037
7.6019
7.5999
7.5898
7.5878
7.5860
7.5840
7.4005
7.3847
7.3688
7.2447
0.0000
3g
2.00
2.01
2.00
2.06
f1 (ppm)

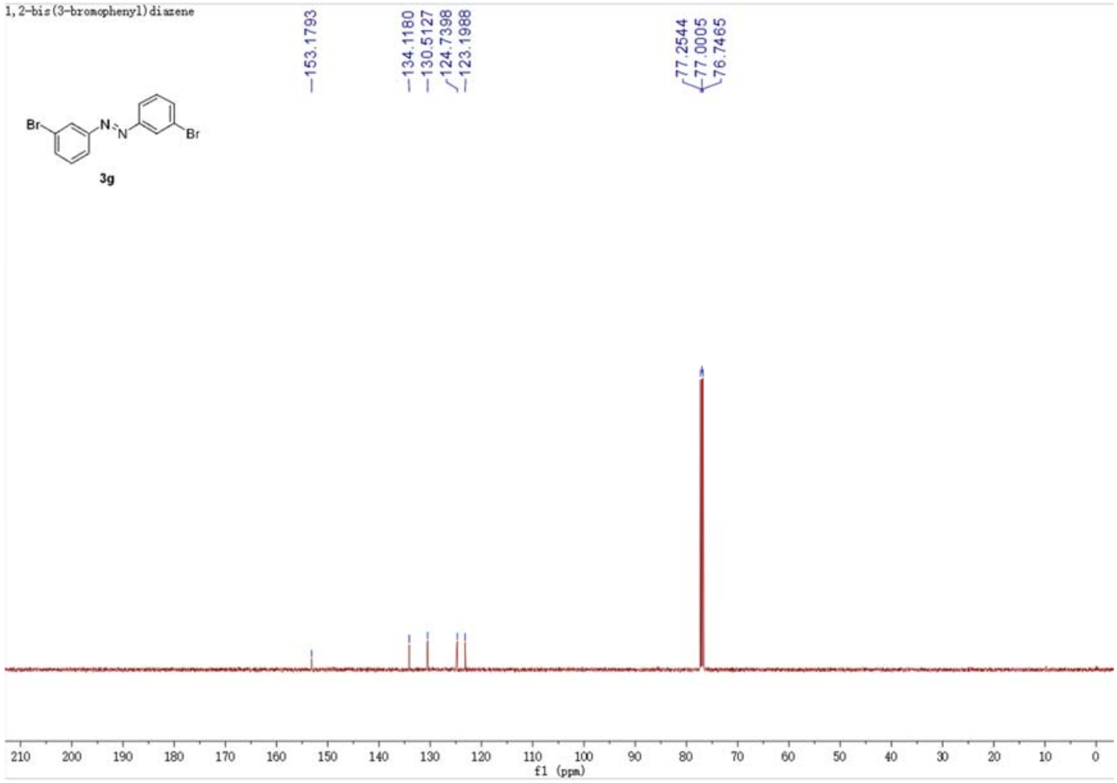
1,2-bis(3-bromophenyl)diazene
153.1793
134.1180
130.5127
124.7398
123.1988
77.2544
77.0005
76.7465
3g
f1 (ppm)

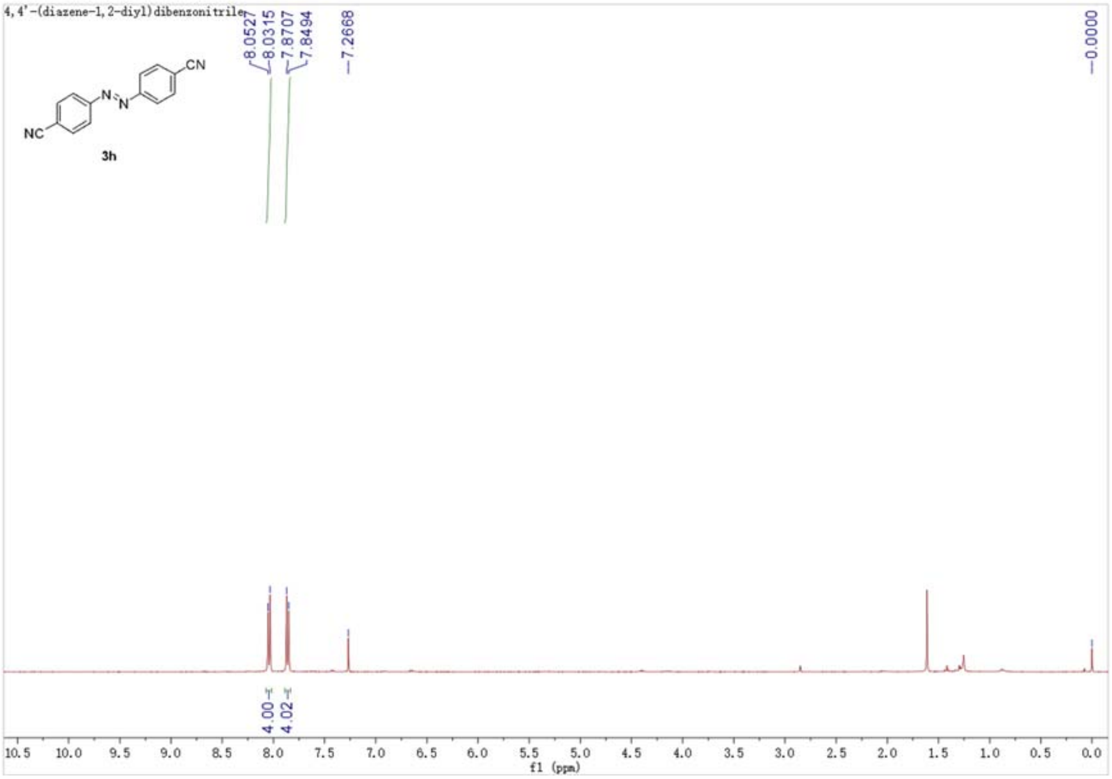
4,4'-(diazene-1,2-diyl)dibenzonitrile
8.0527
8.0315
7.8707
7.8494
7.2668
0.0000
NC
CN
3h
4.00
4.02
f1 (ppm)

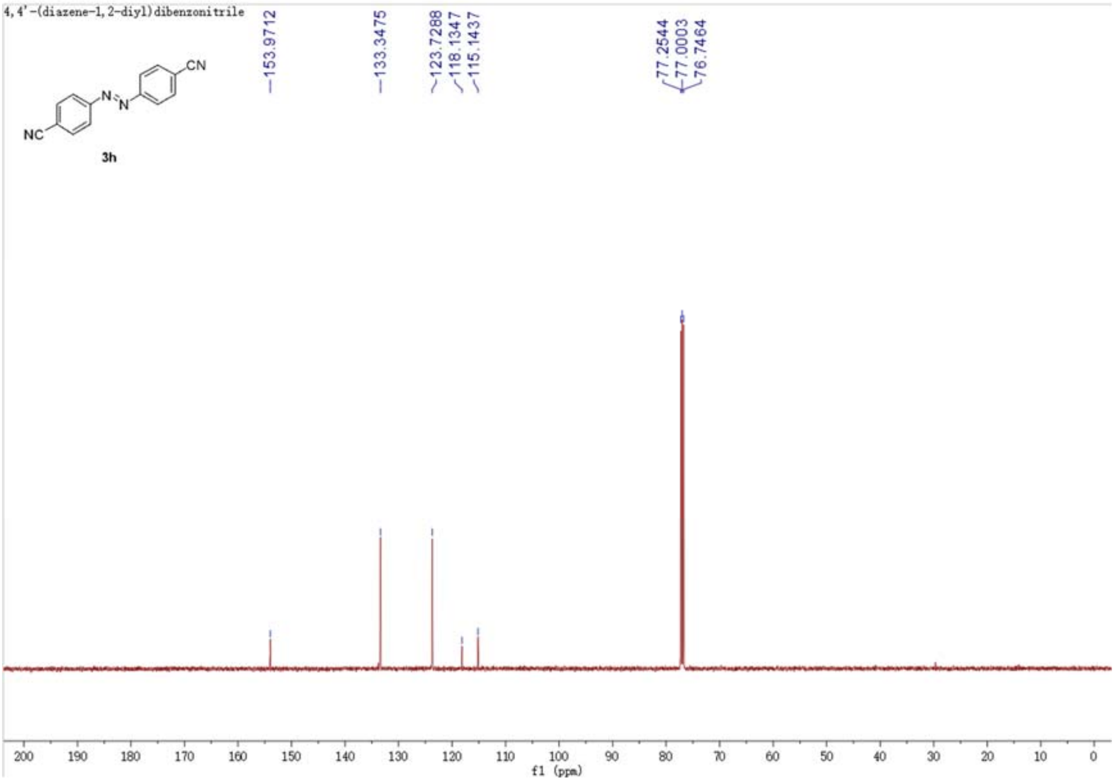
4,4'-(diazene-1,2-diyl)dibenzonitrile
153.9712
133.3475
123.7288
118.1347
115.1437
77.2544
77.0003
76.7464
NC
CN
3h
f1 (ppm)

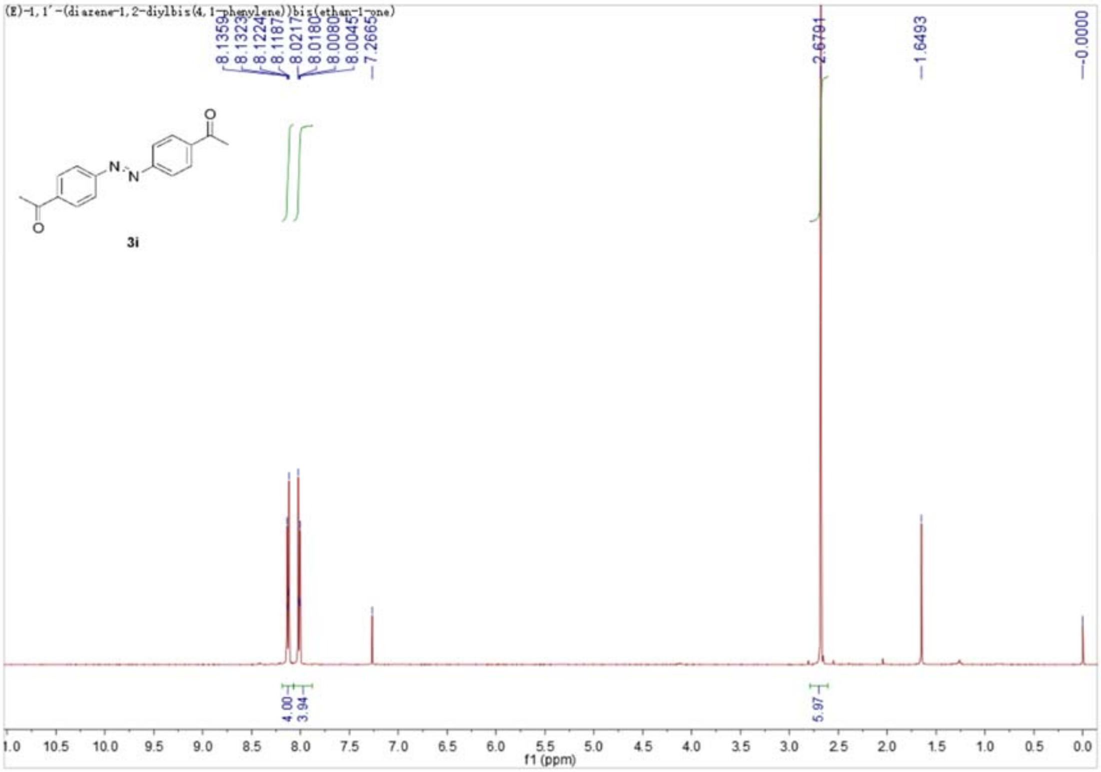
(E)-1,1'-(diazene-1,2-diylbis(4,1-phenylene))bis(ethan-1-one)
8.1359
8.1323
8.1224
8.1187
8.0217
8.0180
8.0080
8.0045
7.2665
2.6791
1.6493
0.0000
3i
4.00
3.94
5.97
f1 (ppm)

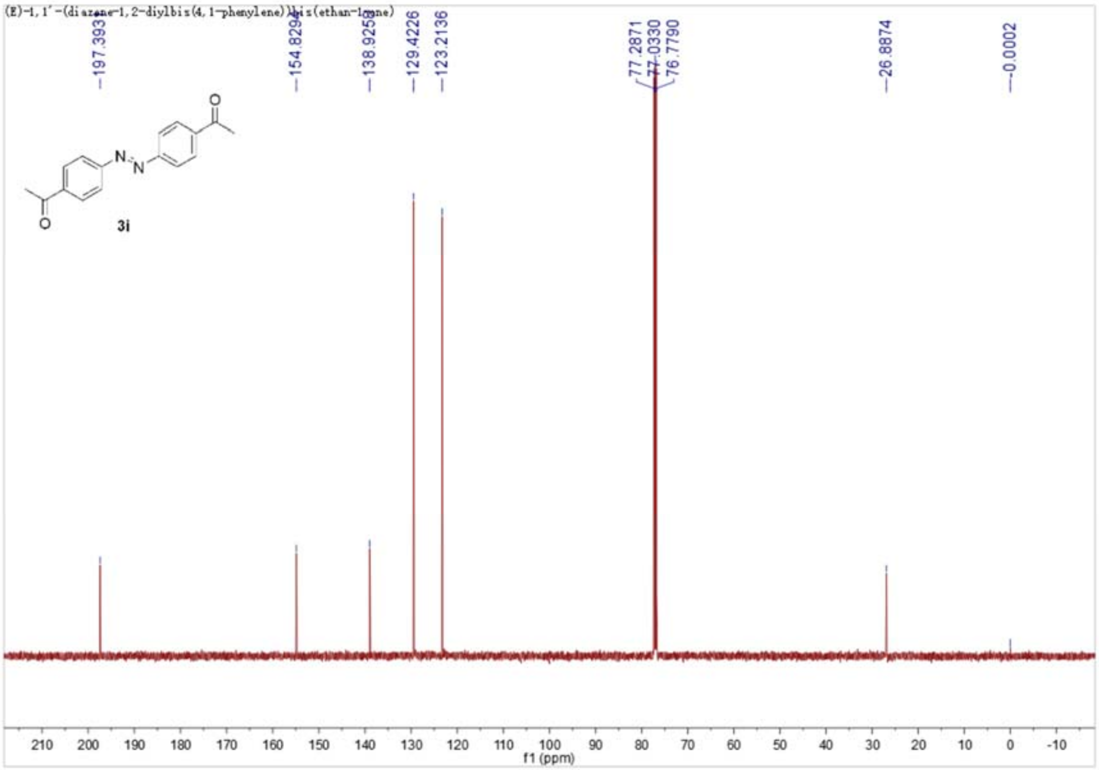
(E)-1,1'-(diazene-1,2-diylbis(4,1-phenylene))bis(ethan-1-one)
197.3931
154.8294
138.9250
129.4226
123.2136
77.2871
77.0330
76.7790
26.8874
0.0002
3i
f1 (ppm)

dimethyl 4,4'-(diazene-1,2-diyl)dibenzoate

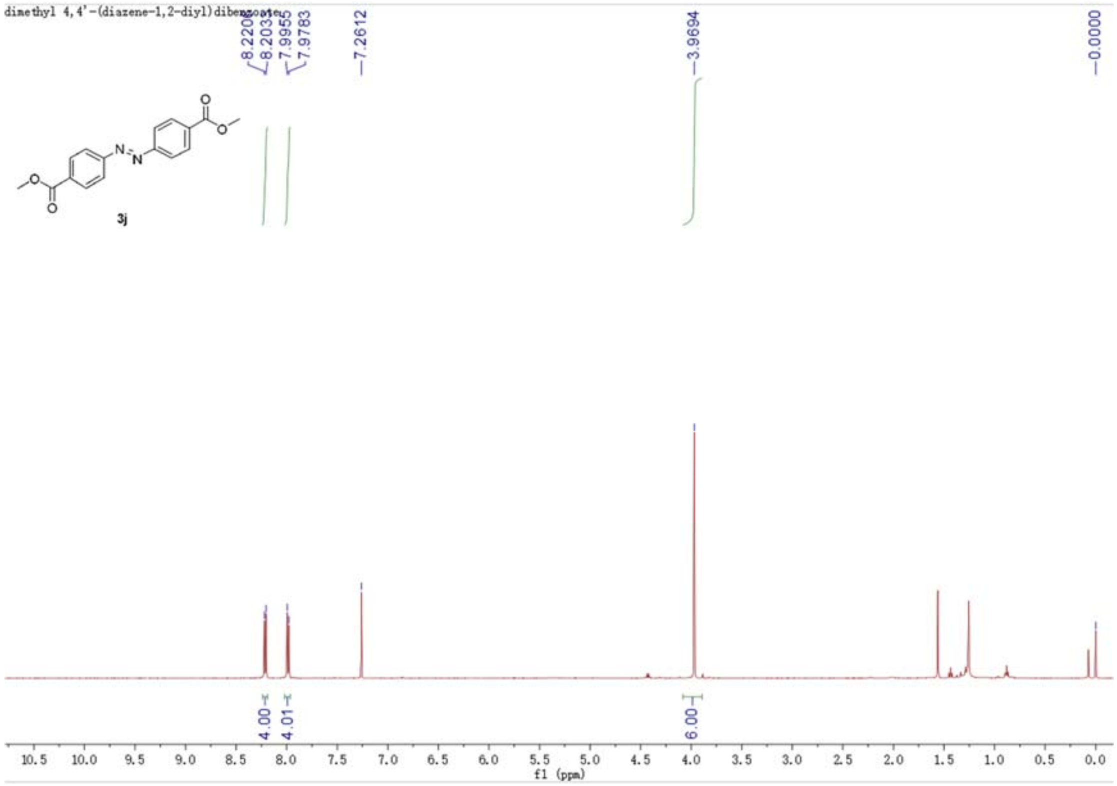

dimethyl 4,4'-(diazene-1,2-diyl)dibenzoate

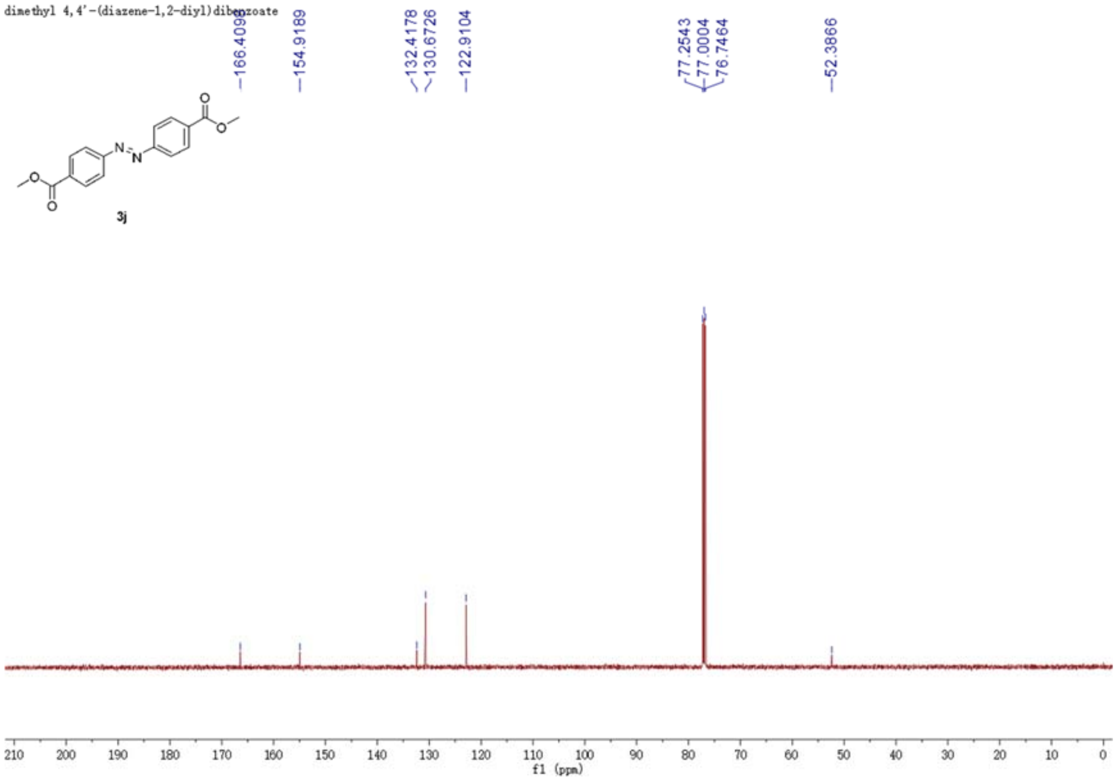

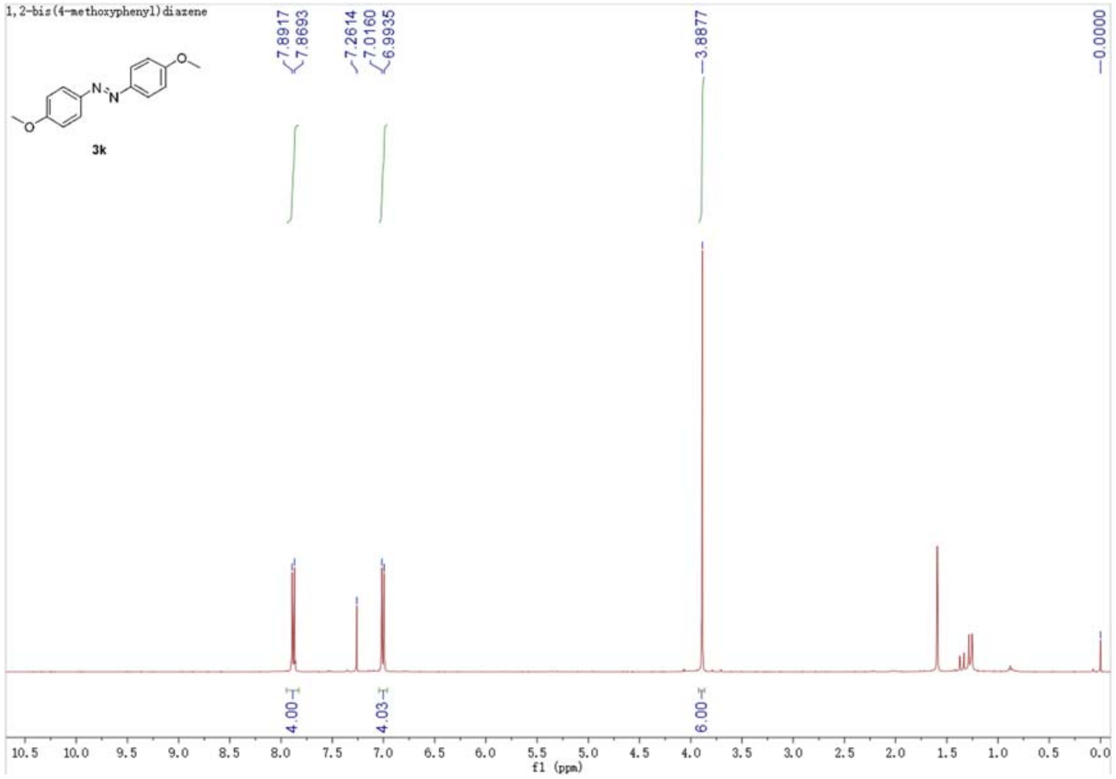
1,2-bis(4-methoxyphenyl)diazene
3k
7.8917
7.8693
7.2614
7.0160
6.9935
3.8877
0.0000
4.00
4.03
6.00
10.5 10.0 9.5 9.0 8.5 8.0 7.5 7.0 6.5 6.0 5.5 5.0 4.5 4.0 3.5 3.0 2.5 2.0 1.5 1.0 0.5 0.0
f1 (ppm)

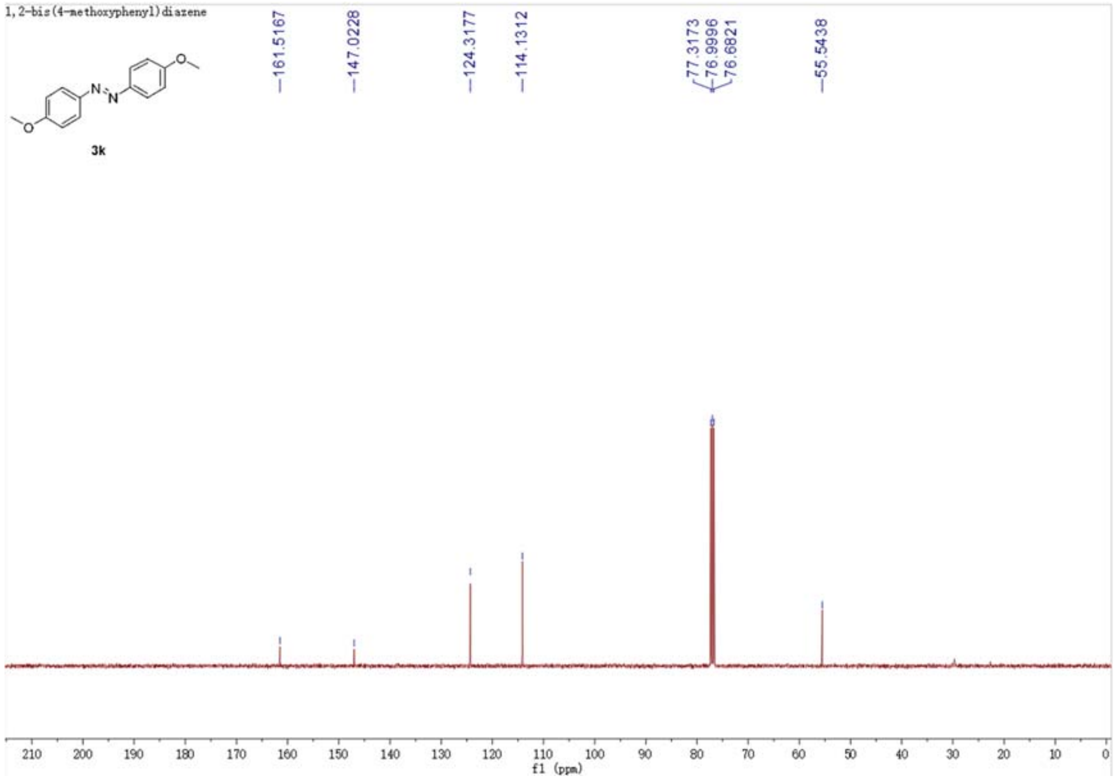
1,2-bis(4-methoxyphenyl)diazene
3k
161.5167
147.0228
124.3177
114.1312
77.3173
76.9996
76.6821
55.5438
210 200 190 180 170 160 150 140 130 120 110 100 90 80 70 60 50 40 30 20 10 0
f1 (ppm)

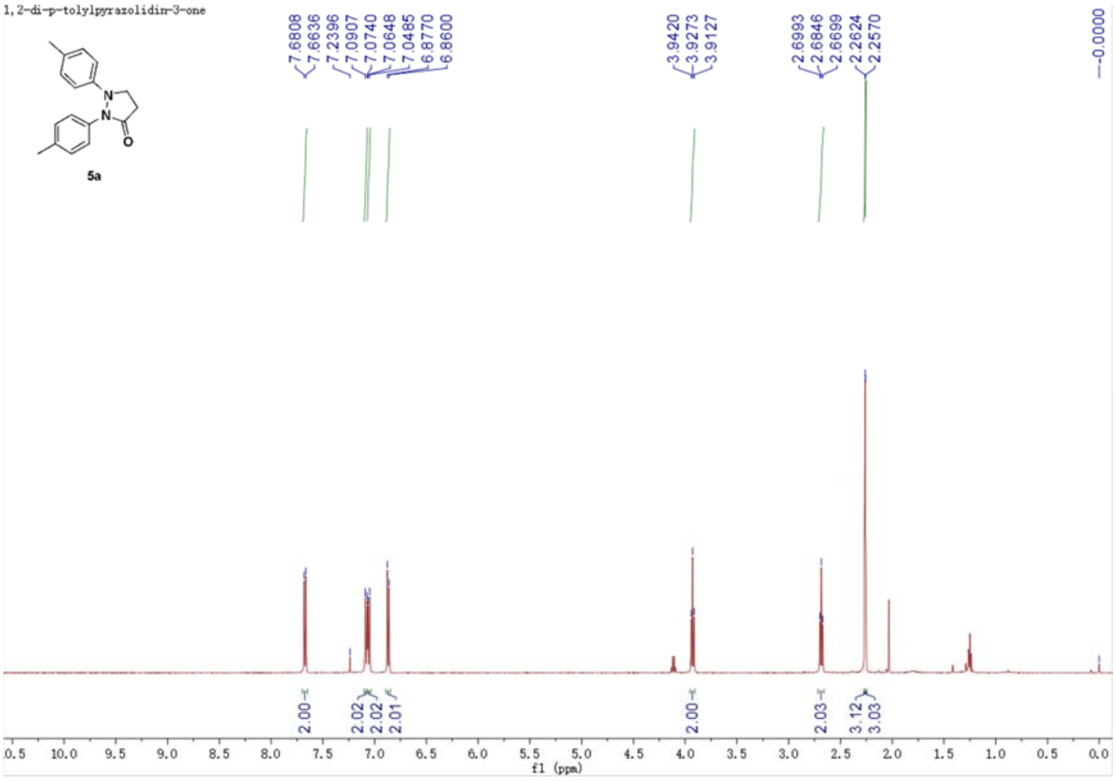
1,2-di-p-tolylpyrazolidin-3-one
5a
7.6808
7.6636
7.2396
7.0907
7.0740
7.0648
7.0485
6.8770
6.8600
3.9420
3.9273
3.9127
2.6993
2.6846
2.6699
2.2624
2.2570
0.0000
2.00
2.02
2.02
2.01
2.00
2.03
3.12
3.03
f1 (ppm)

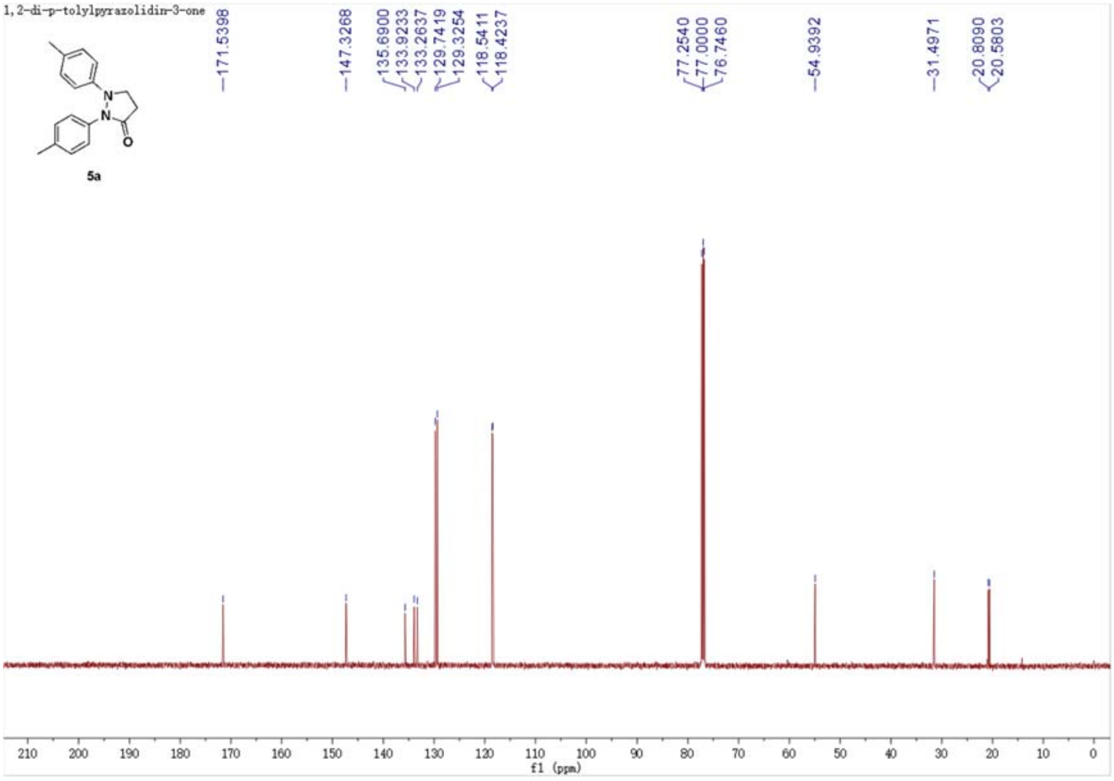
1,2-di-p-tolylpyrazolidin-3-one
5a
171.5398
147.3268
135.6900
133.9233
133.2637
129.7419
129.3254
118.5411
118.4237
77.2540
77.0000
76.7460
54.9392
31.4971
20.8090
20.5803
f1 (ppm)

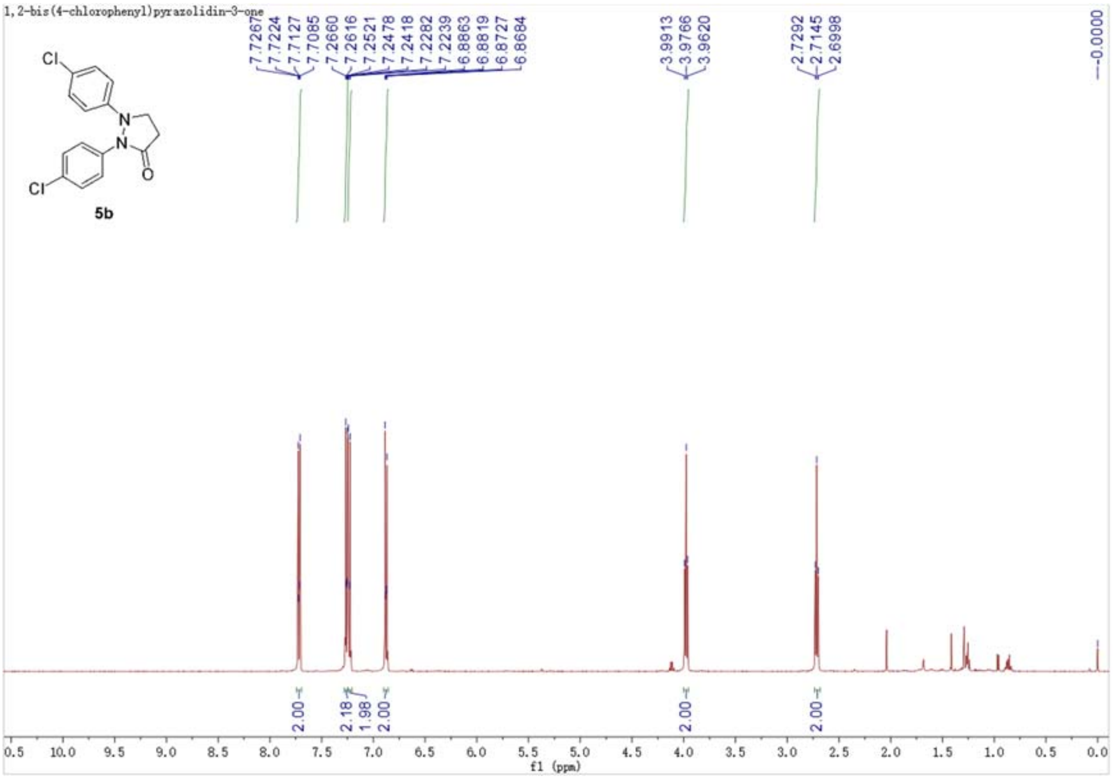
1,2-bis(4-chlorophenyl)pyrazolidin-3-one
7.7267
7.7224
7.7127
7.7085
7.2660
7.2616
7.2521
7.2478
7.2418
7.2282
7.2239
6.8863
6.8819
6.8727
6.8684
3.9913
3.9766
3.9620
2.7292
2.7145
2.6998
0.0000
Cl
N
N
O
Cl
5b
2.00
2.18
1.98
2.00
2.00
2.00
f1 (ppm)

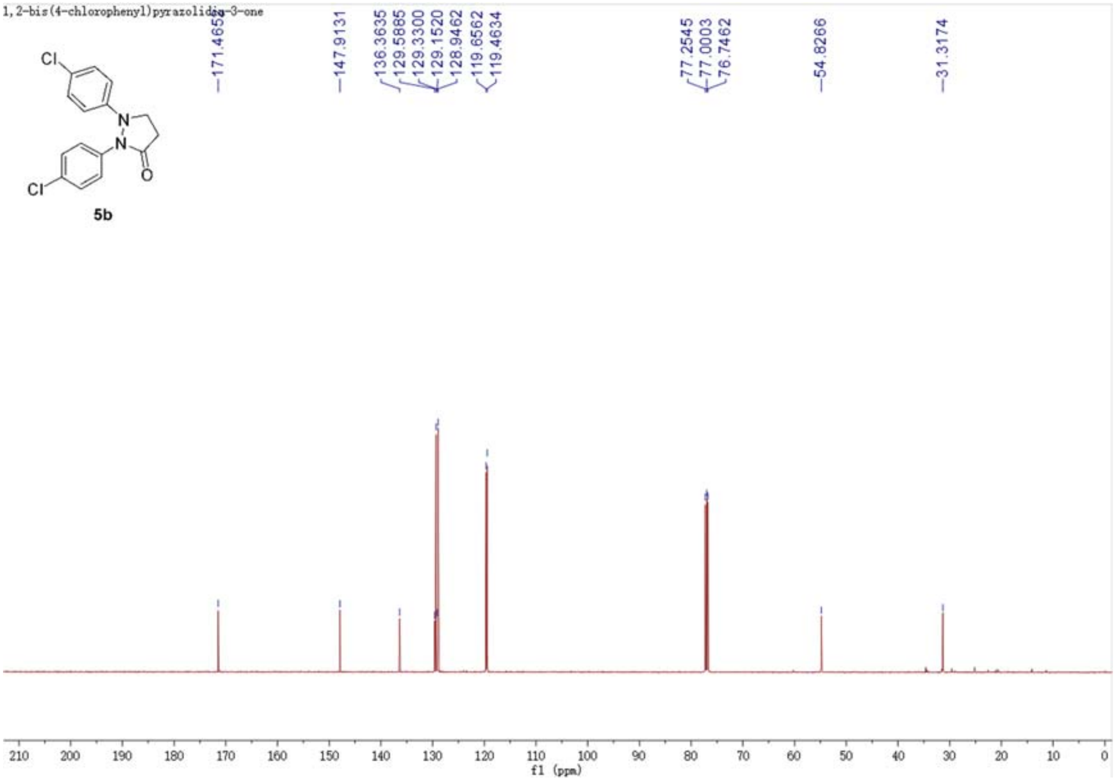
1,2-bis(4-chlorophenyl)pyrazolidin-3-one
171.4653
147.9131
136.3635
129.5885
129.3300
129.1520
128.9462
119.6562
119.4634
77.2545
77.0003
76.7462
54.8266
31.3174
Cl
N
N
O
Cl
5b
f1 (ppm)

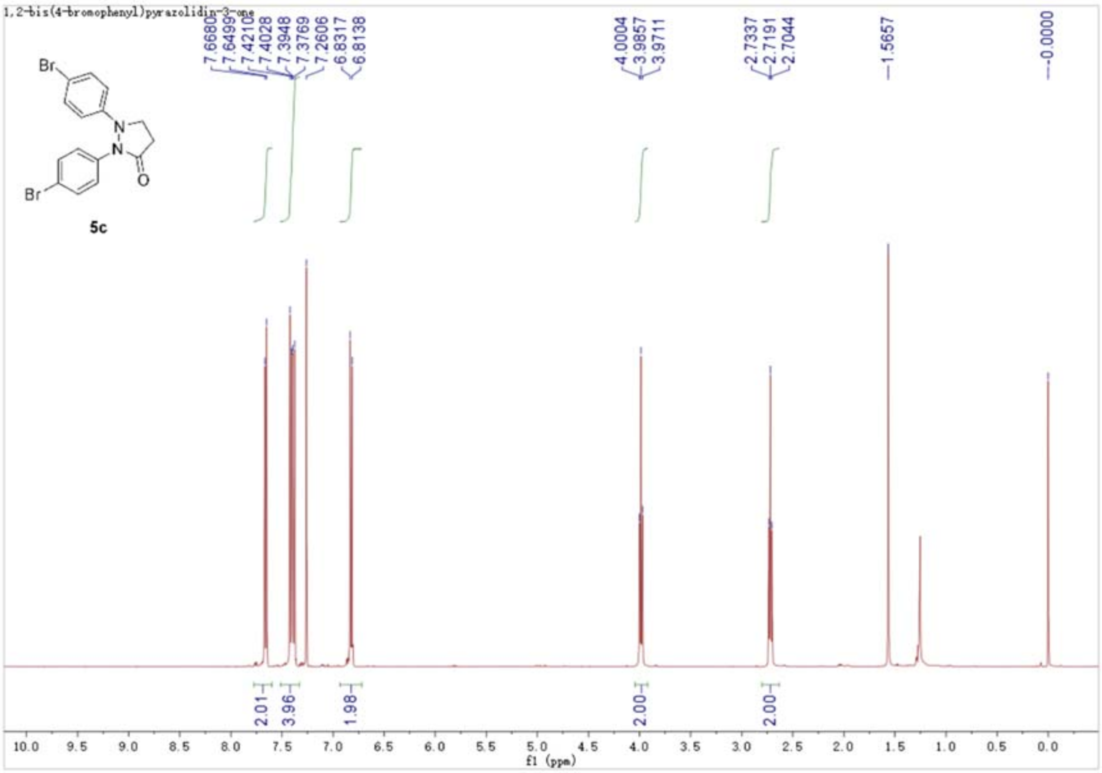
1,2-bis(4-bromophenyl)pyrazolidin-3-one
7.6680
7.6499
7.4210
7.4028
7.3948
7.3769
7.2606
6.8317
6.8138
4.0004
3.9857
3.9711
2.7337
2.7191
2.7044
1.5657
0.0000
Br
N
N
O
Br
5c
2.01
3.96
1.98
2.00
2.00
10.0 9.5 9.0 8.5 8.0 7.5 7.0 6.5 6.0 5.5 5.0 4.5 4.0 3.5 3.0 2.5 2.0 1.5 1.0 0.5 0.0
f1 (ppm)

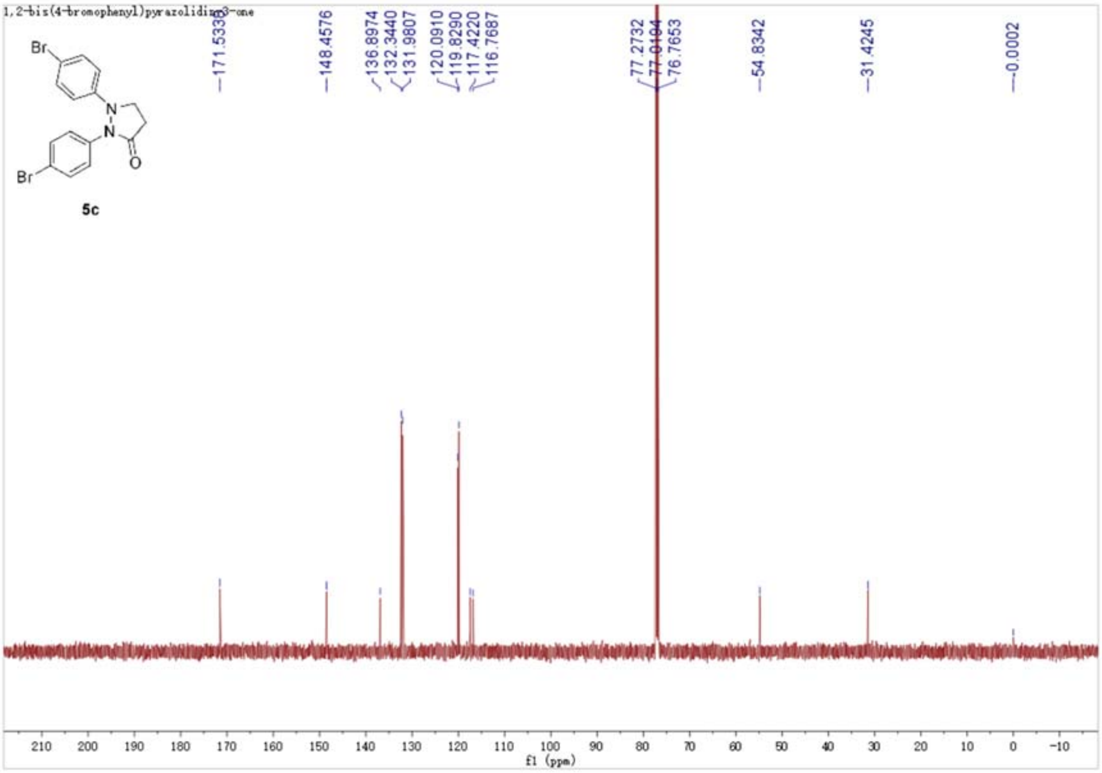
1,2-bis(4-bromophenyl)pyrazolidin-3-one
171.5338
148.4576
136.8974
132.3440
131.9807
120.0910
119.8290
117.4220
116.7687
77.2732
77.0194
76.7653
54.8342
31.4245
0.0002
Br
N
N
O
Br
5c
210 200 190 180 170 160 150 140 130 120 110 100 90 80 70 60 50 40 30 20 10 0 -10
f1 (ppm)

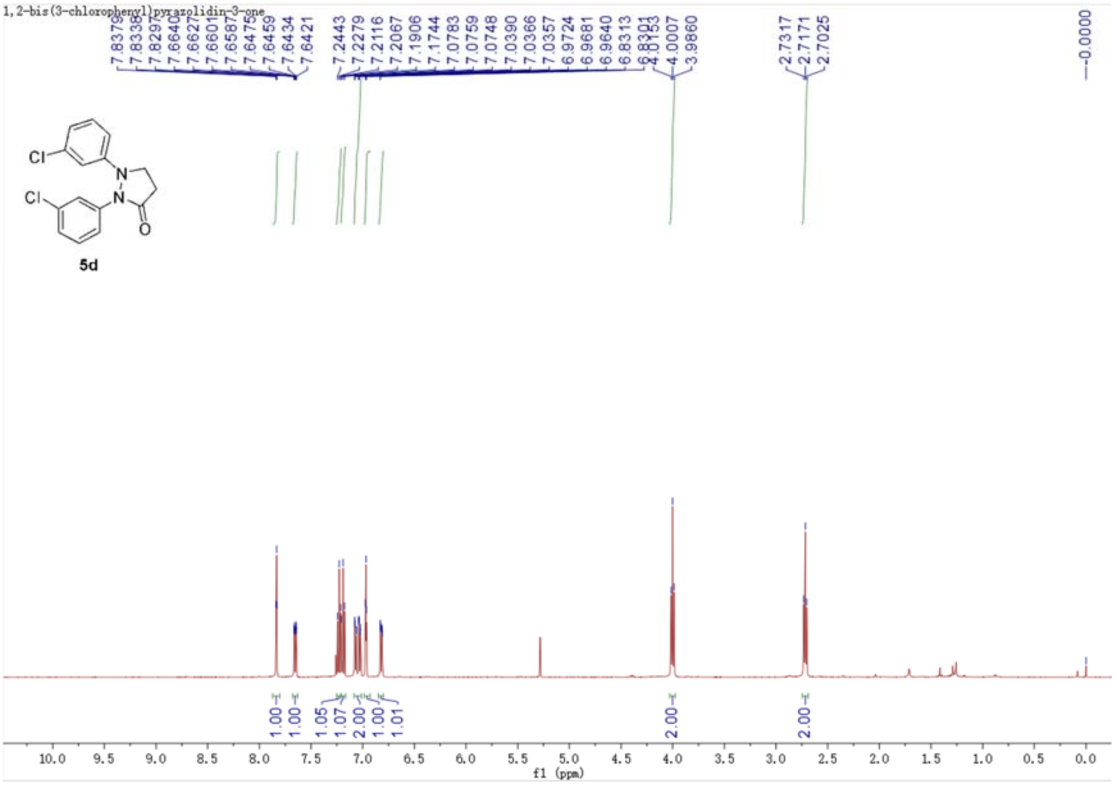
1,2-bis(3-chlorophenyl)pyrazolidin-3-one
5d
f1 (ppm)

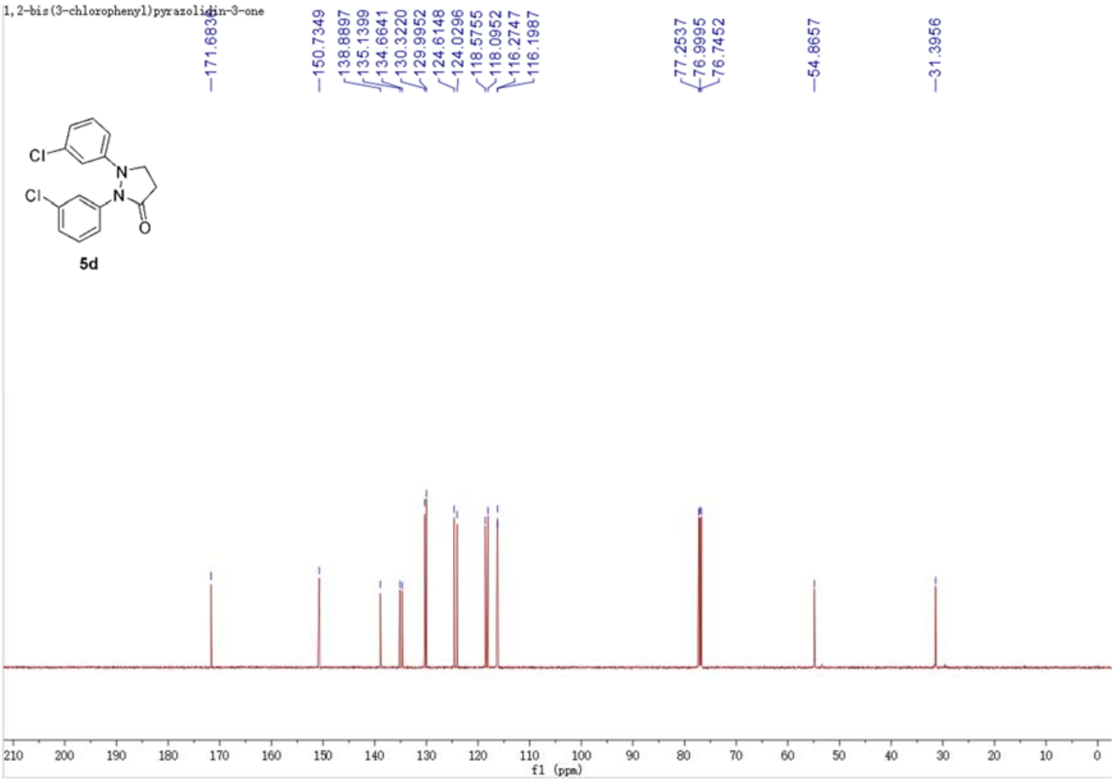
1,2-bis(3-chlorophenyl)pyrazolidin-3-one
5d
f1 (ppm)

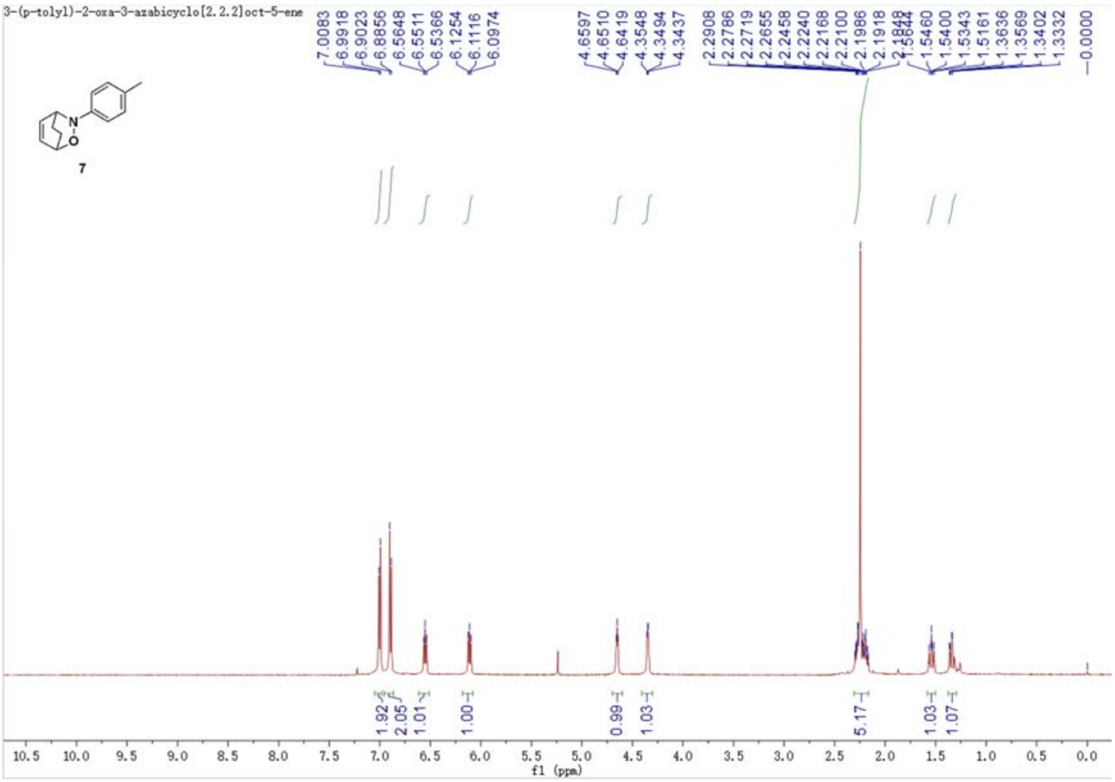
3-(p-tolyl)-2-oxa-3-azabicyclo[2.2.2]oct-5-ene
7
7.0083 6.9918 6.9023 6.8856 6.5648 6.5511 6.5366 6.1254 6.1116 6.0974
4.6597 4.6510 4.6419 4.3548 4.3494 4.3437
2.2908 2.2786 2.2719 2.2655 2.2458 2.2240 2.2168 2.2100 2.1986 2.1918 [illegible]
1.5460 1.5400 1.5343 1.5161 1.3636 1.3569 1.3402 1.3332
0.0000
1.92 2.05 1.01 1.00 0.99 1.03 5.17 1.03 1.07
f1 (ppm)

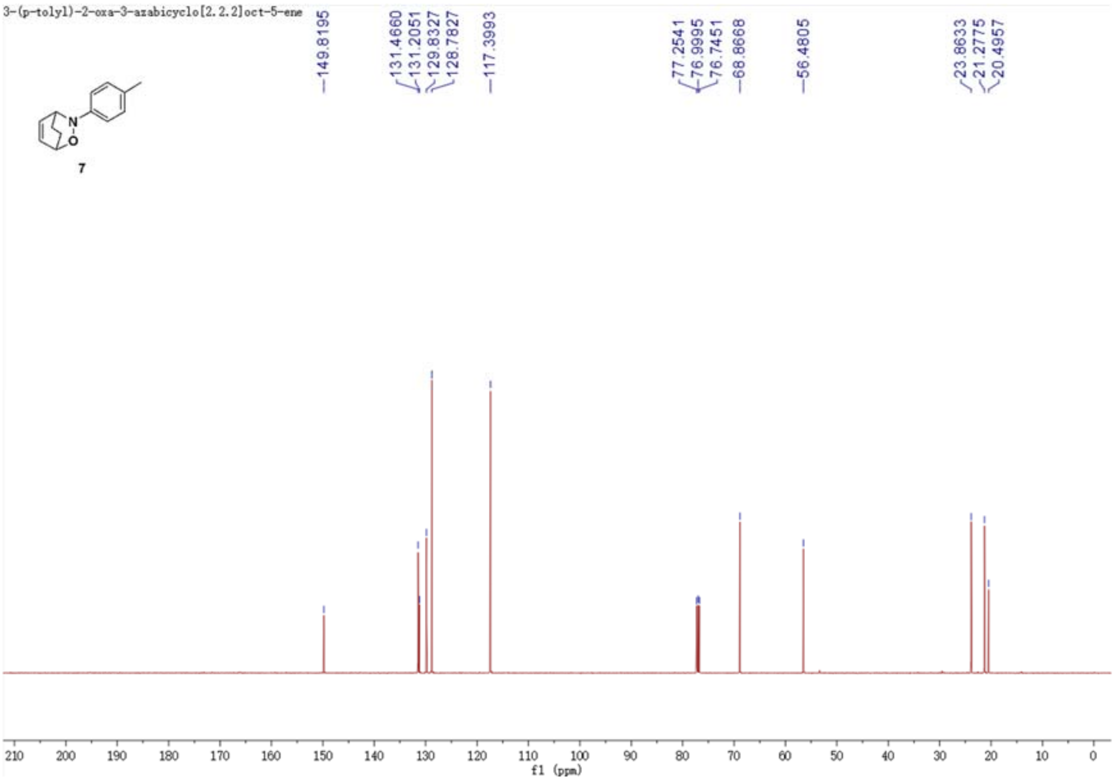
3-(p-tolyl)-2-oxa-3-azabicyclo[2.2.2]oct-5-ene
7
149.8195
131.4660 131.2051 129.8327 128.7827
117.3993
77.2541 76.9995 76.7451
68.8668
56.4805
23.8633 21.2775 20.4957
f1 (ppm)

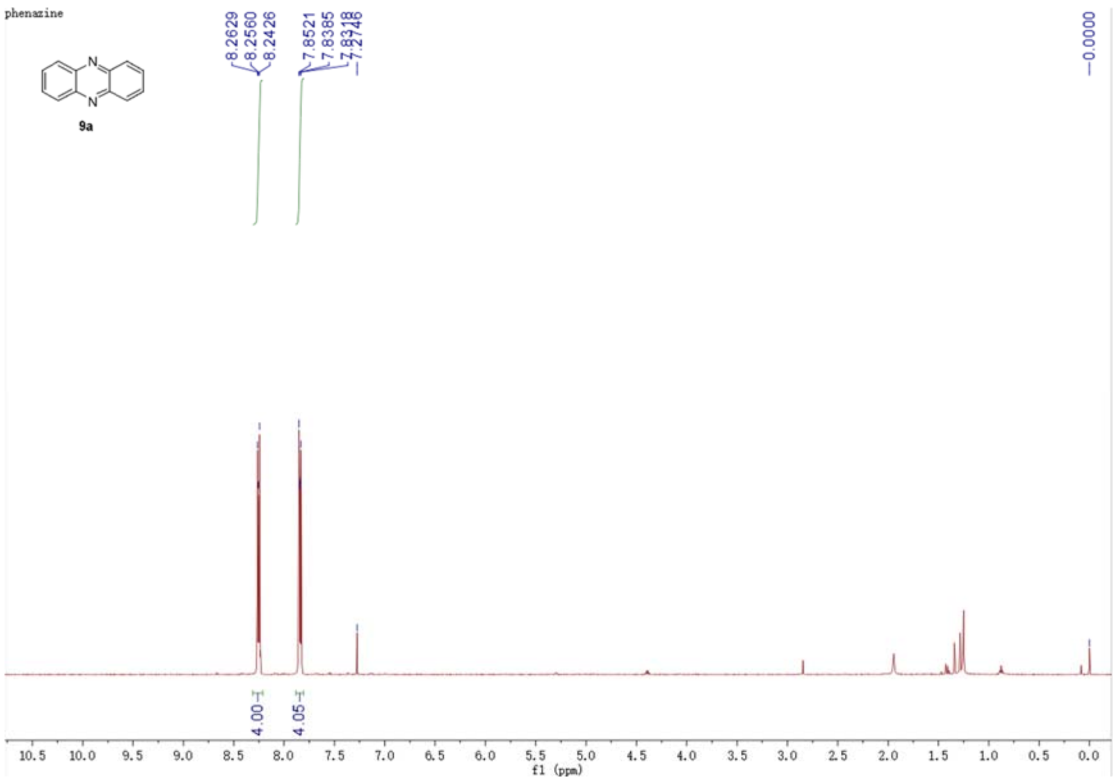
phenazine
9a
8.2629
8.2560
8.2426
7.8521
7.8385
7.8318
7.2746
0.0000
4.00
4.05
10.5 10.0 9.5 9.0 8.5 8.0 7.5 7.0 6.5 6.0 5.5 5.0 4.5 4.0 3.5 3.0 2.5 2.0 1.5 1.0 0.5 0.0
f1 (ppm)

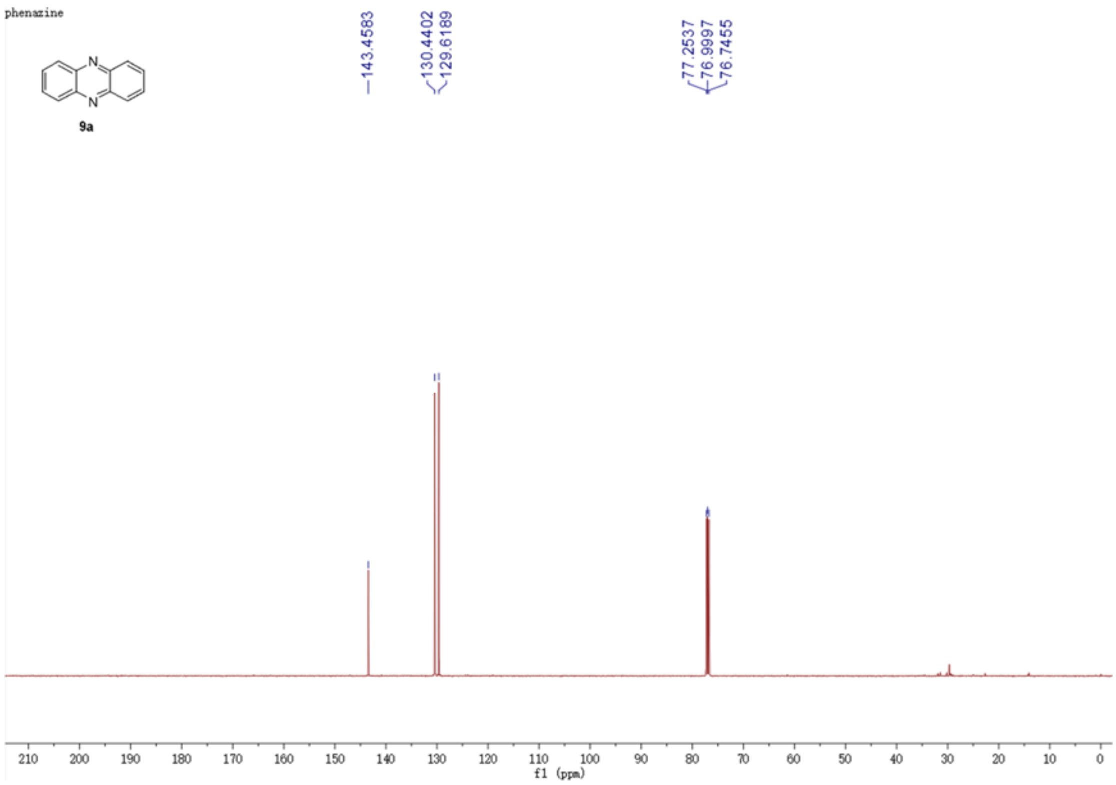
phenazine
9a
143.4583
130.4402
129.6189
77.2537
76.9997
76.7455
210 200 190 180 170 160 150 140 130 120 110 100 90 80 70 60 50 40 30 20 10 0
f1 (ppm)

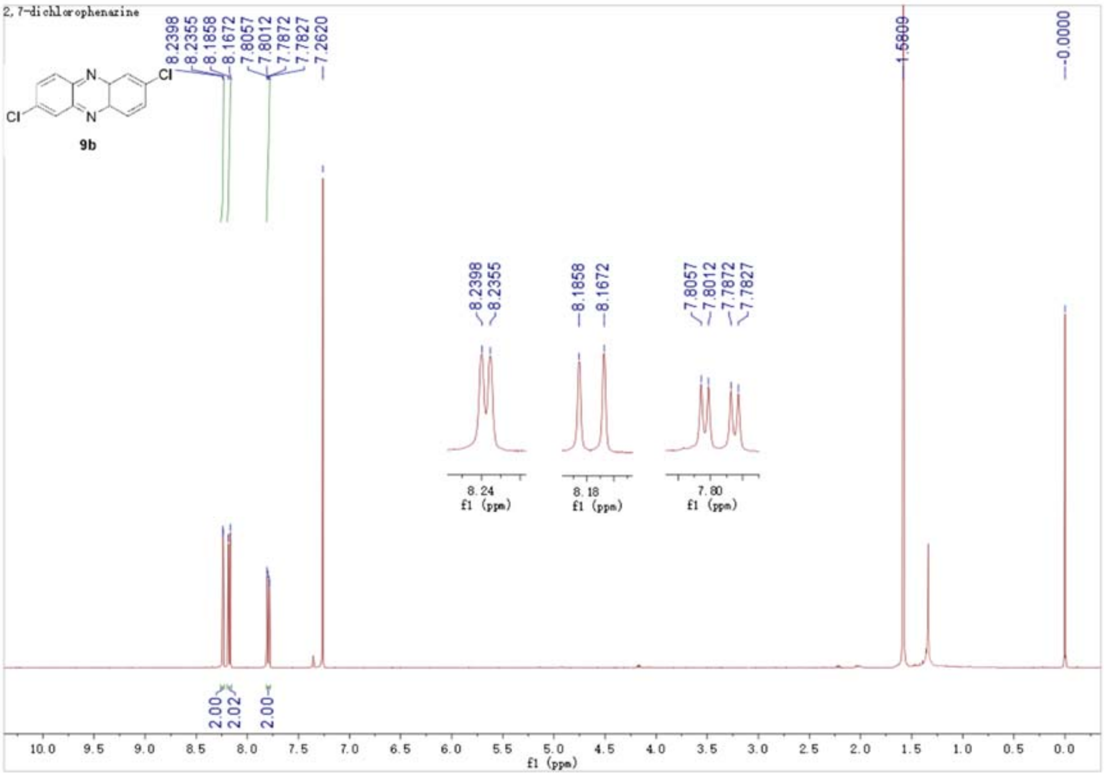
2,7-dichlorophenazine
8.2398
8.2355
8.1858
8.1672
7.8057
7.8012
7.7872
7.7827
7.2620
1.5809
0.0000
9b
2.00
2.02
2.00
f1 (ppm)

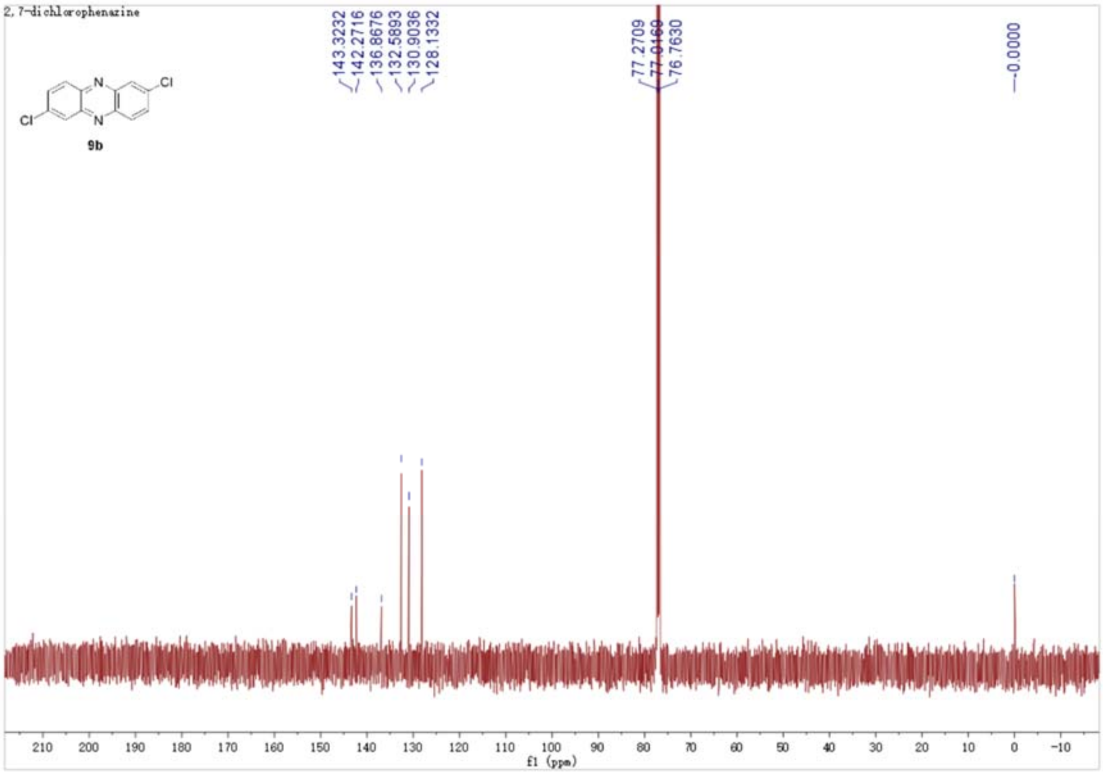
2,7-dichlorophenazine
143.3232
142.2716
136.8676
132.5893
130.9036
128.1332
77.2709
77.0169
76.7630
0.0000
9b
f1 (ppm)

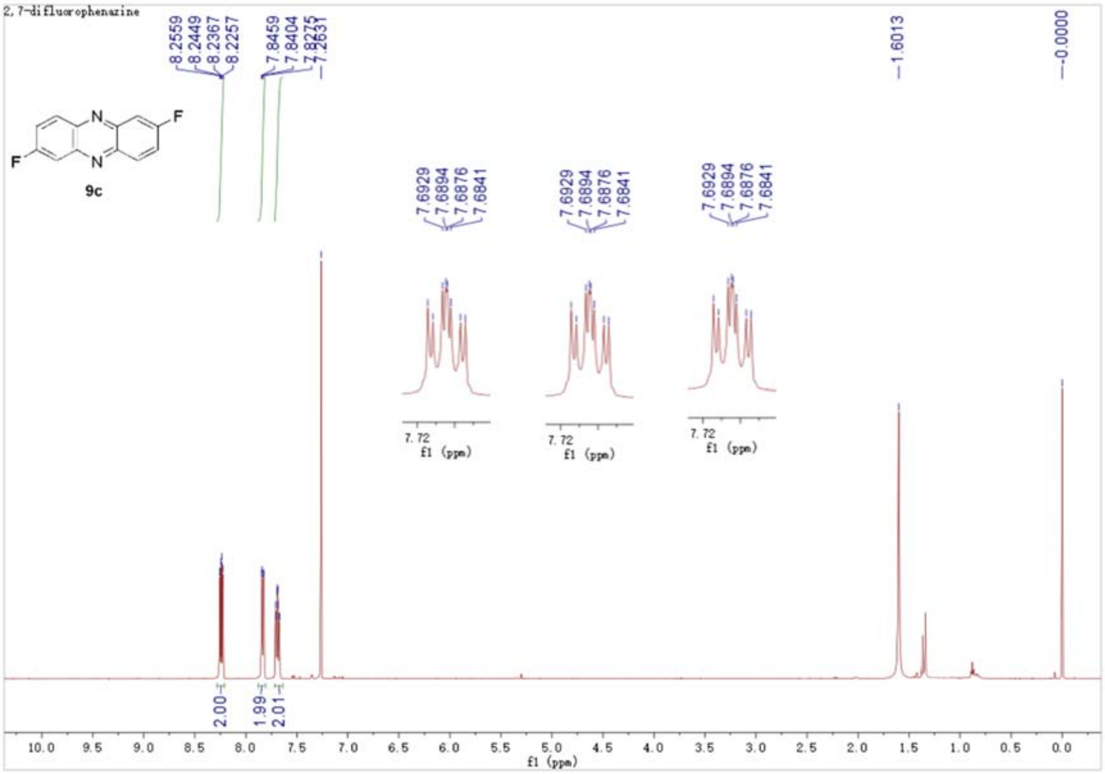
2,7-difluorophenazine
8.2559
8.2449
8.2367
8.2257
7.8459
7.8404
7.6929
7.6894
7.6876
7.6841
-1.6013
-0.0000
9c
2.00
1.99
2.01
f1 (ppm)

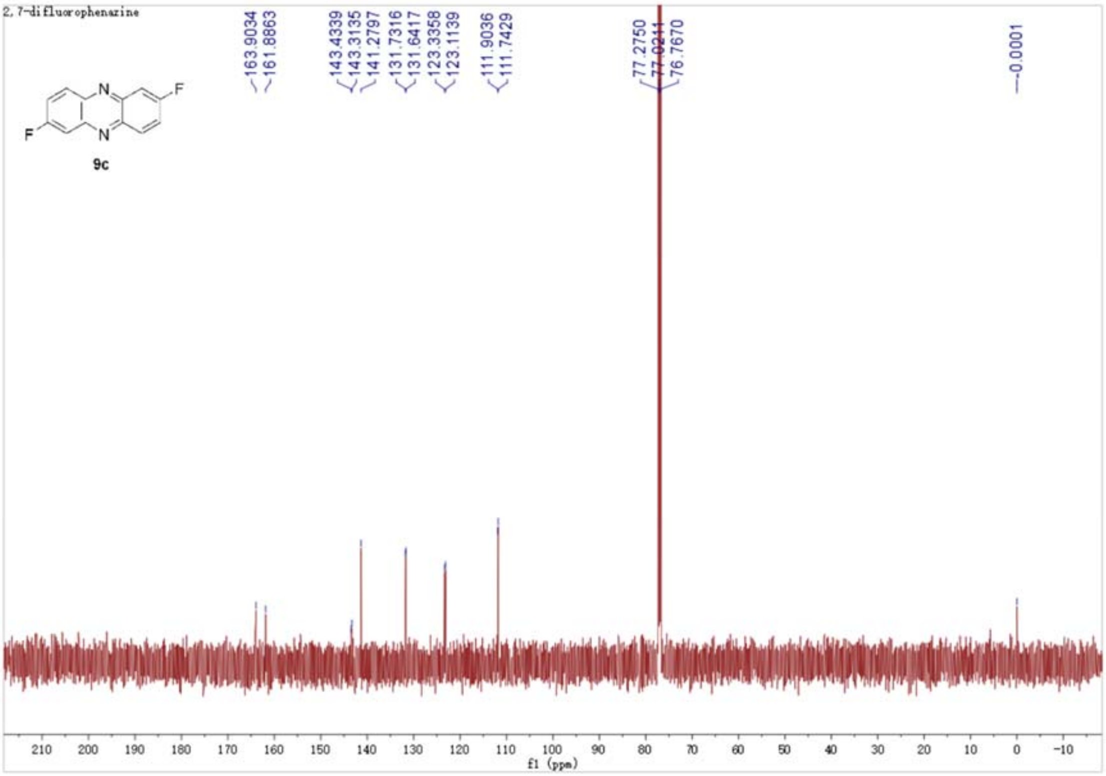
2,7-difluorophenazine
163.9034
161.8863
143.4339
143.3135
141.2797
131.7316
131.6417
123.3358
123.1139
111.9036
111.7429
77.2750
76.7670
-0.0001
9c
f1 (ppm)

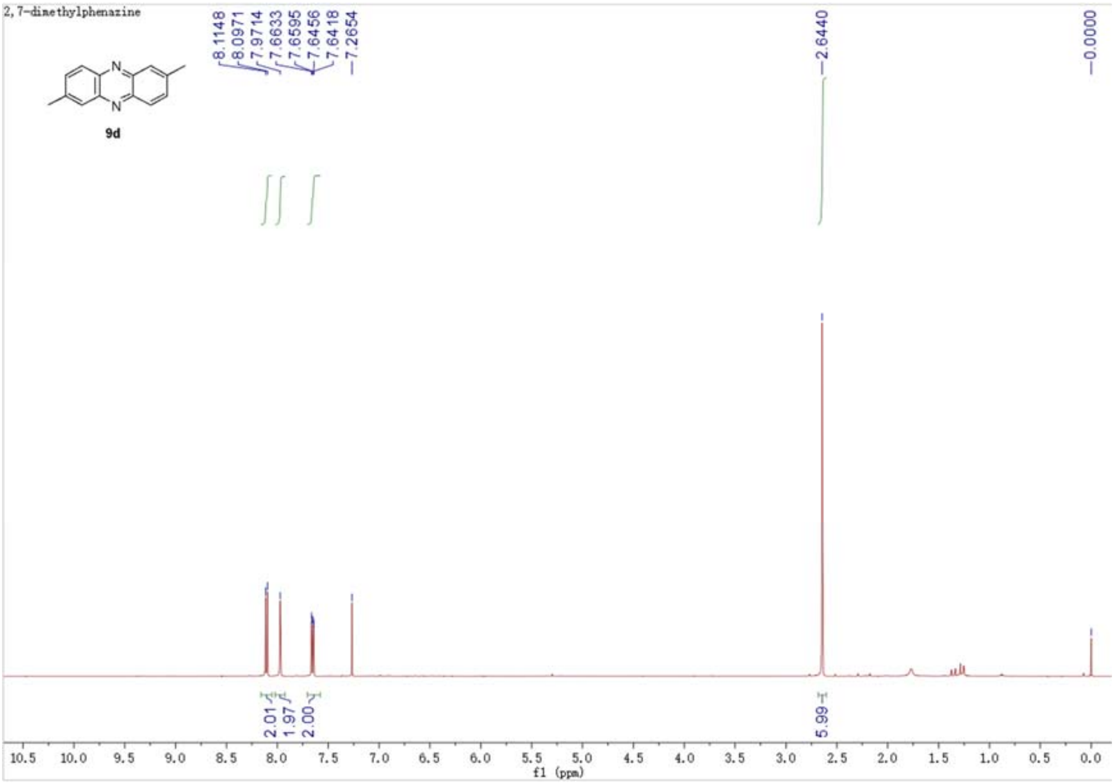
2,7-dimethylphenazine
9d
8.1148
8.0971
7.9714
7.6633
7.6595
7.6456
7.6418
7.2654
2.6440
0.0000
2.01
1.97
2.00
5.99
10.5 10.0 9.5 9.0 8.5 8.0 7.5 7.0 6.5 6.0 5.5 5.0 4.5 4.0 3.5 3.0 2.5 2.0 1.5 1.0 0.5 0.0
f1 (ppm)

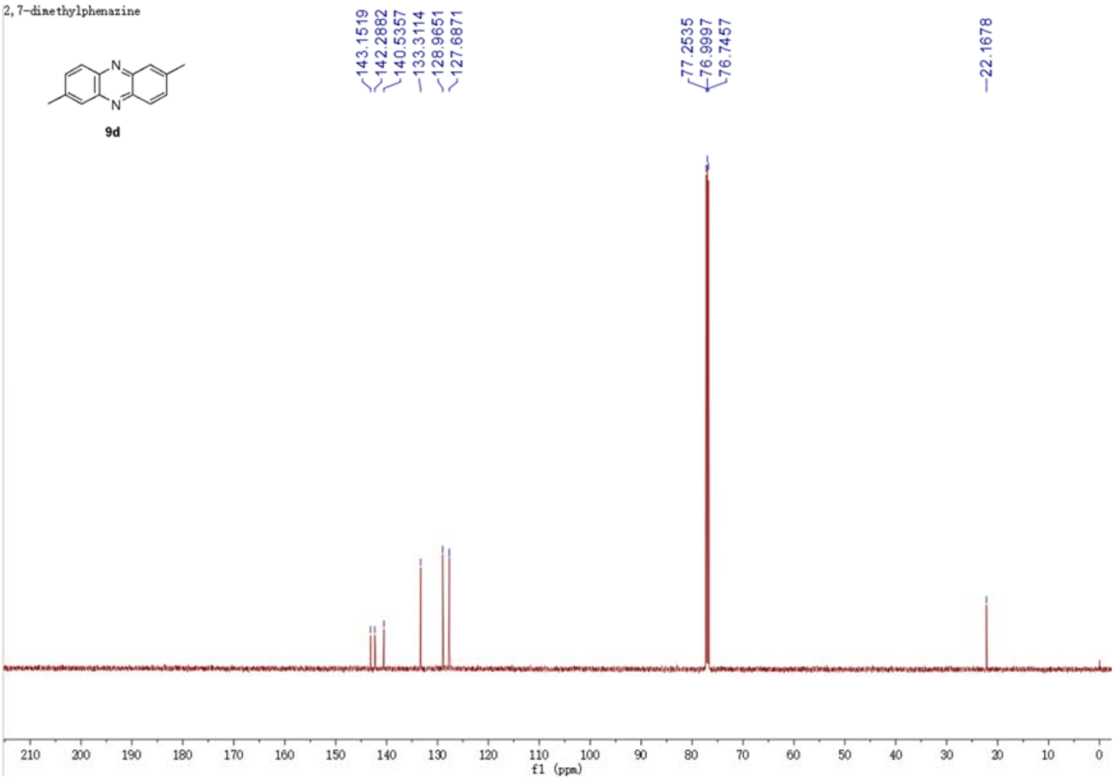
2,7-dimethylphenazine
9d
143.1519
142.2882
140.5357
133.3114
128.9651
127.6871
77.2535
76.9997
76.7457
22.1678
210 200 190 180 170 160 150 140 130 120 110 100 90 80 70 60 50 40 30 20 10 0
f1 (ppm)

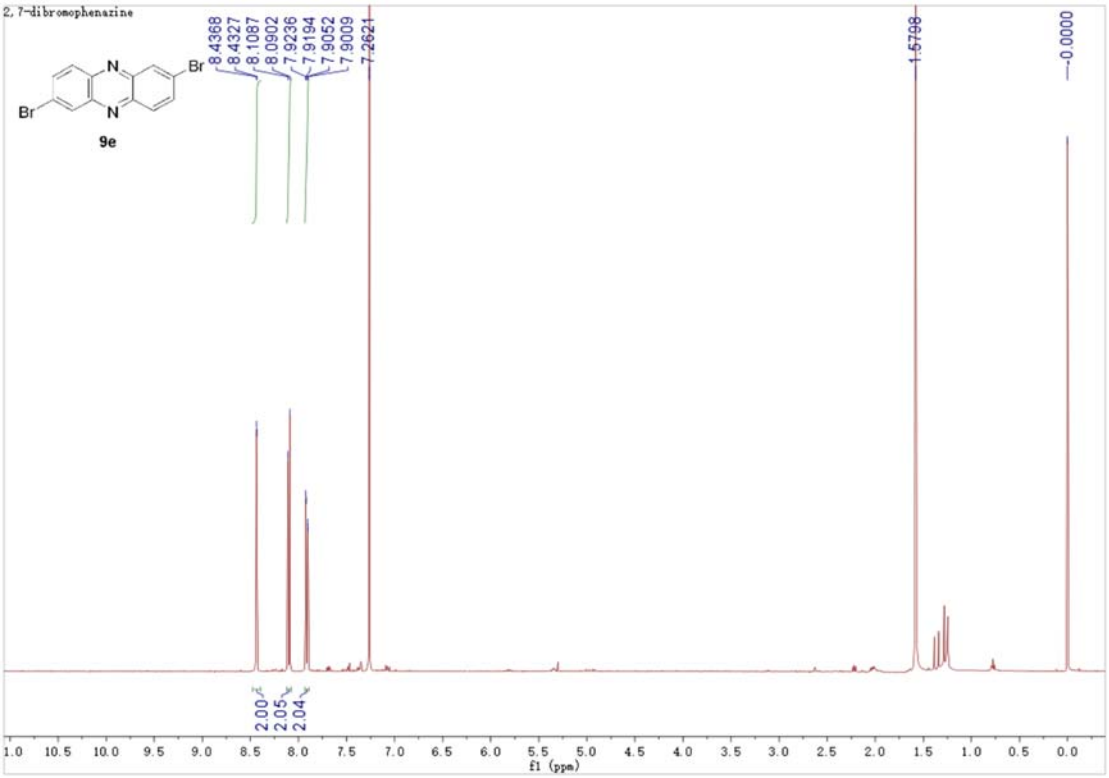
2,7-dibromophenazine
9e
8.4368
8.4327
8.1087
8.0902
7.9236
7.9194
7.9052
7.9009
7.2621
1.5798
0.0000
2.00
2.05
2.04
f1 (ppm)

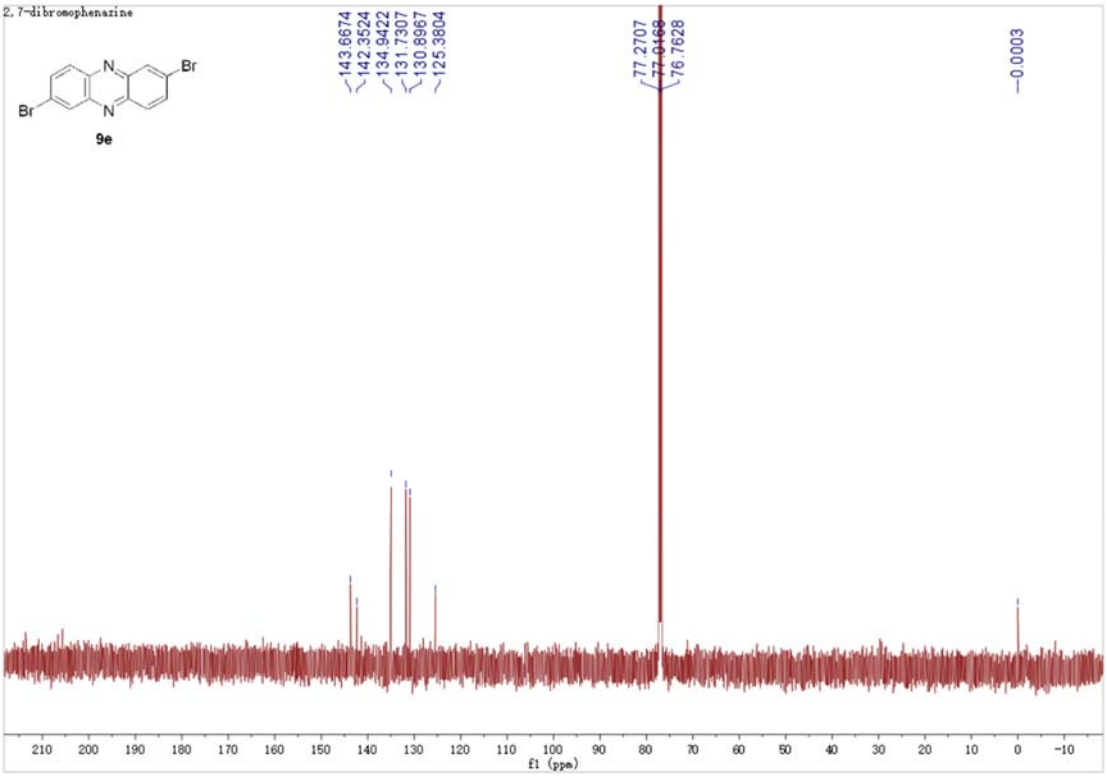
2,7-dibromophenazine
9e
143.6674
142.3524
134.9422
131.7307
130.8967
125.3804
77.2707
77.0168
76.7628
-0.0003
f1 (ppm)

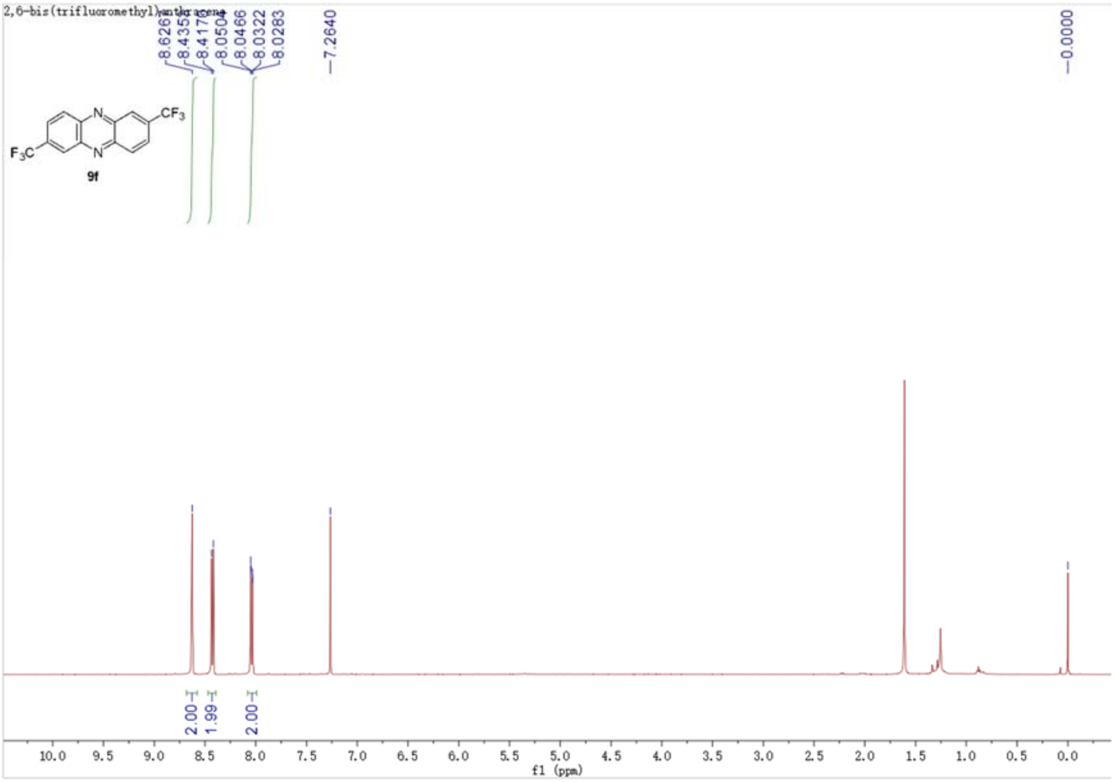
2,6-bis(trifluoromethyl)anthracene
8.6261
8.4355
8.4171
8.0504
8.0466
8.0322
8.0283
7.2640
0.0000
9f
2.00
1.99
2.00
f1 (ppm)

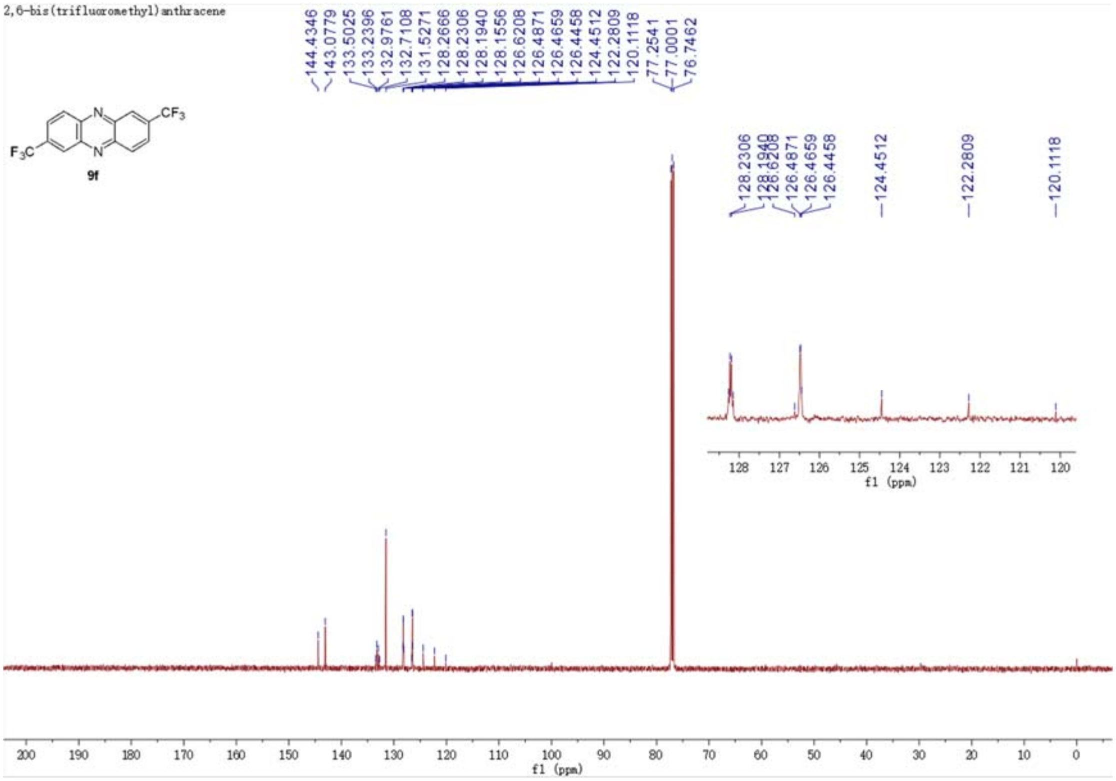
2,6-bis(trifluoromethyl)anthracene
144.4346
143.0779
133.5025
133.2396
132.9761
132.7108
131.5271
128.2666
128.2306
128.1940
128.1556
126.6208
126.4871
126.4659
126.4458
124.4512
122.2809
120.1118
77.2541
77.0001
76.7462
9f
f1 (ppm)

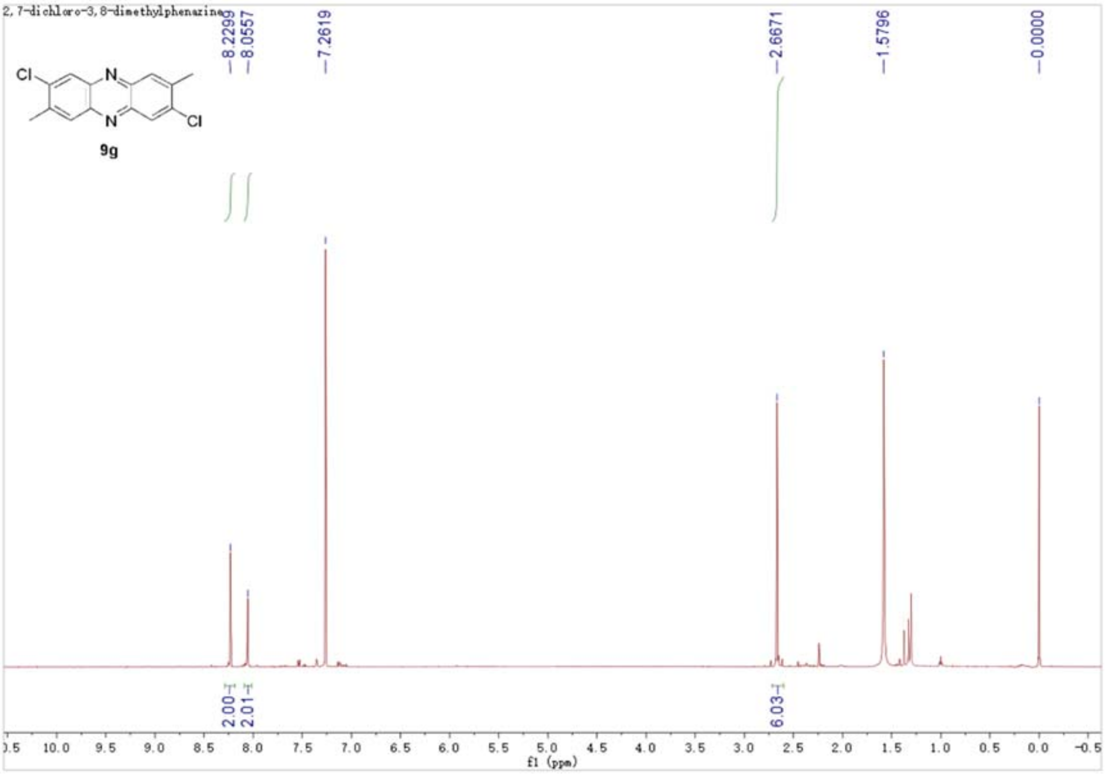
2,7-dichloro-3,8-dimethylphenazine
9g
8.2299
8.0557
7.2619
2.6671
1.5796
0.0000
2.00
2.01
6.03
f1 (ppm)

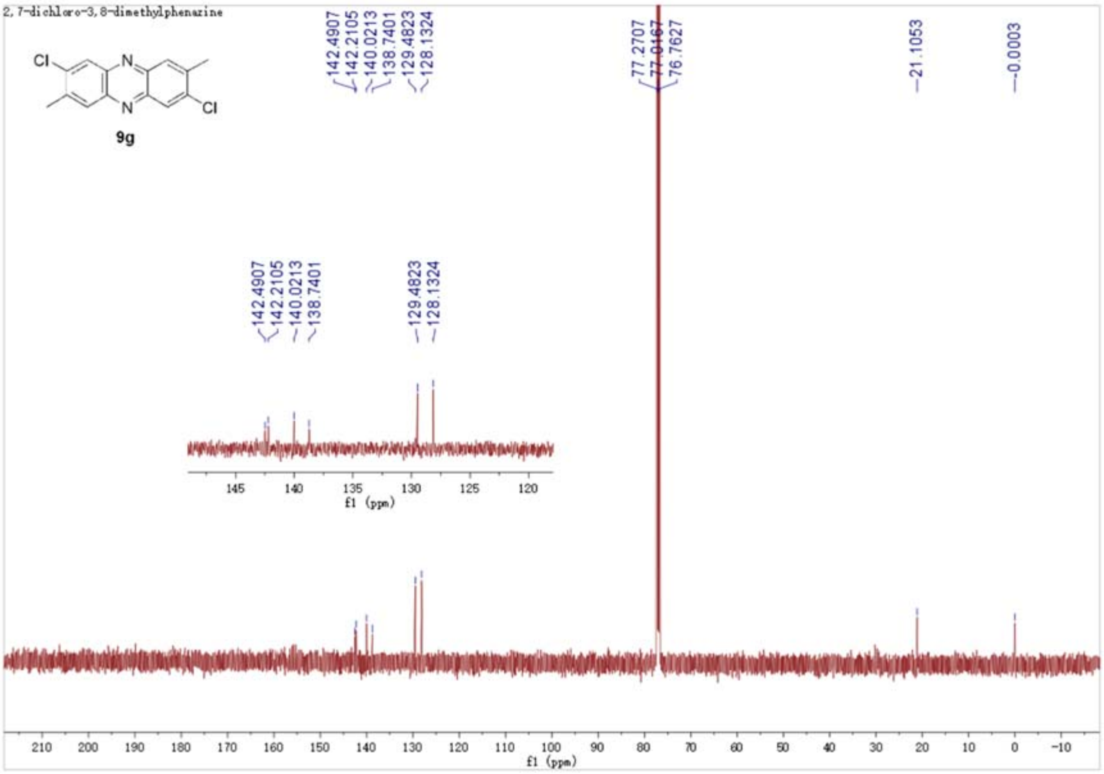
2,7-dichloro-3,8-dimethylphenazine
9g
142.4907
142.2105
140.0213
138.7401
129.4823
128.1324
77.2707
77.0167
76.7627
21.1053
-0.0003
f1 (ppm)

4 Copies of ^{19}F NMR Spectra of 2b, 3b, 9c and 9f

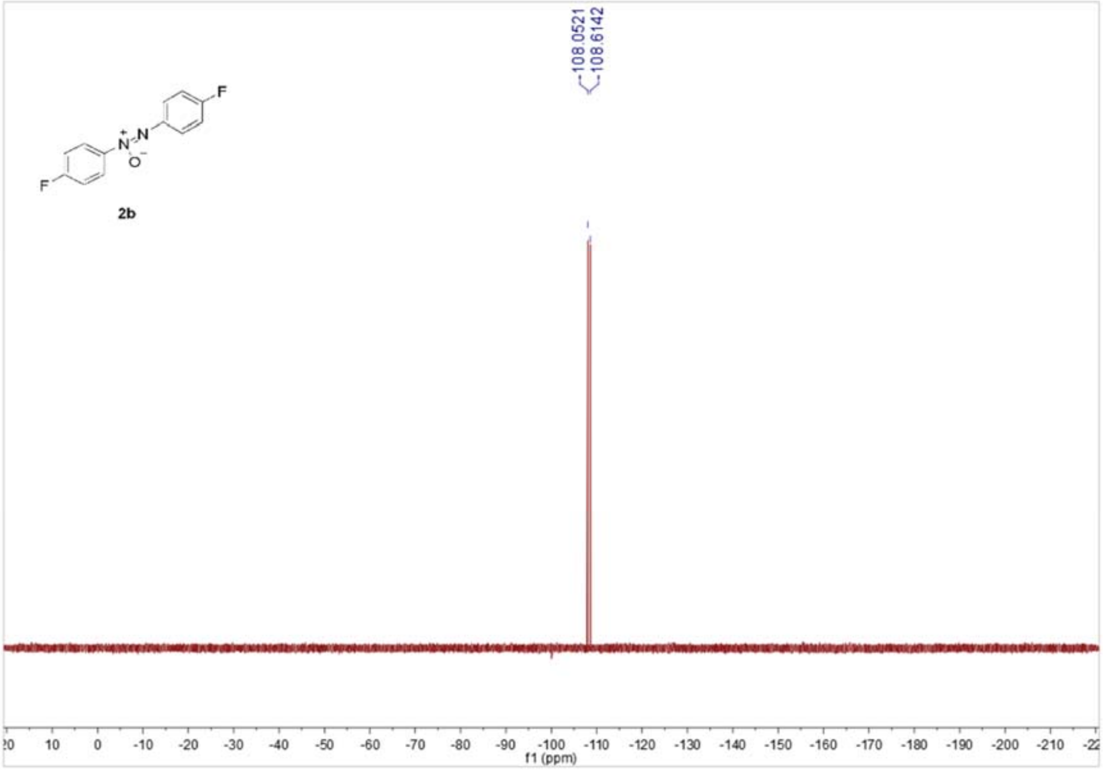

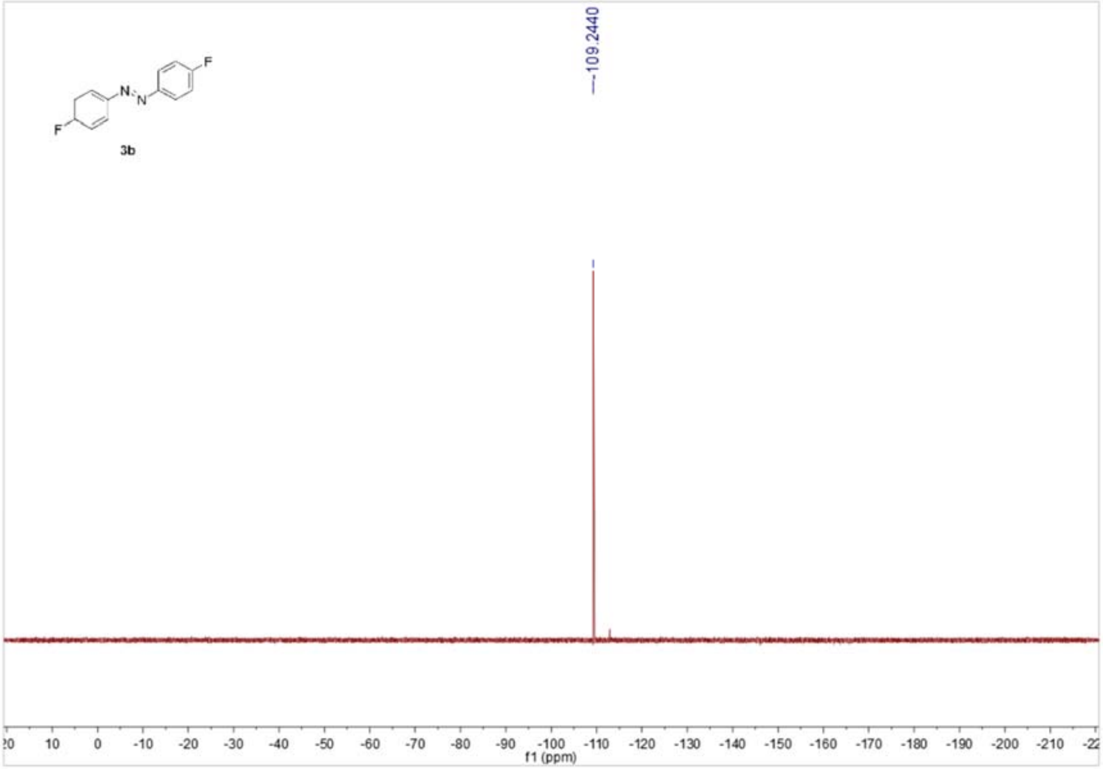

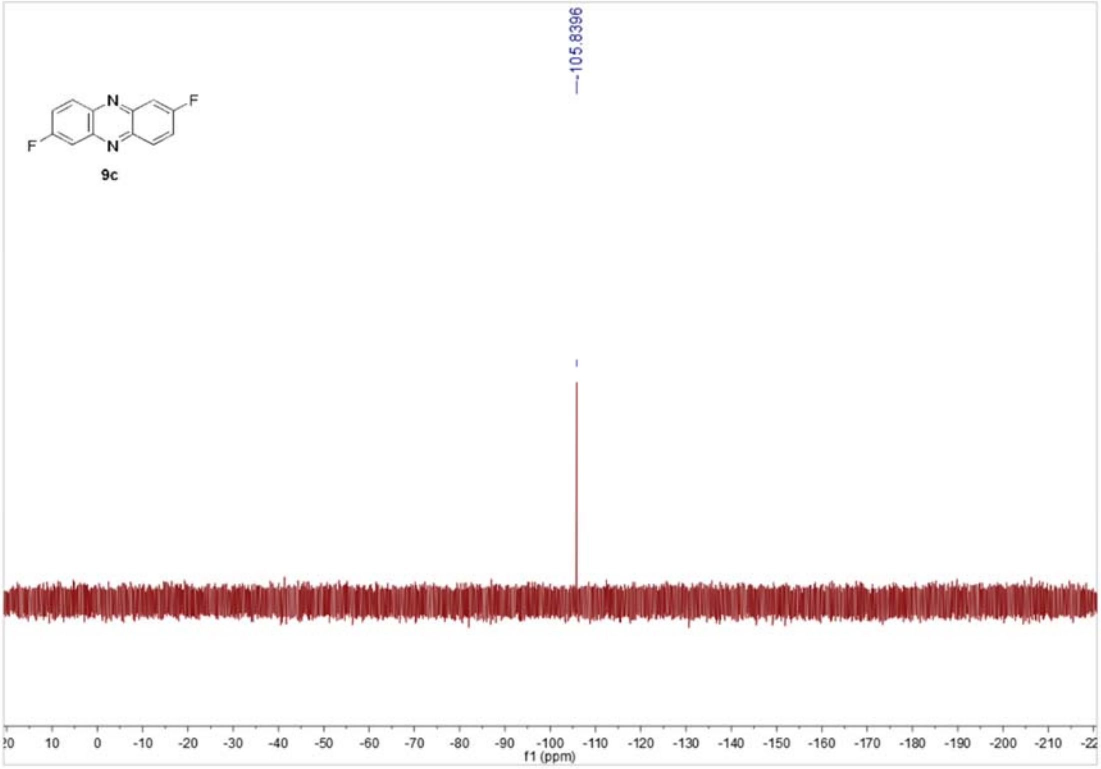
-105.8396
9c
f1 (ppm)

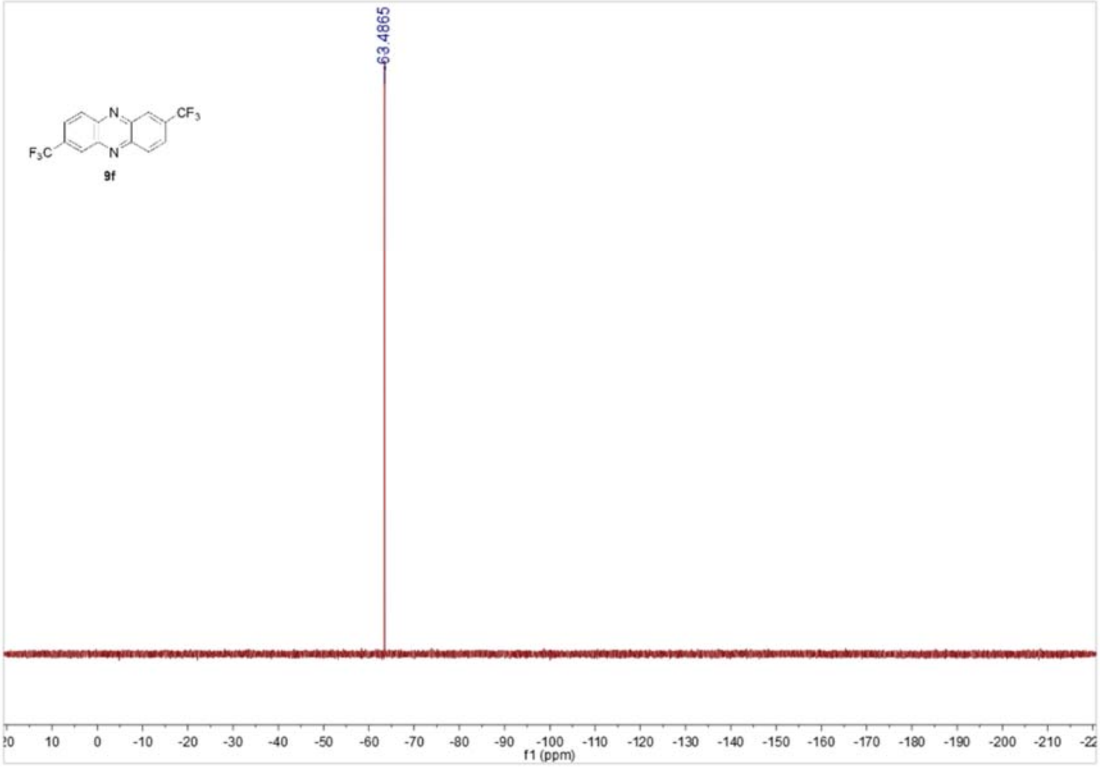
-63.4865
9f
f1 (ppm)

References

1 I. F. Fernández, L. Hecquet and W. Fessner, *Adv. Synth. Catal.,* **2022**, *364*, 612-621.

2 R. P. Wei and F. Shi, *Synth. Commun.,* **2019**, *49*, 688-696.

3 J. H. Kim, J. H. Park, Y. K. Chung and K. H. Park, *Adv. Synth. Catal.,* **2012**, *354*, 2412-2418.

4 D. Azarifar, S. M. Khatami and Z. Najminejad, *J. Iran. Chem. Soc.,* **2014**, *11*, 587-592.5 K.

5 Nozawa-Kumada, E. Abe, S. Ito, M. Shigeno and Y. Kondo, *Org. Biomol. Chem.,* **2018**, *16*, 3095-3098.

6 S. H. Gund, R. S. Shelkar and J. M. Nagarkar, *RSC Advances,* **2014**, *4*, 42947-42951.

7 N. Sakai, K. Fujii, S. Nabeshima, R. Ikeda and T. Konakahara, *Chem. Commun.,* **2010**, *46*, 3173-3175.

8 C. C. Changa, S. Caoa, S. Kanga, L. Kai, X. Tian, P. Pandey and R. B. Silverman, *Bioorg. Med. Chem.,* **2010**, *18*, 3147-3158.

9 W. Wang, Y. Wang, Y. Yang, S. Xie, Q. Wang, W. Chen and Y. Shao, *J. Org. Chem.,* **2024**, *89*, 9265-9274.

10 S. A. Mokbel, R. K. Fathalla, L. Y. El-Sharkawy, A. H. Abadi, M. Engel and M. Abdel-Halim, *Bioorg. Chem.,* **2020**, *99*, 103759.

11 R. A. Wagdy, N. S. Abutaleb, R. K. Fathalla, Y. Elgammal, S. Weck, R. Pal and M. Abdel-Halim, *Eur. J. Med. Chem.,* **2023**, *261*, 115789.

12 X. W. Liu, N. Y. Liu, Y. Q. Deng, S. Wang, T. Liu, Y. C. Tang and J. L. Lu, *J. Biomol. Struct. Dyn.,* **2021**, *39*, 5953-5962.

13 L. Yu, X. Zhou, D. Wu and H. Xiang, *J. Organomet. Chem.* **2012**, *705*, 75-78.
